# Preparation and characterization of manganese, cobalt and zinc DNA nanoflowers with tuneable morphology, DNA content and size

**DOI:** 10.1093/nar/gky630

**Published:** 2018-07-12

**Authors:** Ysobel R Baker, Jinfeng Chen, Jason Brown, Afaf H El-Sagheer, Philip Wiseman, Errin Johnson, Paul Goddard, Tom Brown

**Affiliations:** 1 Department of Chemistry, University of Oxford, Oxford, Oxfordshire OX1 3TA, UK; 2 Department of Physics, University of Oxford, Oxford, Oxfordshire OX1 3PU, UK; 3 Chemistry Branch, Faculty of Petroleum and Mining Engineering, Suez University, Suez 43721, Egypt; 4 Sir William Dunn School of Pathology, University of Oxford, Oxford, Oxfordshire OX1 3RE, UK; 5 Department of Physics, University of Warwick, Coventry, Warwickshire CV4 7AL, UK

## Abstract

Recently reported DNA nanoflowers are an interesting class of organic-inorganic hybrid materials which are prepared using DNA polymerases. DNA nanoflowers combine the high surface area and scaffolding of inorganic Mg_2_P_2_O_7_ nanocrystals with the targeting properties of DNA, whilst adding enzymatic stability and enhanced cellular uptake. We have investigated conditions for chemically modifying the inorganic core of these nanoflowers through substitution of Mg^2+^ with Mn^2+^, Co^2+^ or Zn^2+^ and have characterized the resulting particles. These have a range of novel nanoarchitectures, retain the enzymatic stability of their magnesium counterparts and the Co^2+^ and Mn^2+^ DNA nanoflowers have added magnetic properties. We investigate conditions to control different morphologies, DNA content, hybridization properties, and size. Additionally, we show that DNA nanoflower production is not limited to Ф29 DNA polymerase and that the choice of polymerase can influence the DNA length within the constructs. We anticipate that the added control of structure, size and chemistry will enhance future applications.

## INTRODUCTION

State-of-the art materials with increasingly sophisticated properties are at the forefront of scientific research and modern technology. Hybrid materials aim to merge the best properties of the biological, organic and inorganic components, whilst instilling new functionality not otherwise possible (1). By coupling biological and inorganic materials it is possible to overcome their associated limitations such as instability and lack of biocompatibility (2). For example, hybrid materials that combine magnetic materials with biological components have found a wide range of biomedical applications (3,4). They can be directed to a particular location using an external magnetic field to deliver therapeutics (5,6), used to generate heat locally (7) or combined with biological molecules such as antibodies, enabling magnetic capture and isolation of materials (8). Alternatively, magnetic materials can be combined with enzymes to produce enzyme nanogels which are stable, catalytically active, and can be readily recovered and reused (9).

**Figure 1.**
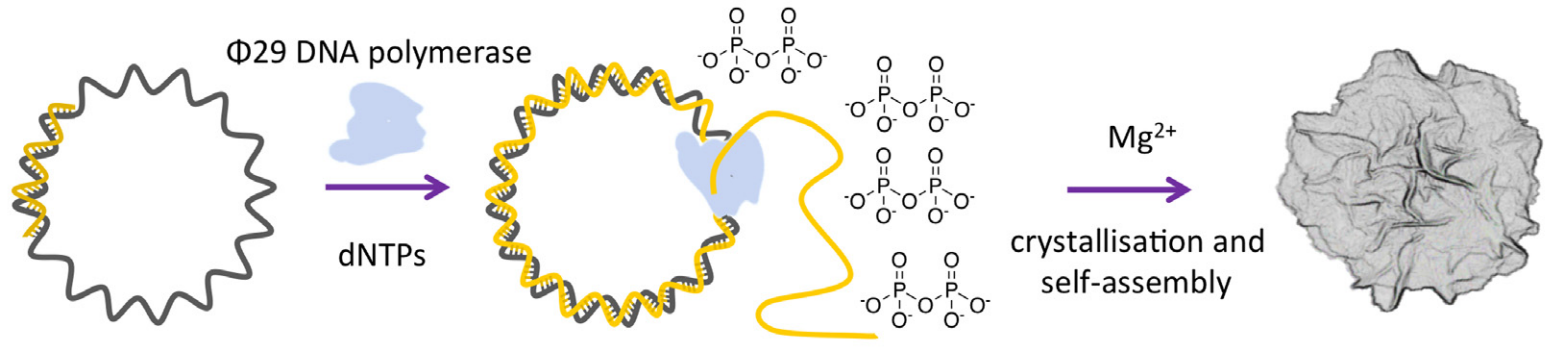
The self-assembly of DNFs using DNA polymerase enzymes. The grey circle represents a cyclic DNA template and the splint that is elongated is shown in yellow.

DNA nanoflowers (DNFs) are an interesting example of inorganic hybrid materials that combine DNA and Mg_2_P_2_O_7_ in hierarchically structured materials with flower shaped morphology. DNFs are prepared enzymatically by rolling circle amplification (RCA) in which a DNA polymerase repeatedly traverses a circular DNA template synthesizing a long strand of DNA which then self-assembles (10,11). Incorporation of nucleotides during RCA releases pyrophosphate, which co-crystallizes with both the growing DNA and Mg^2+^ ions present in the amplification buffer (Figure 1) (12). A similar phenomenon also occurs during rolling circle transcription which is analogous to RCA, giving similar materials termed RNA microsponges (MSs) (13). In principle, DNFs and RNA MSs combine the structure and stability of Mg_2_P_2_O_7_ with the biological and programmable advantages of DNA or RNA. These have already shown potential in a number of applications, including the targeted delivery of chemotherapeutics by encoding aptamers into the DNA product (10,11), the delivery of miRNA for gene inhibition (13,14), cellular delivery of proteins (15), the delivery of DNAzymes (16), as immunostimulators (17), and in the immobilization and catalytic enhancement of enzymes (18).

Research aimed at tailoring the functions of these hybrids has focused on the organic components and includes: encoding functionality through the template (19), incorporating dye modified triphosphates (11,20), adding biologically relevant proteins (15,18), hybridization with dye-functionalized nucleic acids (14), condensing the structures with polycations (20) and intercalating drugs such as doxorubicin (10). We envisaged that by altering the inorganic component it would be possible to harness additional functionalities. Metal phosphates and pyrophosphates have been used in catalysis (21–24), electrochemical detection (25), as electrodes (26), and in supercapacitors (27). They also have multiple potential biomedical applications in therapeutics (28), as artificial bone and tooth scaffolds (29), as MRI contrast agents (30), and as photoluminescent probes (31). Altering the metal ion component of the pyrophosphate salts could therefore potentially expand the versatility and applicability of DNFs. Indeed, a similar strategy was very recently reported for preparing manganese and cobalt DNFs (32). Herein, we expand on the published study by identifying and evaluating variables to manipulate size, alter the morphology, and tailor the DNA content of the particles. We show for the first time that it is possible to prepare zinc DNFs and that DNF preparation is not limited to Ф29 DNA polymerase. We have also investigated the surface potential and hybridization properties of Mn, Co, Zn, and Mg particles, and demonstrate their stability towards enzymatic degradation. In addition, we describe, for the first time, the magnetic properties and structure of one such bio-inorganic hybrid, and show that it is possible to manipulate such materials with an external magnetic field.

## MATERIALS AND METHODS

General methods, protocols for the synthesis of oligonucleotides and cyclization of templates, details for the preparation of each type of DNF described in the manuscript, and protocols for analytical techniques are given in the Supplementary Information. Sequences of oligonucleotides used in this study are given in Table 1.

**Table 1.** Sequences of oligonucleotides used in this study. HPLC traces and mass spectra for oligonucleotides used in RCA are shown in the Supplementary Information. P represents a 5′ phosphate

Name	Sequence
Template 1 (T1)	5′-PTATAGCCCATGTGCTGCTGCTGCAGCGATACGCGTATCGCTATGGCATATCGTACGATATGCCGCAGCAGCATTACCGTCGTT-3′
Template 2 (T2)	5′-PATAGTGAGTCGTATTAGCTCGAGCTCGAGCAGCCGCGCCCTACCCTATCCCTCCCCTCGCGGCTGCTCGAGCTCGAGCATCCCT-3′
Splint 1 (S1)	5′-GCACATGGGCTATAAACGACGGTAA-3′
Splint 2 (S2)	5′-TAATACGACTCACTATAGGGAT-3′
Match probe	5′-TTACCGTCGTTTATAGC-Cy3-3′
Mismatch probe	5′-GTTGTCACTTAGTCCTA-Cy3-3′

### Investigating conditions for DNF formation

Before starting the experiment, 200 mM buffer stock solutions were prepared for the following: Tris pH 7.0, Tris pH 7.5, Tris pH 8.0, Tris pH 8.5, Tris pH 9.0, PIPES pH 7.5, HEPES pH 7.0, HEPES pH 7.5. 66.6 mM, 50 mM, 40 mM, 33.3 mM and 16.7 mM stock solutions of MnCl_2_, CoCl_2_ and ZnCl_2_ in MilliQ water.

#### Bst 2.0 DNA polymerase

The buffer stock solution (2 μl) and the divalent cation stock solution (6 μl) were added to a 200 μl PCR tube. An enzyme stock solution composed of T4 ligase cyclized T1 (0.5 μM), S1 (1 μM), dNTPs (3.33 mM), ammonium sulphate (16.7 mM), KCl (83.3 mM), 0.17% Tween 20 and Bst 2.0 (0.27 U/μl) in water was then prepared, adding the enzyme last. The solution was quickly vortexed and 12 μl was rapidly added to each PCR tube. The tubes were mixed by vortexing and incubated at 61.5°C for 14 h followed by 85°C for 20 min before rapidly cooling to 4°C. The tubes were centrifuged and the supernatant analysed by 0.8% agarose gel electrophoresis. Any precipitates that formed were washed 3 times with H_2_O, suspended in 20 μl of H_2_O and 7.5 μl of the samples were analysed by 0.8% agarose gel electrophoresis. The samples were then imaged by SEM if DNA was detected.

#### Ф29 DNA polymerase

The buffer stock solution (5 μl) and the divalent cation stock solution (6 μl) were added to a 200 μl PCR tube. An enzyme stock solution composed of T4 ligase cyclized T1 (0.67 μM), S1 (1.33 μM), dNTPs (4.44 mM), ammonium sulphate (22.2 mM), DTT (8.89 mM), and Ф29 (0.57 U/μl) in water was then prepared, adding the enzyme last. It was necessary to omit the DTT for the CoCl_2_ and ZnCl_2_ experiments. The solution was quickly vortexed and 9 μl was rapidly added to each PCR tube. The tubes were mixed by vortexing and incubated at 30°C for 20 h followed by 65°C for 10 min before rapidly cooling to 4°C. The tubes were centrifuged and the supernatant analysed by agarose gel electrophoresis. Any precipitates that formed were washed 3 times with H_2_O, and suspended in 20 μl of H_2_O. 7.5 μl of the samples were analysed by 0.8% agarose gel electrophoresis. The samples were then imaged by SEM if DNA was detected.

## RESULTS AND DISCUSSION

### Manganese DNA nanoflowers

Divalent manganese has similar chemical and biochemical behaviour to magnesium, and many DNA polymerases, including Bst 2.0 DNA polymerase (Bst) at 65°C or Ф29 DNA polymerase (Ф29), remain functional when Mg^2+^ cations in their active sites are replaced with Mn^2+^ (33,34). Unlike Mg^2+^, Mn^2+^ has five unpaired d electrons giving rise to magnetic properties. Consequently, we anticipated preparing manganese DNFs (MnDNFs) that could be manipulated by a magnetic field whilst still maintaining the desirable features of their magnesium counterparts (MgDNFs), such as large surface area, nanoscale dimensions, and enzymatic stability.

**Figure 2.**
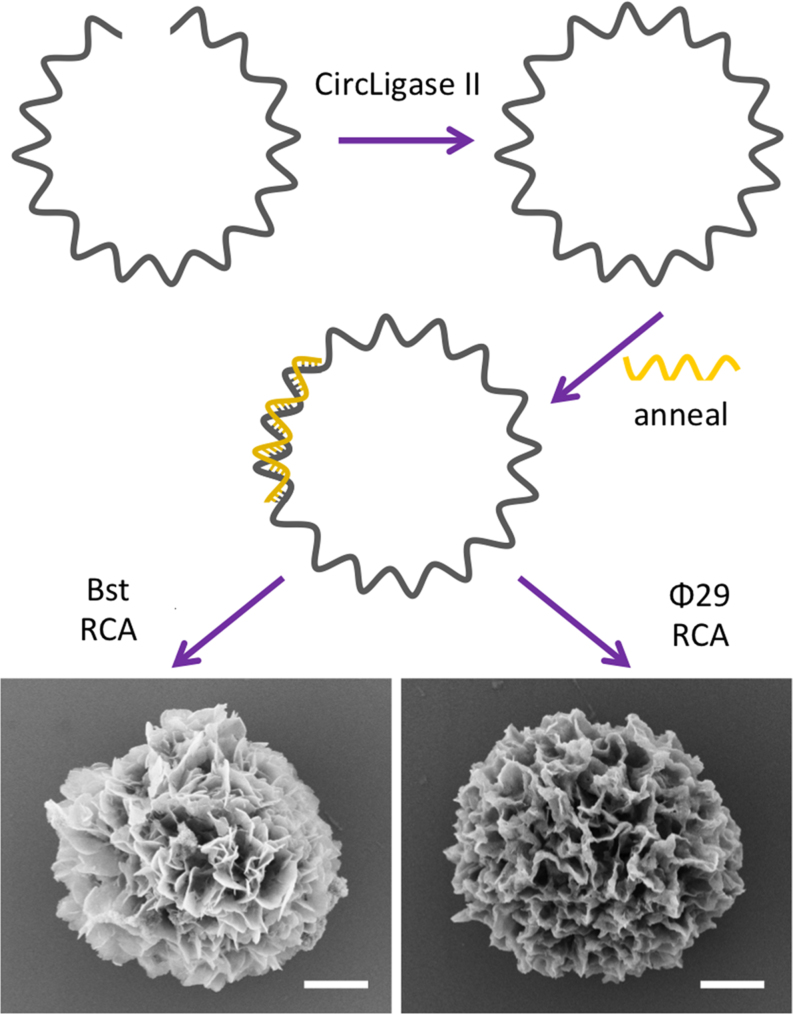
Initial method for the synthesis of MnDNFs. Scale bar represents 1 μm.

To test this, we replaced all Mg^2+^ sources present during MgDNF preparation with Mn^2+^ via MnCl_2_ and investigated using both Bst and Ф29. Despite being reported to catalyse RCA with reasonable DNA yield (35), Bst has not previously been used in the preparation of any DNFs, and we first determined whether Bst could be used to produce MgDNFs. We found that it could, providing the buffer has a final Mg^2+^ concentration of 15 mM or greater (SEM images are given in the SI, Figure S1).The published approach for preparing MgDNFs uses a cyclic DNA template made by T4 ligase-mediated circularization of a linear DNA strand in the RCA step without its isolation from the ligation mixture. As such, all components present in the buffer including Mg^2+^ are carried forward (10,19). T4 ligase, which requires Mg^2+^, was substituted with CircLigase II which uses Mn^2+^ as its cofactor. After addition of a primer to the CircLigase II ligation mixture, the sample was used in RCA reactions catalysed by Bst at 65°C or Ф29 at 30°C (Figure 2). In the amplification step we adopted the recommended buffer compositions, but replaced the Mg^2+^ sources with MnCl_2_. The final amplification buffer for the Bst catalyzed RCA contained 3.25 mM MnCl_2_ and had a mixture of Tris buffer (pH 8.8) and Tris-acetate buffer (pH 7.5) as a carryover from the ligation step. Similarly, the Ф29 buffer contained 11.25 mM MnCl_2_ and was a mixture of the CircLigase II Tris-acetate buffer (pH 7.5) from the ligation step and Tris buffer (pH 7.5).

Fortunately both polymerases produced a precipitate, and scanning electron microscopy (SEM) analysis revealed that these materials had hierarchical morphology (Figure 2). There was a clear difference between the two samples; Bst produced discrete particles with a size range of 3.6 ± 1.2 μm (number of particles counted (*n*) = 90; Supplementary Figure S2) whereas the Ф29 RCA product appeared softer with thick sheets and a broad size range (Supplementary Figure S3). In addition, agarose gel analysis indicated that the precipitate prepared using Ф29 incorporated DNA whereas those prepared using Bst did not (Supplementary Figure S4). Therefore, we suspected that the different morphologies are linked to the presence or absence of DNA.

#### Variables for controlling morphology and DNA levels within the DNFs

Three main differences were identified between the Bst and Ф29 RCA reactions: Mn^2+^ concentration, pH and temperature. We hypothesized that one or a combination of these factors controlled DNA levels within MnDNFs, and screened different RCA conditions, aiming to identify variables that control both DNA levels and particle size distribution. Due to the cost associated with CircLigase II, T4 ligase was used to cyclize the DNA template for subsequent experiments. In addition, purified cyclic templates were used in these experiments, removing any potential batch-to-batch variability associated with the efficiency of enzymatic ligation, and the cyclic product was desalted thoroughly before use, removing any Mg^2+^ remaining from the ligation buffer. Both Bst and Ф29 were investigated in the pH range of 7.0–9.0 with Mn^2+^ concentrations between 5 and 15 mM (Full details are given in the SI). After RCA, the reaction tubes were centrifuged and the supernatants were analysed to evaluate RCA efficiency (Supplementary Figures S6 and S8). Precipitates were washed with water, agarose gel electrophoresis was used to identify the presence of DNA (Supplementary Figures S5 and S7) and selected products were imaged by SEM. For direct comparison between MnDNFs and MgDNFs, the experiments were repeated using MgSO_4_ (Supplementary Figures S9–S12).

Gel-electrophoresis analysis of the supernatants revealed that long extended DNA strands were produced by both polymerases during Mn^2+^ RCA, with Bst becoming more processive with increasing pH whereas the efficiency of Ф29 was reduced at the higher pH. Interestingly, although the main DNA component present in the particles was too long to migrate out of the well of the gel, the Ф29 precipitates also contained a second band between the 10 kb marker and the well of the gel. This band was also present in the supernatant. This agrees with previous reports and is likely to be a double stranded amplification product (36). This band was not observed during Bst catalysed RCA.

**Figure 3.**
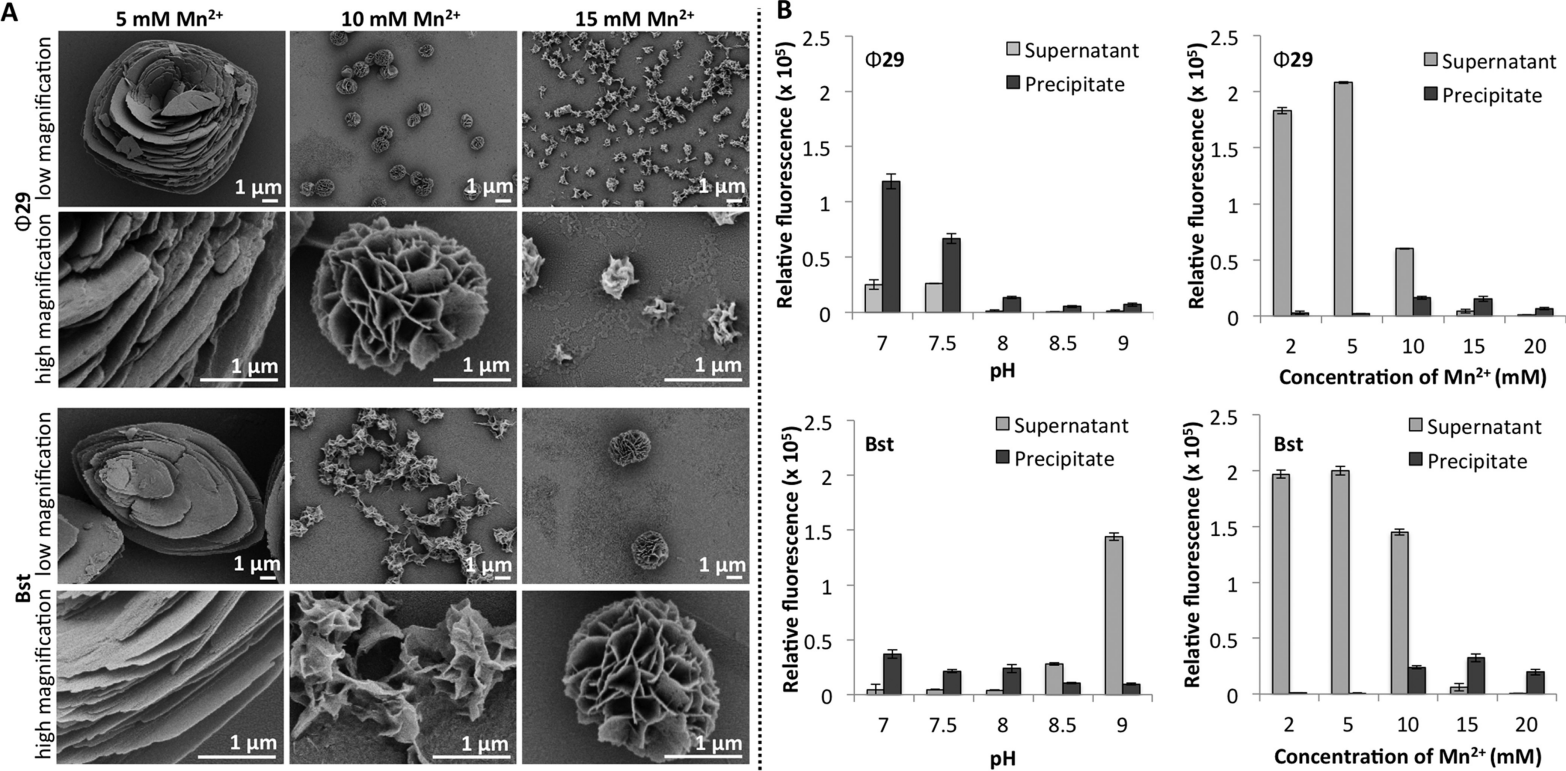
(**A**) SEM images illustrating the effect of MnCl_2_ concentration and choice of polymerase on the morphology and size of particles formed during RCA. The Bst RCA incubation time was 14 h at pH 7.5 and the Ф29 incubation time was 20 h at pH 8.0. (**B**) Fluorescence-based analysis comparing the levels of free DNA and DNA incorporated into the nanoconstructs as a function of enzyme, pH, and cation concentration. Samples were broken down using TBE to release DNA prior to incubation with SYBR gold. A plate reader was then used to compare the DNA levels. The effect of pH on Ф29 RCA was compared at 15 mM MnCl_2_ and the effect of concentration was evaluated at pH 8.0. The effect of pH on Bst was determined at 15 mM MnCl_2_ and the effect of concentration was evaluated at pH 7.0.

SEM imaging revealed that Mn^2+^ concentration has a striking effect on particle morphology (Figure 3A). At 5 mM, Bst-mediated RCA produced large shell-like particles with a layered structure and average length of 16 μm. At 10 mM, smaller particles of ∼1.5 μm in size begin to form in a mesh, and sonication yielded individual small NF particles. At 15 mM, MnDNFs with morphology similar to the reported MgDNFs were formed (10,11,15). These had similar size (∼2.5 μm) and morphology to the precipitate formed in the earlier experiments using Bst with the CircLigase II cyclized template, albeit with a narrower distribution (Table 2). Increasing the Mn^2+^ concentration further resulted in a large mesh formed of sheets (Supplementary Figure S13). A similar trend in morphology was observed for the material produced by Ф29. At 5 mM large particles formed with a layered structure but more square than those produced by Bst, the average longest length of these particles was 9 μm. At 10 mM Mn^2+^, Ф29 produced particles with an average diameter ∼1.3 μm which resemble the particles produced by Bst at 15 mM Mn^2+^ and the previously reported MgDNFs (10,11,15). Increasing the concentration of Mn^2+^ further resulted in much smaller particles ∼300 nm in diameter. This pattern in morphology was not observed when using Mg^2+^; at concentrations below 10 mM, no DNA was detected in any precipitates that formed and at the higher Mg^2+^ concentration (15 mM) nanoflower structures still formed. This was the case for both polymerases. We also observed that Tris buffer gave particles with narrower size distribution than HEPES buffer (Supplementary Figures S14–S16) and that the concentration of ammonium sulphate did not alter the morphology (Supplementary Figure S17).

**Table 2.** Average size of particles formed as a function of Mn^2+^ concentration. The Bst RCA reaction was incubated for 14 h at pH 7.0 whereas the Ф29 pH incubation time was 20 h and the pH was 8.0. Sizes refer to the longest dimension and *n* refers to the number of particles measured. Analysis was performed using ImageJ. See Supplementary Figures S18–S23 for SEM images used in the analysis

Enzyme	Concentration (mM)	Size range (μm)	*n*
Bst	5	15.8 ± 2.7	60
	10	1.4 ± 0.5	26
	15	2.6 ± 0.3	93
Ф29	5	8.8 ± 1.2	20
	10	1.3 ± 0.2	72
	15	0.312 ± 0.006	111

To further investigate the effect of pH and Mn^2+^ concentration of the DNA levels within the particles we used SYBR gold (a fluorescent dye that exhibits high fluorescence enhancement on binding to both single and double stranded DNA) to compare the relative quantities of DNA present in both the supernatants and the precipitates from a fixed RCA volume (Figure 3B). Samples were incubated in Tris-borate-EDTA (TBE) buffer overnight to sequester any Mn^2+^ cations and breakdown any precipitates prior to incubation with the dye and measurement. Using the earlier electrophoresis experiments as a guide, we used a 15 mM concentration of MnCl_2_ to evaluate the effect of pH. We identified that the pH at which the RCA reaction was conducted strongly affects the DNA levels within the particles and, whilst precipitation was observed for all pH’s when 15 mM Mn^2+^ was used in the reaction, pH 7.0 resulted in the highest levels of precipitated DNA for both polymerases. Next, we compared the effect of Mn^2+^ concentration; pH 8.0 was chosen for the Ф29 experiments and pH 7.0 for the Bst experiments. These conditions were chosen based on the earlier gel-electrophoresis results. Here, we observed that both polymerases produced the greatest amount of DNA at the lower Mn^2+^ concentrations (2–5 mM), and that this was greatly reduced with increasing levels of Mn^2+^. This is presumably due to the DNA product being trapped within the precipitates, preventing further extension. The same pattern was seen with SYBR green I and SYBR green II (Supplementary Figures S24 and S25), these dyes were used in addition to the universal nucleic acid stain SYBR gold (37) as they display different sensitivities towards nucleic acids; SYBR green I is known to bind double stranded DNA selectively (38,39), and SYBR green II is more sensitive towards single stranded DNA and RNA (40). Interestingly, when compared with Mg^2+^ (Supplementary Figure S26) higher DNA levels were found for Bst with Mn^2+^, but Mg^2+^ gave higher DNA levels with Ф29.

As mentioned above, this study demonstrates that Ф29 can be replaced with Bst in the preparation of MgDNFs providing that the buffer is supplemented with additional Mg^2+^ and, whilst the morphology remains similar, that the DNA component of the Bst MgDNFs is of shorter length. As with the MnDNFs a second band between the 10 kb marker and the well of the gel was observed for the DNFs produced using Ф29 but not when using Bst, suggesting that Bst does not produce a significant amount of double stranded DNA during RCA (36).

### Cobalt DNA nanoflowers

**Figure 4.**
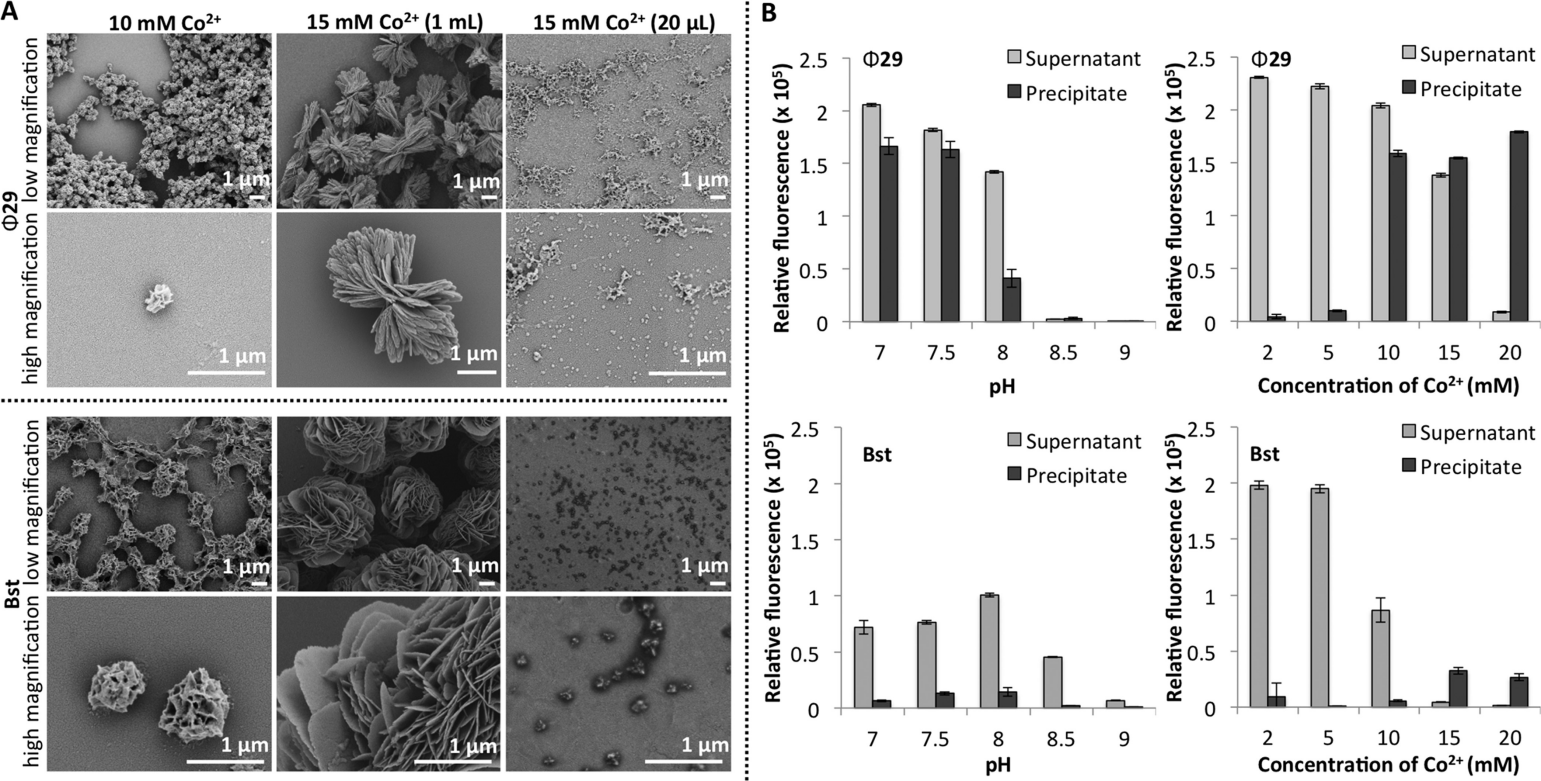
A) SEM images illustrating the effect of CoCl_2_ concentration and reaction scale on the morphology and size of particles formed during RCA at pH 7.0. The Bst RCA incubation time was 14 h whereas the Ф29 incubation time was 20 h. B) Fluorescence-based analysis comparing the levels of free DNA and DNA incorporated into the nanoconstructs as a function of enzyme, pH, and cation concentration. Samples were broken down using TBE to release DNA prior to incubation with SYBR gold. A plate reader was then used to compare the DNA levels. The effect of pH on Ф29 and Bst RCA was determined at 10 mM CoCl_2_ and the effect of concentration was evaluated at pH 7.0.

Next we confirmed that Co^2+^-catalysed RCA also produced DNFs, proving that divalent cations other than Mg^2+^ and Mn^2+^ can be used. Co^2+^ was chosen as it also has unpaired d electrons, so the nanoconstructs should possess magnetic properties, and most DNA polymerases can utilize Co^2+^ as a cofactor (33). Dithiothreitol (DTT) present in the buffer was found to be incompatible with CoCl_2_, resulting in a dark precipitate forming instantly on addition of the DTT. In contrast with previous literature, (32) we found it necessary to remove DTT when preparing CoDNFs. We screened different RCA conditions using the approach described above and identified those where nanomaterials were produced (Supplementary Figures S27-S30). Precipitates that were identified as containing DNA by gel electrophoresis were imaged by SEM (Figure 4A and Table 3). We also evaluated DNA levels using the SYBR dyes (Figure 4B, Supplementary Figures S37-S38). DNA was most efficiently incorporated into the nanostructures at pH 7–7.5 for Ф29 and pH 7–8 for Bst. As with Mn^2+^catalysed RCA, more free DNA is produced at lower metal cation concentrations and more DNA is captured in the precipitates at the higher concentrations (Figure 4B). Interestingly, if the total DNA in the precipitate is combined with the total free DNA our study shows that Ф29 with Co^2+^ produces more DNA than with Mn^2+^ or Mg^2+^. It also indicates that Ф29 has a greater tolerance to higher concentrations of Co^2+^ than Bst, and this will be the subject of further investigation. Of note, unlike the Mn^2+^ RCA reactions, the RCA reaction volume greatly altered the morphology of the materials that formed (Figure 4A). The reason for this is not known, and could be linked to the high levels of DNA and precipitate settling.

**Table 3.** Average size and standard deviation of particles formed during Co^2+^ RCA at pH 7.0. The Bst RCA incubation time was 14 h whereas the Ф29 incubation time was 20 h. Sizes refer to the longest dimension and *n* refers to the number of particles measured. Analysis was performed using ImageJ. See Supplementary Figures S31-S36 for SEM images used in the analysis

Enzyme	Scale (μl)	Concentration (mM)	Size range (μm)	*n*
Bst	100	10	0.79 ± 0.13	52
	20	15	0.210 ± 0.043	26
	1000	15	6.1 ± 0.7	26
Ф29	100	10	0.446 ± 65	108
	20	15	0.038 ± 0.008	176
	1000	15	3.8 ± 0.4	31

### Zinc DNA nanoflowers

**Figure 5.**
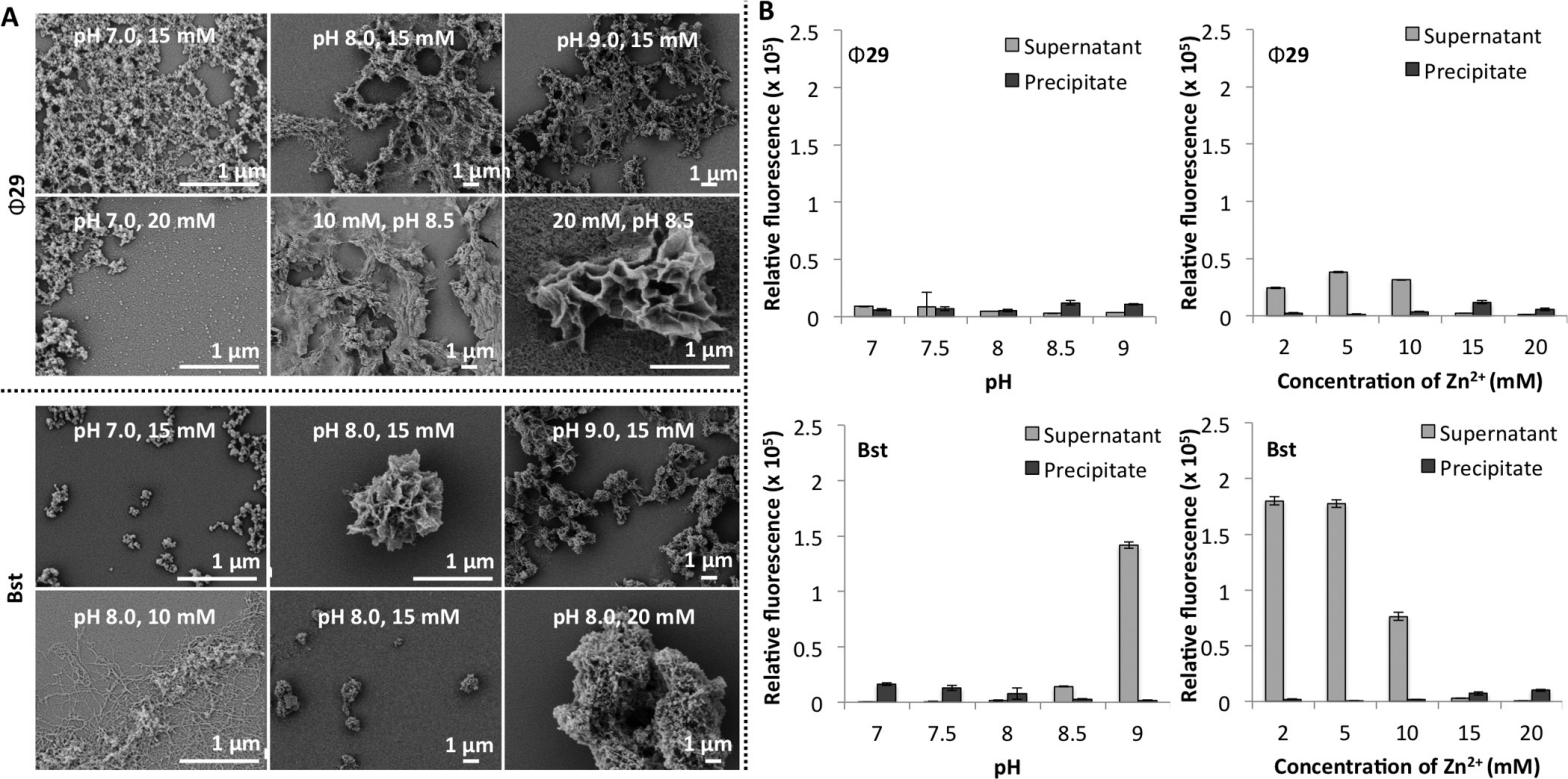
(**A**) SEM images illustrating the effect of ZnCl_2_ concentration and pH on the morphology and size of particles formed during RCA. The Bst RCA incubation time was 14 h whereas the Ф29 incubation time was 20 h. (**B**) Fluorescence-based analysis comparing the levels of free DNA and DNA incorporated into the nanoconstructs as a function of enzyme, pH, and cation concentration. Samples were broken down using TBE to release DNA prior to incubation with SYBR gold. A plate reader was then used to compare the DNA levels. The effect of pH on Ф29 RCA was determined at 15 mM ZnCl_2_ and the effect of concentration was evaluated at pH 8.5. The effect of pH on Bst was determined at 15 mM ZnCl_2_ and the effect of concentration was evaluated at pH 8.0.

We also evaluated Zn^2+^ as the cofactor in the preparation of DNFs as Bst has been reported to tolerate Zn^2+^ (A.K. Vashishtha, J. Wang, and W.H. Konigsberg, unpublished results) (33). Surprisingly, both polymerases produced DNA at the lower Zn^2+^ concentrations, with Bst showing greater DNA yields (Figure 5B). As with Mn^2+^, Mg^2+^ and Co^2+^ greater DNA levels in the precipitates were observed at higher cation concentrations. SEM analysis revealed that whilst DNA was observed in the precipitates produced at all pHs tested, only the RCA reactions at pH 7.0 produced discrete particles (Figure 5A). Unlike MnDNFs, MgDNFs and CoDNFs these ZnDNFs are much smaller with spherical morphology and it is possible these are amorphous particles formed of Zn_2_P_2_O_7_ and DNA explaining their smaller size and shape.

Finally, to determine if the trends in morphology are dependent on the DNA sequence, a second template and splint pair was prepared with a different nucleobase sequence and base composition (Table 1). These were then used in RCA reactions with several of the conditions described earlier in the text. The particles that formed showed a similar trend in morphology and size to those observed with the first template, representative images are shown in the SI (Supplementary Figures S39–S43). In brief, the MnDNFs that formed at 5 mM Mn^2+^ were large and layered, and increasing the concentration of Mn^2+^ resulted in the formation of the spherical flower morphology. The CoDNFs also formed petal like structures at 10 mM Co^2+^ and these were smaller at 15 mM. These results suggest that the morphology depends on the cation concentration to a greater extent than the nucleobase sequence.

### Characterization and properties of the nanoflowers

#### Elemental composition

**Figure 6.**
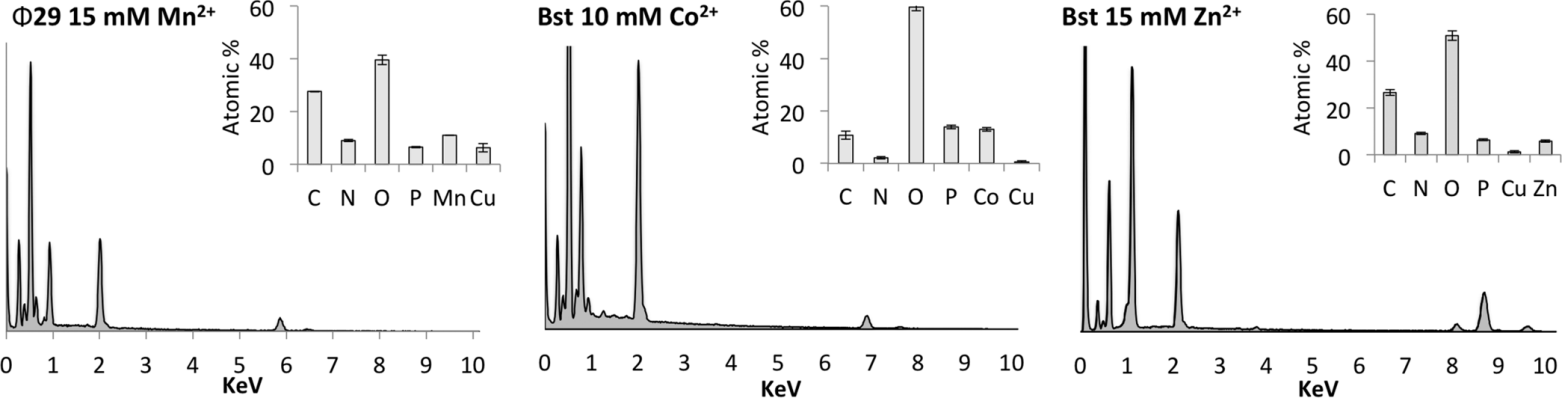
Exemplar SEM-based EDX characterization of the elemental compositions of the DNFs. EDX data is shown for MnDNFs prepared using Ф29 in Tris buffer pH 8.0, CoDNFs prepared in Tris buffer pH 7.0 and ZnDNFs prepared in Tris buffer pH 7.0. The CoDNFs prepared using a 1 ml RCA volume. Samples were analysed on a carbon support film with a copper mesh to minimize signals from mounting, which is why high carbon levels and copper are detected. See SI Figure S39 for other samples.

Energy-dispersive X-ray spectroscopy (EDX) was used to analyse the elemental composition of the MnDNFs, CoDNFs, and ZnDNFs (Figure 6 and Supplementary Figure S44). This confirmed that the MnDNFs were composed of Mn, P, O, N and C. The CoDNFs were formed of Co, P, O, N and C and the ZnDNFs were composed of Zn, P, O, N and C. We also carried out elemental analysis at different Mn^2+^ concentrations and the data are given in the SI (Supplementary Figures S45–S48). In agreement with the SYBR gold assay, increasing the concentration of Mn^2+^ during RCA resulted in precipitates with higher nitrogen content, presumably from the DNA (Supplementary Figure S48). Sulfur was not identified in the MnDNFs formed using HEPES buffer (Supplementary Figures S45–S47), providing evidence that buffer is not incorporated into the nanostructures.

#### Fourier-transform infrared spectroscopy (FTIR)

We used FTIR to support our theory that M_2_P_2_O_7_ (where M is a divalent metal cation) form the core of the particles, rather than alternative phosphate derivatives such as M_3_(PO_4_)_2_. The MnDNFs, CoDNFs and ZnDNFs all showed an asymmetric POP bridge stretch (ν^as^ POP) at around 900 cm^−1^ and a symmetric POP bridge (ν^s^ POP) stretch at 730 cm^−1^. The asymmetric vibration modes of the PO_3_ (ν^as^ PO_3_) gave a strong peak in the region of 1090–1070 cm^−1^ and the DNFs also show several overlapping broad bands in the region that corresponds to the stretching vibration of water (2800–3800 cm^−1^) with multiple bending vibrations of H_2_O (δ H_2_O) around 1640 cm^−1^. This suggests that the water molecules associated with the samples are in different environments. Additionally, the infrared (IR) spectrum of the MnDNFs (Supplementary Figure S50) was similar to that of previously published Mn_2_P_2_O_7_.2H_2_O (41). We then compared the IR spectra with Mn^2+^ and Co^2+^ phosphate and pyrophosphate salts, which were prepared by mixing corresponding metal dichloride with buffered pyrophosphate or phosphate based on a literature procedure for the synthesis of hydrated manganese(II) phosphate (Mn_3_(PO_4_)_2_·3H_2_O) (22). In all cases, the IR spectra matched with the pyrophosphate salts suggesting that the DNFs are composed of a derivative of M^2+^ with P_2_O_7_^4−^, most likely M_2_P_2_O_7_ rather than phosphate salts (Supplementary Figures S50–S58).

#### Surface potential

The surface potentials of the particles prepared under different conditions were compared using a Malvern Zetasizer Ultra instrument (Supplementary Tables S2 and S3). Particles were suspended in 10 mM aqueous NaCl. Due to the limitation of the technique, high quality data could not be obtained for some of the larger particles which have a tendency to settle. As expected, there was a significant degree of correlation between DNA incorporated into the MnDNFs and the magnitude of zeta potential which ranged from: –41.6 mV to –2.5 mV (most to least DNA) (Figure 3, Supplementary Tables S2 and S3). This suggests that the presence of DNA increases the surface charge and stability of the materials. The correlation for the CoDNFs and ZnDNFs, however, was less pronounced. As the surface potential of the particles can be controlled using the methods described in this paper these results will be of importance to other researches developing similar nanostructures for therapeutic applications such as MRI contrast agents or as targeted delivery agents.

#### Serum stability of DNFs

We next confirmed that the DNA within DNFs retained the high serum stability previously observed for MgDNFs (15). ZnDNFs, CoDNFs and MnDNFs were incubated in cell culture medium supplemented with 10% fetal bovine serum (FBS) and the DNA content was analysed at various time points. FBS was chosen to simulate the extracellular conditions to which a therapeutic oligonucleotide would be exposed and contains predominantly endonucleases. The stability of the DNA in the particles was determined using agarose gel electrophoresis and compared with the stability of free λ-DNA incubated under the same conditions. In all cases, the original morphology of the particles remained intact, even after 48 h (Supplementary Figures S60–S67). Whilst the DNA component of all DNFs proved more stable than the λ-DNA control (Supplementary Figure S59), our data indicates that the smaller particles are more stable to digestion than the larger micron-sized particles. The results also suggest that the CoDNFs are more stable than the MnDNFs. We noticed that after treatment with 10% FBS the particles were less aggregated, one possible explanation for this is that the individual particles may be joined to each other by long DNA strands, and that the naked DNA between the particles is digested.

#### Magnetic properties of MnDNFs and CoDNFs

**Figure 7.**
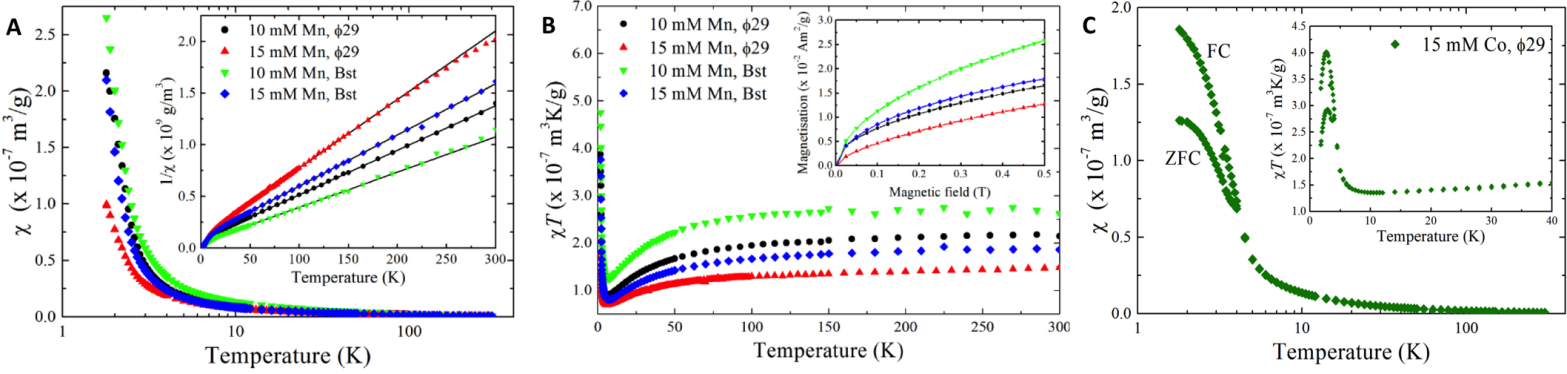
(**A**) Field-cooled powder magnetic susceptibility χ as a function of temperature of four MnDNF samples taken in an applied dc magnetic field of μ_0_*H* = 0.025 T. The inset shows the inverse susceptibility data (symbols) and the associated fits of χ(*T*) to the Curie-Weiss law (lines). (**B**) The same data plotted as χ*T*. The inset shows the magnetization measured at *T* = 1.8 K in the same samples. (**C**) Zero-field-cooled (ZFC) and field-cooled (FC) powder magnetic susceptibility χ as a function of temperature of a CoDNF sample taken in an applied dc magnetic field of μ_0_*H* = 0.025 T. The inset shows the same data plotted as χ*T* in the low-temperature region. CoDNFs prepared using a 1 ml RCA volume with 15 mM CoCl_2_ were used.

We have also investigated the magnetic properties of the MnDNFs and CoDNFs. Previous studies have used isothermal methods to prepare nucleic acid materials that can be manipulated using a magnet but these used folate-labelled primer strands to allow immobilization on streptavidin coated magnetic beads (42,43). Interestingly, the study using RCT shows a TEM image with MgDNF-like structures (43). It is possible that these materials are RNA NFs or MSs formed from magnesium pyrophosphate and RNA with the magnetic particles embedded in the structure, rather than RNA coated magnetic nanoparticles. Unlike the reported studies, the magnetic materials here are prepared during RCA, the constructs are considerably larger, and the approach does not require the use of streptavidin nanoparticles or modified nucleic acids during the preparation. After confirming that the MnDNFs and CoDNFs could be manipulated using a magnet and could be captured on a commercial magnetic rack (Supplementary Figure S68), the low-temperature magnetic properties of the materials were then studied in more detail. The magnetometry data collected on the MnDNFs are shown in Figure 7. While it is difficult to draw definitive conclusions from powder susceptibility in anisotropic systems, some information can be inferred from the data. The mass susceptibility of all MnDNF samples (Figure 7A, main panel) initially increases on cooling in a Curie-Weiss manner. The zero-field cooled (ZFC) and field cooled (FC) curves displayed no noticeable differences, and no clear evidence of magnetic ordering is present down to the lowest temperature measured. The product χ*T* of each material (Figure 7B, main panel) shows a steady value at high temperatures indicating that the compounds are in the paramagnetic phase where temperature fluctuations overcome internal interactions. Variations in χ*T* on cooling are indicative of departures from paramagnetic behaviour, and can be caused by interactions between the spins, or the thermal depopulation of crystal-field split magnetic energy levels. The MnDNFs all exhibit a drop in χ*T* as the temperature is cooled below 100–150 K. This is most likely caused by antiferromagnetic interactions between Mn^2+^ ions, as observed in several manganese pyrophosphates, including Mn_2_P_2_O_7_ (44–47). The magnetic susceptibility data above *T* = 50 K was fitted to a Curie-Weiss law and the fitted lines are plotted, along with the data displayed as 1/χ(*T*) in the inset to Figure 7a. The Curie-Weiss temperatures (Θ_CW_) determined from the fits are in the range –15 to –21 K and are tabulated in Table 4. This is similar to Θ_CW_ = –13 K found for Mn_2_P_2_O_7_ (44). However, while Mn_2_P_2_O_7_ orders antiferromagnetically at 14 K, none of our MnDNFs exhibit clear signs of long-range magnetic order. Moreover, as temperature is reduced below 7 K the χ*T* for all samples begins to increase. The temperatures of the minima in susceptibility *T*_min_ are recorded in Table 4. This increase in χ*T* points towards the presence of additional ferromagnetic interactions, or the onset of canted antiferromagnetism (also known as weak ferromagnetism), in which the non-colinear arrangement of antiferromagnetically aligned spins gives rise to a net ferromagnetic moment in a particular direction.

**Table 4.** Parameters derived from the magnetic susceptibility data. Θ_CW_ is the Curie–Weiss temperature resulting from fits of χ(*T*) to the Curie-Weiss law. *N* is an estimate of the number of spins per gram assuming a high-spin state (*S* = 5/2 for Mn and 3/2 for Co) and a *g*-factor of 2. *T*_min_ (*T*_max_) is the temperature of the minimum (maximum) observed in the χ*T* data

Sample	Θ_cw_ (K)	*N* (×10^21^)	*T*_min_ (K)	*T*_max_ (K)
Ф29 10 mM Mn^2+^	−19.3(2)	2.5	6.5(3)	-
Ф29 15 mM Mn^2+^	−16.4(1)	1.6	6.0(3)	-
Bst 10 mM Mn^2+^	−15.0(7)	3.2	6.8(1)	-
Bst 15 mM Mn^2+^	−21.0(3)	2.2	7.1(1)	-
Ф29 15 mM Co^2+^ (1 ml)	−22.2(5)	5.9	10.5(3)	2.7(1)

Also shown in Table 4 is an estimate of the number of Mn^2+^ spins per gram of sample deduced from the Curie-Weiss fit, assuming a high-spin *S* = 5/2 configuration and a *g*-factor of 2. The data show that a higher number of spins in the final product is obtained for the samples prepared using 10 mM MnCl_2_ in the reaction compared to the 15 mM reactions, and that the Bst polymerase yields more spins per mass than the Ф29. These numbers are in good agreement with the results of the EDX analysis shown in Figure 6 and Supplementary Figure S44.

The magnetization of the MnDNFs measured at 1.8 K (inset to Figure 7B) shows a gradual rise and no saturation up to 0.5 T. There is no hysteresis between up and down sweeps of the magnetic field.

The data for the CoDNFs (Figure 7C) show similarities with that of MnDNFs: it exhibits paramagnetic behaviour at high temperatures with a Θ_CW_ = –22.2(5) K; a decrease in χ*T* once the temperature is cooled below 150 K, followed by an increase below *T*_min_ = 10.5(1) K; and a slow non-hysteretic rise in magnetization up to 0.5 K (not shown). However, the CoDNF sample also exhibits a bifurcation of the ZFC and FC susceptibility curves below a temperature of 4 K (Figure 7C, main panel). In addition, χ*T* begins to decrease again below *T*_max_ = 2.7(1) K (Figure 7C, inset). This could indicate the presence of further antiferromagnetic interactions, a reconfiguration of canted spins, or the splitting of the Co^2+^ spin multiplet. We note that bulk Co_2_P_2_O_7_ displays antiferromagnetic order below 10.1 K (48), which is close to the value of *T*_min_ observed for our CoDNFs. The number of spins per gram shown in Table 4 was estimated assuming *S* = 3/2 and *g* = 2 for each Co^2+^ ion.

In summary, at high temperatures the magnetic data for the MnDNFs and CoDNFs are similar to that for bulk Mn_2_P_2_O_7_ (44) and Co_2_P_2_O_7_ (48), respectively. However, at lower temperatures additional energy scales are present in the DNFs that give rise to changes in χ*T* not observed in the bulk materials. Possible explanations for this observation include: (i) the spins in the DNFs are interacting strongly with or via their surroundings; (ii) although the individual DNFs investigated fall in the size range of 300 nm to 10 μm, the complex nanoscale structure of the flowers leads to non-bulk behaviour; (iii) disorder inherent in the bulk material that makes up the DNF is affecting the spin response; or (iv) the crystal structure of the transition-metal DNFs is not the same as the equivalent diphosphate materials. This is a subject of ongoing investigations.

#### Hybridization properties

**Figure 8.**
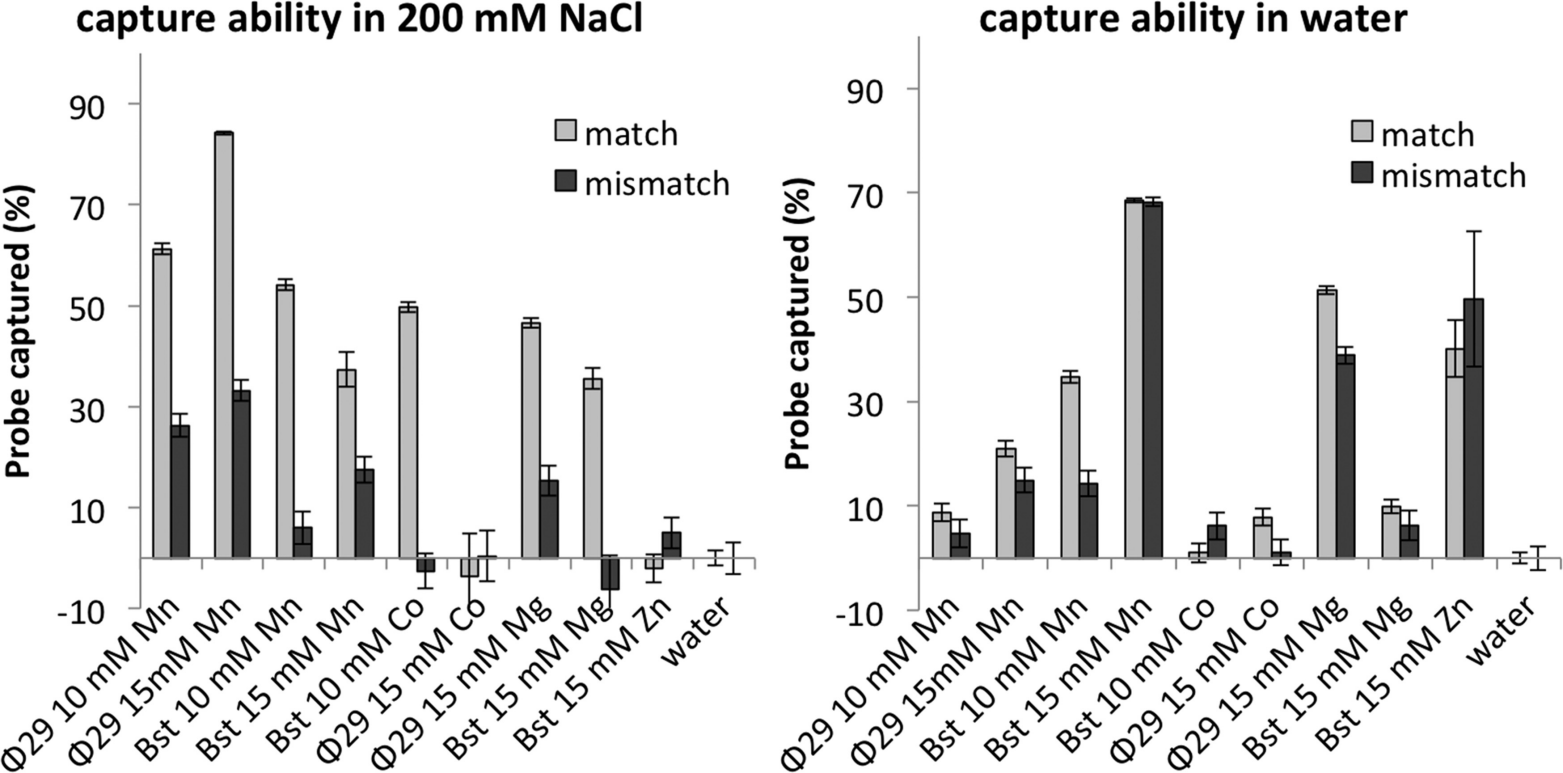
The ability of different DNFs to hybridize with and capture a complementary DNA strand. DNFs prepared from the equivalent of 20 μl RCA volume were incubated with 100 μl of either 50 nM Cy3 labelled complementary DNA probe (match) or 50 nM of a scrambled version (mismatch) at room temperature for 1 h in the presence of 200 mM NaCl (left) or water (right). The particles were collected by centrifugation and the percentage of probe left in solution was determined using a fluorescence plate reader. Greater selectivity was observed in saline than water, and overall particles prepared using Bst appear to show greater specificity than their Ф29 counterparts.

For the DNFs described here to find use in targeted delivery through aptamer incorporation or in bio-separation based applications it is imperative that the DNA component of the DNFs is available to interact with and bind to the intended target. To test this, we evaluated the ability of the different DNFs to selectively hybridize with a complementary oligonucleotide, and compared this to a scrambled base sequence control (Figure 8). The conditions of incubation greatly affected the ability of the DNFs to selectively hybridize and capture the matched sequence over the scrambled control. Incubation of the DNFs with the probes in 200 mM NaCl resulted in the greatest discrimination suggesting that DNFs could find application in magnetic assisted bio-separation or targeted delivery. Our current hypothesis for improved selectivity in saline over water is that NaCl screens the charges of the phosphate backbone in the probe preventing ionic interactions between the inorganic component and the probes, allowing specific base-pairing to dominate relative to non-specific charge-charge interactions. Whilst we expected pH buffering would improve the selectivity, in the majority of cases incubation in PBS resulted in reduced hybridization and it is feasible that PBS reduced hybridization through competitive interactions. Of particular note, the presence of divalent cations resulted in the capture of both the matched and mismatched probes (data not shown). We suspect that the divalent cations lead to non-selective probe capture either by forming salt bridges between the inorganic scaffold and the probes or by interactions through the nitrogen atoms in the DNA bases.

## CONCLUSIONS

We have developed methodology to produce nano- and microscale hybrid DNA-inorganic materials that can be prepared in a range of shapes, show enhanced stability against DNA degradation and can be manipulated using an external magnetic field. These particles can be prepared enzymatically using different divalent cations (Mn^2+^, Co^2+^ and Zn^2+^) which co-crystallize with DNA and pyrophosphate anions, constituting a novel class of materials. By simply altering the composition of the enzyme buffers we show that it is possible to alter the inorganic core of DNFs, control the morphology and size between 40 nm to 16 μm, manipulate the DNA levels within the particles, and alter their surface potential. By changing the polymerase enzyme it is also possible to control the morphology further and vary the length of DNA within the constructs. We have also investigated the magnetic properties of the materials providing insight into the structures of these materials. Finally, we have shown that under certain conditions these structures are capable of selectively binding to their complementary DNA target, and consequently the DNA is free to interact, rather than being tied up in the structures. The tuneable and selective properties of these materials suggests applications in drug delivery, sensing, biocatalysis, energy (as supercapacitors), and separation technologies.

## DATA AVAILABILITY

Compositions of enzyme buffers are available on supplier's websites (http://www.lucigen.com/phi29-DNA-Polymerase/ and https://www.neb.com/products/m0537-bst-20-dna-polymerase#Product%20Information_Properties%20and%20Usage). The magnetometry data presented in this paper will be made available at http://wrap.warwick.ac.uk/103500.

## Supplementary Material

Supplementary Data
