# Supplementary material for: Preparation and characterization of manganese, cobalt and zinc DNA nanoflowers with tuneable morphology, DNA content and size
Source: Nucleic Acids Res. 2018 Jul 12;46(15):7495–505. doi: 10.1093/nar/gky630 (PMC6125639; doi:10.1093/nar/gky630)
Supplement: Supplementary Data [file gky630_supplemental_figures.pdf]

Supplementary Material

Preparation and characterisation of manganese, cobalt and zinc DNA nanoflowers with tuneable morphology, DNA content and size

Ysobel R. Baker[1], Jinfeng Chen[1], Jason Brown[2], Afaf H. El-Sagheer[1,3], Philip Wiseman[1], Errin Johnson[4], Paul Goddard[5] and Tom Brown[1]*

[1] Department of Chemistry, University of Oxford, Oxford, Oxfordshire, OX1 3TA, UK
[2] Department of Physics, University of Oxford, Oxford, Oxfordshire, OX1 3PU, UK
[3] Chemistry Branch, Faculty of Petroleum and Mining Engineering, Suez University, Suez 43721, Egypt
[4] Sir William Dunn School of Pathology, University of Oxford, Oxford, Oxfordshire, OX1 3RE, UK
[5] Department of Physics, University of Warwick, Coventry, Warwickshire, CV4 7AL, UK

* To whom correspondence should be addressed. Tel: +44 1865 275413 Email: tom.brown@chem.ox.ac.uk

Contents

Experimental 2
- General techniques 2
- Polyacrylamide gel electrophoresis (PAGE) 2
- Agarose gel electrophoresis 3
- Scanning electron microscopy 3
- Energy dispersive X-ray spectroscopy 3
- Fourier-transform infrared spectroscopy (FTIR) 3
- Magnetometry 3
- Oligonucleotide synthesis 4
- CircLigase II cyclisation of T1 5
- T4 ligase cyclisation of T1 6
- T4 ligase cyclisation of T2 6
- Screening experiments 6
  - Bst 2.0 DNA polymerase screening 7
  - Φ29 DNA polymerase screening with $MnCl_2$ 7
  - Φ29 DNA polymerase screening with $CoCl_2$ and $ZnCl_2$ 7
- Comparison of DNA levels using SYBR Gold, SYBR Green I, and SYBR Green II 7
- Preparation of inorganic material for IR studies 8
- Preparation of particles for magnetic studies 8
- Enzymatic stability studies 10

Supplementary figures 12

Agarose gel analysis for $MnCl_2$ screening experiments 14
Supplementary MnDNF SEM images 18
SEM images showing the effect of buffer on particle morphology 18
SEM images used to determine particle size 23
SYBR green I and SYBR green II analysis of DNA levels in MnDNFs 26
SYBR gold analysis of DNA levels in MgDNFs 28
Agarose gel analysis for $CoCl_2$ screening experiments 29
Supplementary CoDNF SEM images 31
SYBR green I and SYBR green II analysis of DNA levels in CoDNFs 35
SEM images of particles formed using T2 and S2 37
Additional Energy-dispersive X-ray spectroscopy (EDX) data 40
Infrared spectra 44
Surface potential of various DNFs 49
MnDNF and CoDNF serum stability assays 51
Mass spectra of the DNA templates and splints used in this study 56
References 59

Experimental

All chemicals were purchased from Sigma-Aldrich unless otherwise specified and used without further purification. NxGen Phi29 DNA polymerase (Φ29) was purchased from Lucigen. CircLigase II ssDNA Ligase was purchased from Epicentre (Madison, WI, USA). T4 DNA ligase was purchased from Promega. Bst 2.0 DNA polymerase and deoxynucleotides (dNTPs) solution mix were purchased from New England Biolabs (UK). 1 kb DNA ladder was purchased from New England Biolabs (UK). Solid supports, standard DNA and RNA phosphoramidites, and reagents were purchased from Link Technologies, Glen Research and Applied Biosystems Ltd. NAP-25 and NAP-10 columns were purchased from G.E. Healthcare Life Sciences. Scanning electron microscopy (SEM) consumables were purchased from Agar scientific, and used without further treatment.

General techniques

Polyacrylamide gel electrophoresis (PAGE)

The DNA product was mixed with an equal volume of formamide and denatured by heating at 95 °C for 5 min. The mixture was cooled quickly on ice, and then loaded on an 8% denaturing PAGE gel (acrylamide:bisacrylamide 19:1, 40% acrylamide solution, 7 M urea, prepared in a 1X Tris-borate-EDTA (TBE) buffer). The gel was run in 1X TBE buffer at 20 W for 2.5 h at room temperature and

visualised using G:Box (Syngene). The cyclised template bands were excised, crushed and soaked in MilliQ water (10 mL) overnight at 37 °C with shaking. After filtration and evaporation of the MilliQ water, the cyclised templates were desalted using NAP-25 followed by NAP-10 columns. The DNA concentration of circularised templates were measured with Nanodrop 2000C spectrometer (Thermo Scientific).

Mass analysis

After purification, the molecular weight (MW) and analytical HPLC traces of oligonucleotides were recorded by mass spectrometry either using a Bruker micrOTOF™ II focus ESI-TOF MS instrument in ES^- mode or a XEVO G2-QTOF MS instrument in ES^- mode (Table S1) and quantified based upon their absorption at 260 nm using the nearest neighbour molar extinction coefficient.

Agarose gel electrophoresis

The products of RCA were visualised using a 0.8% (w/v) agarose gel cast with TBE buffer containing 0.25X SYBR™ Gold (ThermoFisher). 7.5 µL of sample was mixed with 2.5 µL of 5X GoTaq® Green buffer (Promega) before adding the sample to the well. The gels were run at room temperature (126 V) and imaged using a G:Box (Syngene).

Scanning electron microscopy

Scanning electron microscopy (SEM) analysis was performed using a Zeiss Sigma 300 Field Emission Gun Scanning Electron Microscope (FEG-SEM). 10 µL of the precipitate suspension in water was added to a silicon wafer chip and dried at 50 °C for 15 min. This was then coated with gold before imaging.

Energy dispersive X-ray spectroscopy

Energy dispersive X-ray spectroscopy (EDX) analysis was performed using a Quanta 600 FEG scanning electron microscope equipped with an Oxford Instruments INCA Energy detector. 2.5 µL of the precipitate suspension in water was dried on a carbon film on a 200 Mesh copper grid before imaging to reduce substrate interaction.

Fourier-transform infrared spectroscopy (FTIR)

Infrared spectra (IR) were recorded on a Bruker Tensor 27 FTIR spectrophotometer using the standard ATR cell.

Magnetometry

Direct-current magnetic susceptibility measurements were performed on powdered samples of 10 mM and 15 mM Mn^{2+} DNFs catalysed by Φ29 (masses of 0.8 mg and 0.65 mg, respectively), 10 mM and 15 mM Mn^{2+} DNFs catalysed by Bst 2.0 (0.2 mg and 1.0 mg, respectively), and 15mM Co^{2+} DNFs catalysed by Φ29 (0.6 mg). The samples were placed inside a gelatin capsule, which was fixed inside a plastic drinking straw and attached to the end of a brass rod. The sample's magnetisation (*M*) was

then measured in an applied field of $\mu_0 H$ = 0.025 T for temperatures in the range 1.8 ≤ T ≤ 300 K, with a Quantum Design SQUID magnetometer. In the linear limit, the magnetic susceptibility (χ) was deduced from this measurement using $\chi = M/H$. An isothermal measurement of the magnetisation at 2 K was also recorded for applied fields up to $\mu_0 H$ = 0.5 T.

Oligonucleotide synthesis

Solid supports, standard DNA and RNA phosphoramidites, and reagents were purchased from Link Technologies Ltd, Glen Research and Applied Biosystems Ltd. Single stranded oligonucleotides were synthesised using a standard 1.0 μmol phosphoramidite cycle of acid-catalysed detritylation, 35 s coupling, capping and iodine oxidation on an Applied Biosystems 394 automated DNA/RNA synthesiser. The 5' phosphate was added using a 2-[2-(4,4'-dimethoxytrityloxy)ethylsulfonyl]ethyl-(2-cyanoethyl)-(N,N-diisopropyl)-phosphoramidite. Stepwise coupling efficiencies and overall yields were determined by automated trityl cation conductivity monitoring facility and in all cases were more than 98%. The oligonucleotides were cleaved from the solid support and deprotected by treatment with concentrated aqueous ammonia solution at room temperature for 1 h followed by heating in a sealed glass vial at 55 °C for 5 h. The aqueous solution of ammonia was then removed by evaporation prior to oligonucleotide purification.

Linear oligonucleotides were purified by reverse-phase high performance liquid chromatography (RP-HPLC) on a Gilson system using a Luna 10 μm C8 100 Å pore Phenomenex 10 x 250 mm column with a gradient of acetonitrile in triethylammonium bicarbonate increasing from 100% buffer A to 50% buffer B over 20 min with a flow rate of 4 mL/min (buffer A: 0.1 M triethylammonium bicarbonate, pH 7.5, buffer B: 0.1 M triethylammonium bicarbonate, pH 7.5 with 50% acetonitrile). The elution of oligonucleotides was monitored by UV absorption at 298 nm. After HPLC purification, the molecular weight (MW) of oligonucleotides was recorded by mass spectrometry on either a Bruker micrOTOF™ II focus ESI-TOF MS instrument in ES^- mode or a XEVO G2-QTOF MS instrument in ES^- mode and quantified based upon their absorption at 260 nm using the nearest neighbour molar extinction coefficient. Analytical HPLC traces were recorded on either a Bruker micrOTOF™ II focus ESI-TOF MS instrument or a XEVO G2-QTOF MS.

Name	Sequence	Required mass	Found Mass
Template 1 (T1)	5′-PTATAGCCCATGTGCTGCTGCTGCAGCGATACGC GTATCGCTATGGCATATCGTACGATATGCCGCAGCAG CATTACCGTCGTT-3′	25597	25598
Template 2 (T2)	5′-PATAGTGAGTCGTATTAGCTCGAGCTCGAGCAGC CGCGCCCTACCCTATCCCTCCCCTCGCGGCTGCTCG AGCTCGAGCATCCCT-3′	25690	25692
Splint 1 (S1)	5′-GCACATGGGCTATAAACGACGGTAA-3′	7724	7723
Splint 2 (S2)	5′-TAATACGACTCACTATAGGGAT-3′	6742	6742
Match probe	5′-TTACCGTCGTTTATAGC-Cy3-3′	5658	5658
Mismatch probe	5′-GTTGTCACTTAGTCCTA-Cy3-3′	5658	5659

Table S1. Sequences of oligonucleotides used in this study. HPLC traces and mass spectra for oligonucleotides used in rolling circle amplification (RCA) are shown later (Fig S69-74). P represents a 5′ phosphate.

CircLigase II cyclisation of T1

T1 (50 pmol) was cyclised using CircLigase II according to the manufacturer's instructions. In short, T1 (50 pmol) and 5 µL CircLigase II ssDNA Ligase (5 U/µL) in 100 µL of 1X CircLigase II reaction buffer (33 mM Tris-acetate (pH 7.5), 2.5 mM $MnCl_2$, 66 mM potassium acetate, and 0.5 mM dithiothreitol (DTT)) was incubated at 60 °C for 16 h before heating to 80 °C for 10 min to denature the enzyme. This was used without purification.

Bst RCA using CircLigase II cyclised T1

S1 (60 pmol) in 10 µL of 10X $MnCl_2$ substituted Bst buffer (200 mM Tris-HCl, 100 mM $(NH_4)_2SO_4$, 20 mM $MnCl_2$, 500 mM KCl, 1% Tween® 20, pH 8.8 @ 25°C) was added to 50 µL of CircLigase II cyclised T1 mixture. This was heated to 95 °C for 5 min and cooled to 20 °C at a rate of 0.5 °C/min. 16 µL of H_2O, 20 µL of dNTPs mix (10 mM) and 4 µL of Bst 2.0 polymerase were added. The mixture was incubated at 65 °C for 14 h followed by 85 °C for 20 min before cooling to room temperature. The tubes were centrifuged and the precipitate that formed was washed 3 times with H_2O then suspended in 20 µL of H_2O. 7.5 µL of the samples were analysed by 0.8% agarose gel electrophoresis. The samples were also analysed by SEM.

Φ29 RCA using CircLigase II cyclised T1

S1 (60 pmol) in 10 µL of 10X $MnCl_2$ substituted Φ29 buffer (500 mM Tris-HCl, 100 mM $(NH_4)_2SO_4$, 40 mM DTT, 100 mM $MnCl_2$, pH 7.5 @ 25°C) was added to 50 µL of CircLigase II cyclised T1 mixture. This was heated to 95 °C for 5 min and cooled to 20 °C at a rate of 0.5 °C/min. 15 µL of H_2O, 20 µL of dNTPs mix (10 mM) and 5 µL of Φ29 polymerase were added. The mixture was incubated at 30 °C for 20 h followed by 65 °C for 10 min before cooling to room temperature. The tubes were centrifuged and the precipitate that formed was washed 3 times with H_2O then suspended in 20 µL of H_2O. 7.5 µL of the samples were analysed by 0.8% agarose gel electrophoresis. The samples were also analysed by SEM.

T4 ligase cyclisation of T1

T1 (4.8 nmol) and S1 (9.6 nmol) were annealed in 3.9 mL of 1X T4 ligase buffer (30 mM Tris-HCl, pH 7.8 at 25°C, 10 mM $MgCl_2$, 10 mM DTT, and 1 mM adenosine triphosphate (ATP)) by heating the sample to 95 °C for 5 min followed by cooling to room temperature at a rate of 0.5 °C/min. T4 DNA ligase (100 µL, 3U/µL) was then added and the sample incubated at 25 °C for 16 h before the ligation was stopped by heating to 95 °C for 5 min. The volume of the sample was reduced to approximately 1 mL using a CentriVap centrifugal concentrator at 55 °C and purified by 8% denaturing polyacrylamide gel electrophoresis (PAGE). The gel was visualised using a UV lamp and the circularised template bands were excised, crushed and soaked in water (10 mL) overnight at 37 °C. After filtration and evaporation of the water, the cyclised templates were desalted using two NAP-25 columns.

T4 ligase cyclisation of T2

T2 (6.0 nmol) and S2 (12 nmol) were annealed in 4.9 mL of 1X T4 ligase buffer (30 mM Tris-HCl, pH 7.8 at 25°C, 10 mM $MgCl_2$, 10 mM DTT, and 1 mM ATP) by heating the sample to 95 °C for 5 min followed by cooling to room temperature at a rate of 0.5 °C/min. T4 DNA ligase (125 µL, 3U/µL) was then added and the sample incubated at 25 °C overnight before the ligation was stopped by heating to 95 °C for 5 min. The volume of the sample was reduced to approximately 1 mL using a CentriVap centrifugal concentrator at 55 °C and purified by 8% denaturing polyacrylamide gel electrophoresis (PAGE). The gels were visualised using a UV lamp and the circularised template bands were excised, crushed and soaked in water (10 mL) overnight at 37 °C. After filtration and evaporation of the water, the cyclised templates were desalted using two NAP-25 columns.

Screening experiments

Before starting the experiment, 200 mM buffer stock solutions were prepared for the following: Tris pH 7.0, Tris pH 7.5, Tris pH 8.0, Tris pH 8.5, Tris pH 9.0, PIPES pH 7.5, HEPES pH 7.0, HEPES pH 7.5. 66.6 mM, 50 mM, 40 mM, 33.3 mM and 16.7 mM stock solutions of $MnCl_2$ in water were prepared by diluting a 1 M solution of $MnCl_2$ in water (purchased form Sigma Aldrich) with MilliQ water. The same concentration solutions were also prepared for $CoCl_2$ and $ZnCl_2$ by diluting a 1 M solution of either

$CoCl_2$ or $ZnCl_2$ which were freshly prepared before each experiment by dissolving $CoCl_2.6H_2O$ or $ZnCl_2$ in MilliQ water.

Bst 2.0 DNA polymerase screening

The buffer stock solution (2 µL) and the divalent cation stock solution (6 µL) were added to a 200 µL PCR tube. An enzyme stock solution composed of T4 ligase cyclised T1 (0.5 µM), S1 (1 µM), dNTPs (3.33 mM), ammonium sulphate (16.7 mM), KCl (83.3 mM), 0.17% Tween 20 and Bst 2.0 (0.27 U/µL) in water was then prepared, adding the enzyme last. The solution was quickly vortexed and 12 µL was rapidly added to each PCR tube. The tubes were mixed by vortexing and incubated at 61.5 °C for 14 h followed by 85 °C for 20 min before rapidly cooling to 4 °C. The tubes were centrifuged and the supernatant analysed by 0.8% agarose gel electrophoresis. Any precipitates that formed were washed 3 times with H_2O, suspended in 20 µL of H_2O and 7.5 µL of the samples were analysed by 0.8% agarose gel electrophoresis. The samples were also imaged by SEM if DNA was detected.

Φ29 DNA polymerase screening with $MnCl_2$

The buffer stock solution (5 µL) and the $MnCl_2$ stock solution (6 µL) were added to a 200 µL PCR tube. An enzyme stock solution composed of T4 ligase cyclised T1 (0.67 µM), S1 (1.33 µM), dNTPs (4.44 mM), ammonium sulphate (22.2 mM), DTT (8.89 mM), and Φ29 (0.57 U/µL) in water was then prepared, adding the enzyme last. The solution was quickly vortexed and 9 µL was rapidly added to each PCR tube. The tubes were mixed by vortexing and incubated at 30 °C for 20 h followed by 65 °C for 10 min before rapidly cooling to 4 °C. The tubes were centrifuged and the supernatant analysed by agarose gel electrophoresis. Any precipitates that formed were washed 3 times with H_2O, and suspended in 20 µL of H_2O. 7.5 µL of the samples were analysed by 0.8% agarose gel electrophoresis. The samples were also imaged by SEM if DNA was detected.

Φ29 DNA polymerase screening with $CoCl_2$ and $ZnCl_2$

The buffer stock solution (5 µL) and either the $CoCl_2$ or $ZnCl_2$ stock solution (6 µL) were added to a 200 µL PCR tube. An enzyme stock solution composed of T4 ligase cyclised T1 (0.67 µM), S1 (1.33 µM), dNTPs (4.44 mM), ammonium sulphate (22.2 mM) and Φ29 (0.57 U/µL) in water was then prepared, adding the enzyme last. The solution was quickly vortexed and 9 µL was rapidly added to each PCR tube. The tubes were mixed by vortexing and incubated at 30 °C for 20 h followed by 65 °C for 10 min before rapidly cooling to 4 °C. The tubes were centrifuged and the supernatant was analysed by 0.8% agarose gel electrophoresis. Any precipitates that formed were washed 3 times with H_2O, suspended in 20 µL of H_2O and 7.5 µL of the samples were analysed by 0.8% agarose gel electrophoresis.

Comparison of DNA levels using SYBR Gold, SYBR Green I, and SYBR Green II

RCA reactions were performed as described above. 80 µL of water was then added to the RCA mixtures (5X dilution) and the samples were mixed thoroughly using a pipette. The precipitate was then collected by centrifugation and the supernatant carefully removed and kept. The precipitates

were then washed 3 times with 100 μL of water and incubated in 100 μL of TBE buffer overnight at room temperature to break down and dissolve any particles that formed. 5 μL of either the supernatant solution or the particle solution were then added to 100 μL of 1X TBE containing 1X SYBR dye and 0.1% Triton X-100 in a 96 well plate (Greiner, F-bottom (chimney well), black, Fluotrac, med. binding). Detergent was found to be necessary to prevent signal loss over time. The samples were incubated for 20 min with intermittent shaking (300 rpm, 1 min, five times) before reading the relative fluorescence using a CLARIOstar microplate reader. All samples were performed in triplicate and the standard deviations of these are given. The same gain and focal height were used for all readings measured using each individual dye allowing direct comparison. For SYBR Gold experiments the settings used were Excitation: 495-8; Dichroic filter: 514.2; Emission: 537-15. For SYBR Green I experiments the settings used were Excitation: 492-8; Dichroic filter: 514.2; Emission: 526-8. For SYBR Green II experiments the settings used were: Excitation: 492-8; Dichroic filter: 514.2; Emission: 526-8.

Preparation of inorganic material for IR studies

The inorganic components were prepared based on a literature procedure for the preparation of $Mn_3(PO_4)_2.3H_2O$.(1) 4 mL of either 10 mM KH_2PO_4 or $Na_2H_2P_2O_7$ buffered in 18.5 mM HEPES at pH 7.5 was added to 4 mL of either 10 mM $MnCl_2$, $CoCl_2$ or $ZnCl_2$ in water. The solutions were left at room temperature overnight then the resulting precipitate was collected by centrifugation, washed 5 times with MilliQ H_2O, and lyophilised before FTIR analysis.

Preparation of particles for magnetic studies

Preparation of Φ29 particles using 15 mM Mn^{2+}

A solution containing T4 ligase cyclised T1 (0.3 nmol), S1 (0.6 nmol), Tris buffer (250 μL, 200 mM, pH 8.0), $MnCl_2$ (300 μL, 50 mM), ammonium sulfate (10 μL, 1 M), and H_2O (215 μL) was heated to 95 °C and cooled to room temperature at a rate of 1 °C/min. dNTPs (10 mM, 200 μL) and Φ29 (25 μL, 10 000 U/mL) were added and the mixture incubated at 30 °C for 20 h. The sample was then heated to 65 °C for 10 min and cooled to room temperature. The resulting precipitate was collected by centrifugation and washed 5 times by resuspending in MilliQ water followed by collecting by centrifugation. The particles were analysed by SEM and agarose gel electrophoresis before the precipitate was dried under vacuum for 12 h and used in IR and magnetic studies. Yield: 0.65 mg of off-white precipitate.

Preparation of Φ29 particles using 10 mM Mn^{2+}

A solution containing T4 ligase cyclised T1 (0.3 nmol), S1 (0.6 nmol), Tris buffer (250 μL, 200 mM, pH 8.0), $MnCl_2$ (300 μL, 33.3 mM), ammonium sulfate (10 μL, 1 M), and H_2O (215 μL) was heated to 95 °C and cooled to room temperature at a rate of 1 °C/min. dNTPs (10 mM, 200 μL) and Φ29 (25 μL, 10 000 U/mL) were added and the mixture incubated at 30 °C for 20 h. The sample was then heated to 65 °C for 10 min and cooled to room temperature. The resulting precipitate was collected by

centrifugation and washed 5 times by resuspending in MilliQ water followed by collecting by centrifugation. The particles were analysed by SEM and agarose gel electrophoresis before the precipitate was dried under vacuum for 12 h and used in IR and magnetic studies. Yield: 0.8 mg of off-white precipitate.

Preparation of Φ29 particles using 15 mM Co^{2+}

A solution containing T4 ligase cyclised T1 (0.3 nmol), S1 (0.6 nmol), Tris buffer (250 µL, 200 mM, pH 7.0), $CoCl_2$ (300 µL, 50 mM), ammonium sulfate (10 µL, 1 M), and H_2O (215 µL) was heated to 95 °C and cooled to room temperature at a rate of 1 °C/min. dNTPs (10 mM, 200 µL) and Φ29 (25 µL, 10 000 U/mL) were added and the mixture incubated at 30 °C for 20 h. The sample was then heated to 65 °C for 10 min and cooled to room temperature. The resulting precipitate was collected by centrifugation and washed 5 times by resuspending in MilliQ water followed by collecting by centrifugation. The particles were analysed by SEM and agarose gel electrophoresis before the precipitate was dried under vacuum for 12 h and used in IR and magnetic studies. Yield: 0.6 mg of pink precipitate.

Preparation of Φ29 particles using 10 mM Co^{2+}

A solution containing T4 ligase cyclised T1 (0.3 nmol), S1 (0.6 nmol), Tris buffer (250 µL, 200 mM, pH 7.0), $CoCl_2$ (300 µL, 33.3 mM), ammonium sulfate (10 µL, 1 M), and H_2O (215 µL) was heated to 95 °C and cooled to room temperature at a rate of 1 °C/min. dNTPs (10 mM, 200 µL) and Φ29 (25 µL, 10 000 U/mL) were added and the mixture incubated at 30 °C for 20 h. The sample was then heated to 65 °C for 10 min and cooled to room temperature. The resulting precipitate was collected by centrifugation and washed 5 times by resuspending in MilliQ water followed by collecting by centrifugation. The particles were analysed by SEM and agarose gel electrophoresis before the precipitate was dried under vacuum for 12 h and used in IR and magnetic studies. Yield: 0.1 mg of pink precipitate.

Preparation of Bst particles using 15 mM Mn^{2+}

A solution containing T4 ligase cyclised T1 (0.3 nmol), S1 (0.6 nmol), Tris buffer (100 µL, 200 mM, pH 7.5), $MnCl_2$ (300 µL, 50 mM), ammonium sulfate (10 µL, 1 M), KCl (50 µL, 1 M), Tween 20 (10 µL, 10% v/v) and H_2O (310 µL) was heated to 95 °C and cooled to room temperature at a rate of 1 °C/min. dNTPs (10 mM, 200 µL) and Bst (20 µL, 8 000 U/mL) were added and the mixture incubated at 61.5 °C for 14 h. The sample was then heated to 85 °C for 20 min and cooled to room temperature. The resulting precipitate was collected by centrifugation and washed 5 times by resuspending in MilliQ water followed by collecting by centrifugation. The particles were analysed by SEM and agarose gel electrophoresis before the precipitate was dried under vacuum for 12 h and used in IR and magnetic studies. Yield: 1.0 mg of off-white precipitate.

Preparation of Bst particles using 10 mM Mn^{2+}

A solution containing T4 ligase cyclised T1 (0.3 nmol), S1 (0.6 nmol), Tris buffer (100 µL, 200 mM, pH 7.5), $MnCl_2$ (300 µL, 33.3 mM), ammonium sulfate (10 µL, 1 M), KCl (50 µL, 1 M), Tween 20 (10 µL, 10% v/v) and H_2O (310 µL) was heated to 95 °C and cooled to room temperature at a rate of 1 °C/min. dNTPs (10 mM, 200 µL) and Bst (20 µL, 8 000 U/mL) were added and the mixture incubated at 61.5 °C for 14 h. The sample was then heated to 85 °C for 20 min and cooled to room temperature. The resulting precipitate was collected by centrifugation and washed 5 times by resuspending in MilliQ water followed by collecting by centrifugation. The particles were analysed by SEM and agarose gel electrophoresis before the precipitate was dried under vacuum for 12 h and used in IR and magnetic studies. Yield: 0.2 mg of off-white precipitate.

Preparation of Bst particles using 15 mM Co^{2+}

A solution containing T4 ligase cyclised T1 (0.3 nmol), S1 (0.6 nmol), Tris buffer (100 µL, 200 mM, pH 7.0), $CoCl_2$ (300 µL, 50 mM), ammonium sulfate (10 µL, 1 M), KCl (50 µL, 1 M), Tween 20 (10 µL, 10% v/v) and H_2O (310 µL) was heated to 95 °C and cooled to room temperature at a rate of 1 °C/min. dNTPs (10 mM, 200 µL) and Bst (20 µL, 8 000 U/mL) were added and the mixture incubated at 61.5 °C for 14 h. The sample was then heated to 85 °C for 20 min and cooled to room temperature. The resulting precipitate was collected by centrifugation and washed 5 times by resuspending in MilliQ water followed by collecting by centrifugation. The particles were analysed by SEM and agarose gel electrophoresis before the precipitate was dried under vacuum for 12 h and used in IR and magnetic studies. Yield: 0.1 mg of purple precipitate.

Preparation of Bst particles using 10 mM Co^{2+}

A solution containing T4 ligase cyclised T1 (0.3 nmol), S1 (0.6 nmol), Tris buffer (100 µL, 200 mM, pH 7.0), $CoCl_2$ (300 µL, 33.3 mM), ammonium sulfate (10 µL, 1 M), KCl (50 µL, 1 M), Tween 20 (10 µL, 10% v/v) and H_2O (310 µL) was heated to 95 °C and cooled to room temperature at a rate of 1 °C/min. dNTPs (10 mM, 200 µL) and Bst (20 µL, 8 000 U/mL) were added and the mixture incubated at 61.5 °C for 14 h. The sample was then heated to 85 °C for 20 min and cooled to room temperature. The resulting precipitate was collected by centrifugation and washed 5 times by resuspending in MilliQ water followed by collecting by centrifugation. The particles were analysed by SEM and agarose gel electrophoresis before the precipitate was dried under vacuum for 12 h and used in IR and magnetic studies. Yield: 0.1 mg of purple precipitate.

Enzymatic stability studies

Particle solutions (10 µL) were first centrifuged and the water removed. The pellets were then suspended in 20 µL of DMEM containing 10% FBS and incubated at 37 °C for a set time (1 h, 6 h, 12 h, 24 h and 36 h). In accordance with the literature, λ-DNA (1 µL of 500 µg/mL, NEB) was incubated under the same conditions as a control.(2) After incubation the samples were analysed by 0.8% agarose gel electrophoresis. The relative band intensities were measured with ImageJ and used to

compare DNA degradation. Particles from the 36 h incubation sample were collected by centrifugation, washed with water 3 times and imaged by SEM to determine structural integrity.

Supplementary figures

Formation of Magnesium DNA nanoflowers using Bst 2.0 polymerase

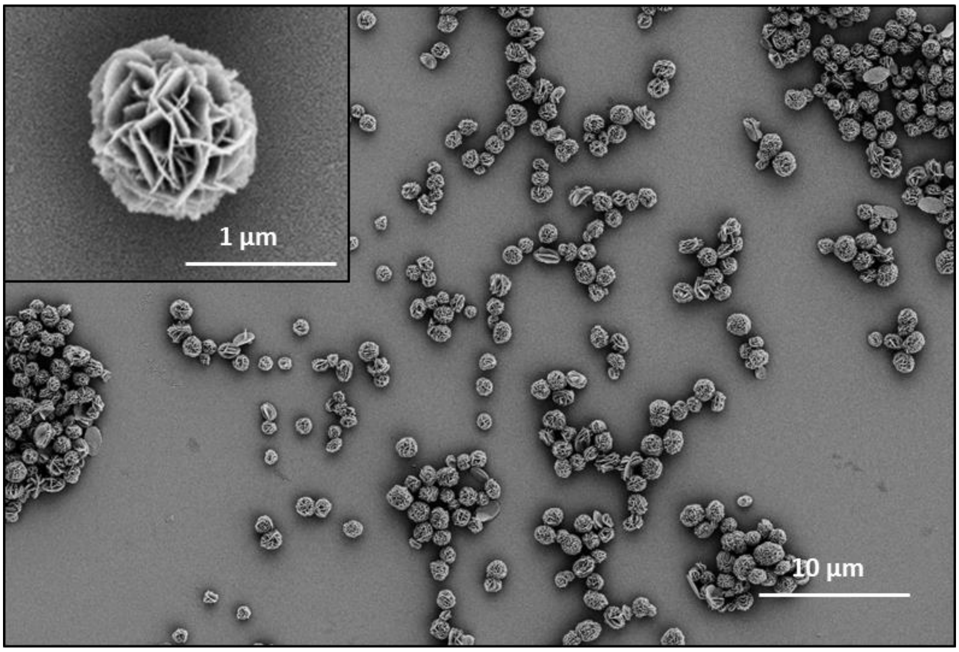

Figure S1. SEM image of precipitate formed during Bst catalysed RCA using T4 ligase cyclised template T1. The RCA reaction mixture was supplemented with $MgSO_4$ to give a final Mg^{2+} concentration of 15 mM. Inset) High-magnification SEM image showing the particle morphology.

RCA using CircLigase II cyclised templates

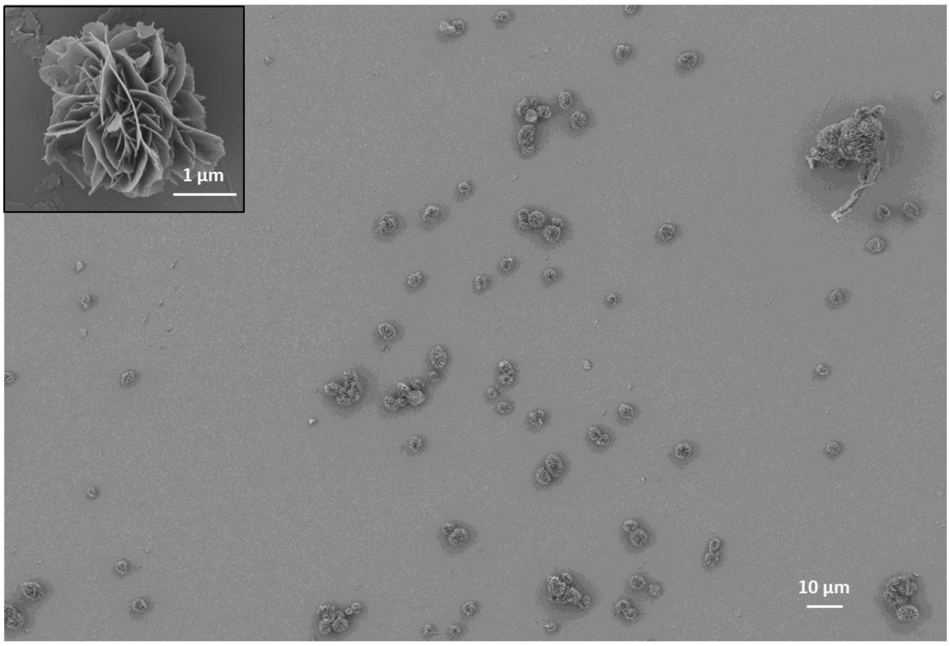

Figure S2. SEM image of precipitate formed during Bst catalysed RCA using CircLigase II cyclised template T1. This shows the sharp defined morphology of the MnDNFs. Inset) High-magnification SEM image showing the particle morphology. The $MgSO_4$ in the amplification buffer was substituted with $MnCl_2$. Average size of particles measured using ImageJ: 3.6 ± 1.2 μm, *n* = 90.

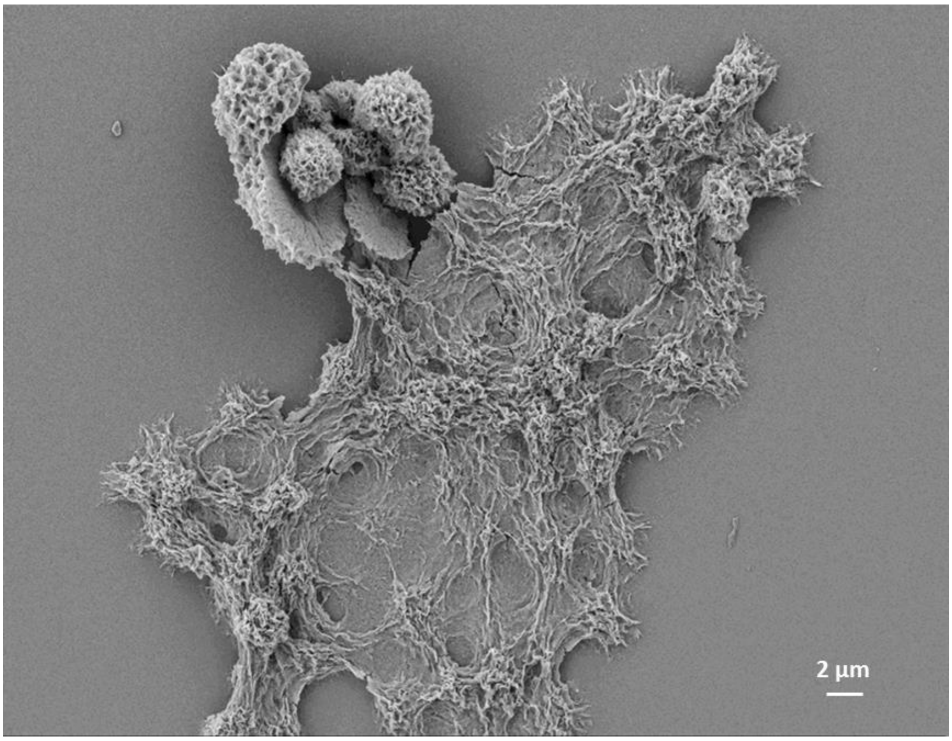

Figure S3. SEM image of precipitate formed during Φ29 catalysed RCA using CircLigase II cyclised template T1 that shows the softer appearance and undefined morphology compared to Fig S1. The $MgCl_2$ in the amplification buffer was substituted with $MnCl_2$.

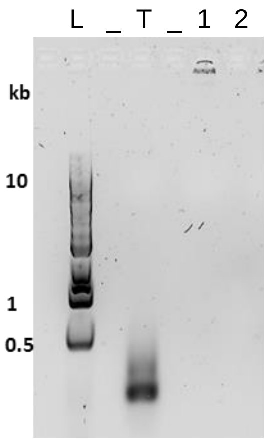

Figure S4. Agarose gel electrophoresis analysis of the precipitates formed during Bst and Φ29 catalysed RCA using CircLigase II cyclised template T1. The Mg^{2+} sources in the amplification buffers were substituted with $MnCl_2$. L) 1 kb DNA ladder; T) template T1; Lane 1) analysis of the precipitates that formed during Φ29 catalysed RCA; Lane 2) analysis of the precipitates that formed during Bst catalysed RCA. This result shows that the precipitates from Bst RCA did not contain DNA whereas the precipitates from Φ29 RCA did.

Agarose gel analysis for $MnCl_2$ screening experiments

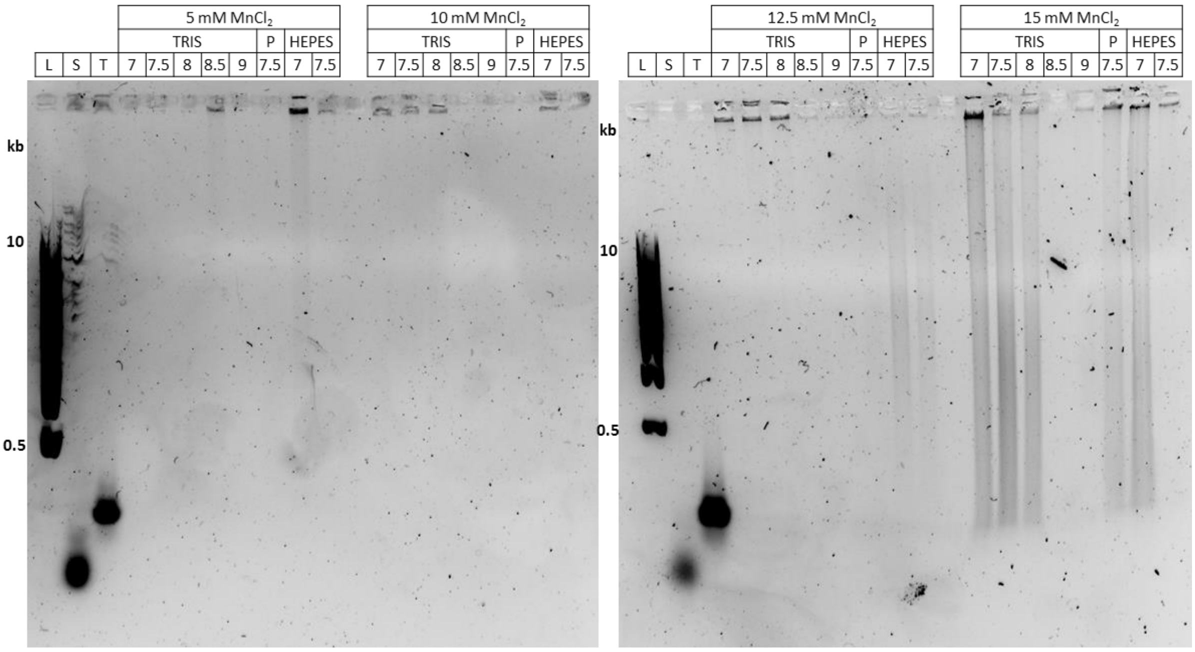

Figure S5. Agarose gel electrophoresis analysis of precipitates that form during Bst catalysed RCA with $MnCl_2$ as the cofactor. L) 1 kb DNA ladder; S) splint S1; T) template T1; P) PIPES; numbers above lanes indicate the pH of the buffer used. These gels show how the buffer used, the concentration of $MnCl_2$ and the pH of the amplification step control the DNA loading of the precipitates formed. Samples with DNA present were then analysed by SEM. DNA is only seen in precipitates at pH 8 and lower.

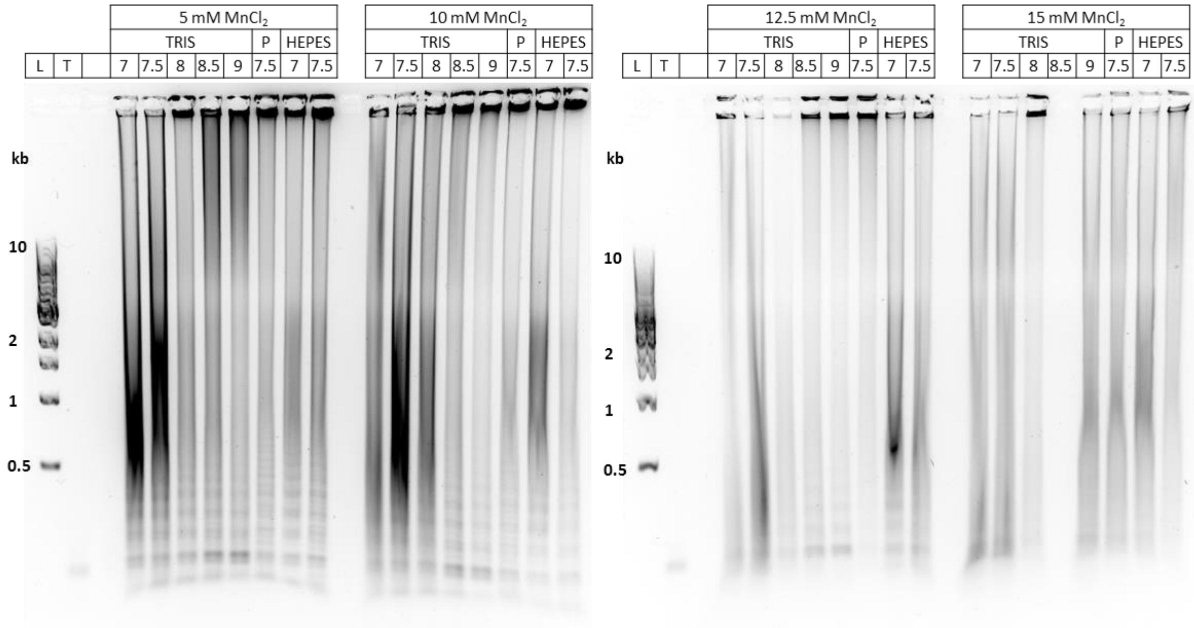

Figure S6. Agarose gel electrophoresis analysis of supernatants from Bst catalysed RCA with $MnCl_2$ as the cofactor. L) 1 kb DNA ladder; T) template T1; P) PIPES; numbers above lanes indicate the pH of the buffer used. These gels show how the buffer used, the concentration of $MnCl_2$ and the pH of the amplification step affect Bst efficiency and processivity. Higher pH results in the formation of longer free DNA in the supernatant.

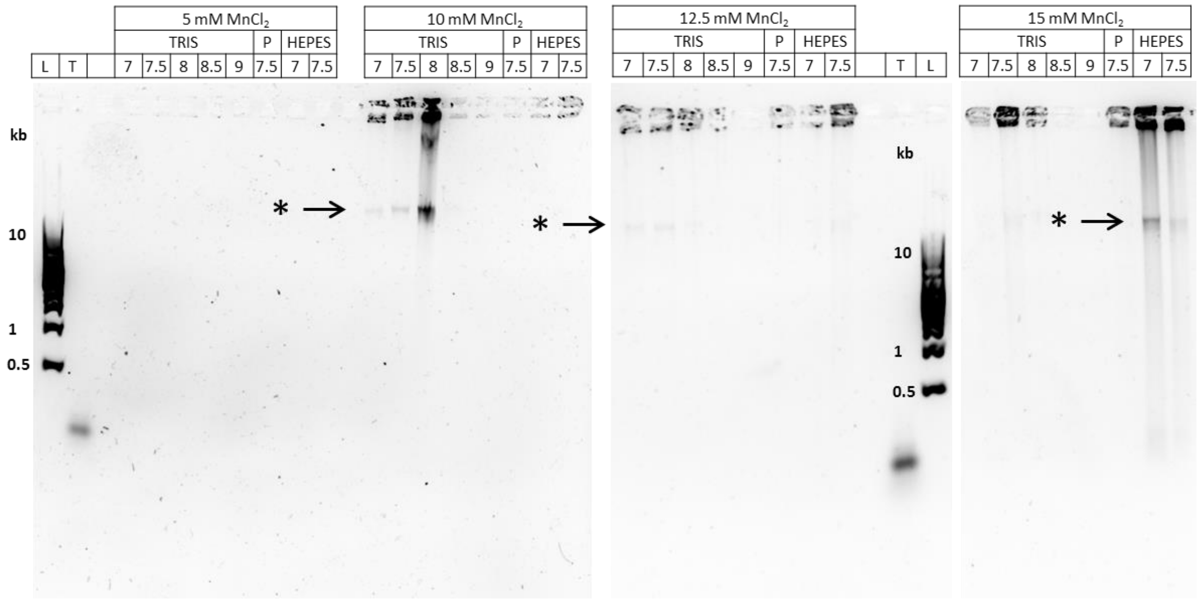

Figure S7. Agarose gel electrophoresis analysis of precipitates that form during Φ29 catalysed RCA with $MnCl_2$ as the cofactor. L) 1 kb DNA ladder; T) template T1; P) PIPES; *) the appearance of a second band matches with previous literature, suggesting that this is a double stranded product.(3) Numbers above lanes indicate the pH of the buffer used. These gels show how the buffer used, the concentration of $MnCl_2$ and the pH of the amplification step control the DNA loading of the precipitates formed. Samples with DNA present were then analysed by SEM. DNA is only seen in precipitates formed at pH 8 or lower.

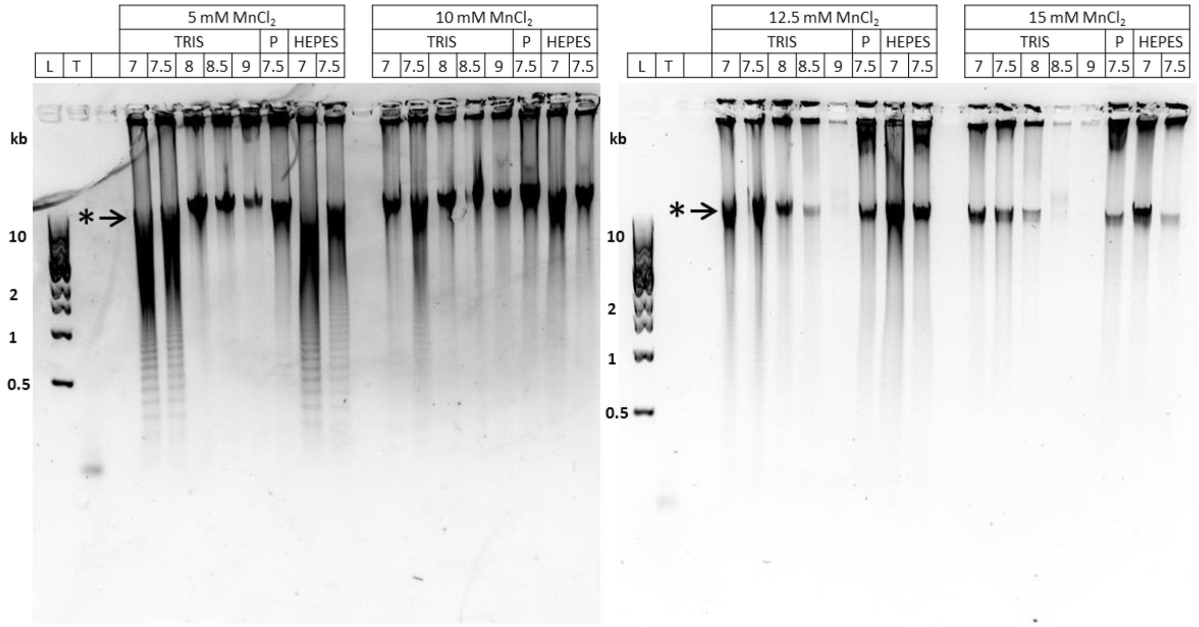

Figure S8. Agarose gel electrophoresis analysis of supernatants from Φ29 catalysed RCA with $MnCl_2$ as the cofactor. L) 1 kb DNA ladder; T) template T1; P) PIPES; *) the appearance of a second band matches with previous literature, suggesting that this is a double stranded product.(3) Numbers above lanes indicate the pH of the buffer used. These gels show how the buffer used, the concentration of $MnCl_2$ and the pH of the amplification step affect Φ29 efficiency and processivity. Lower pH appears to result in more efficient RCA.

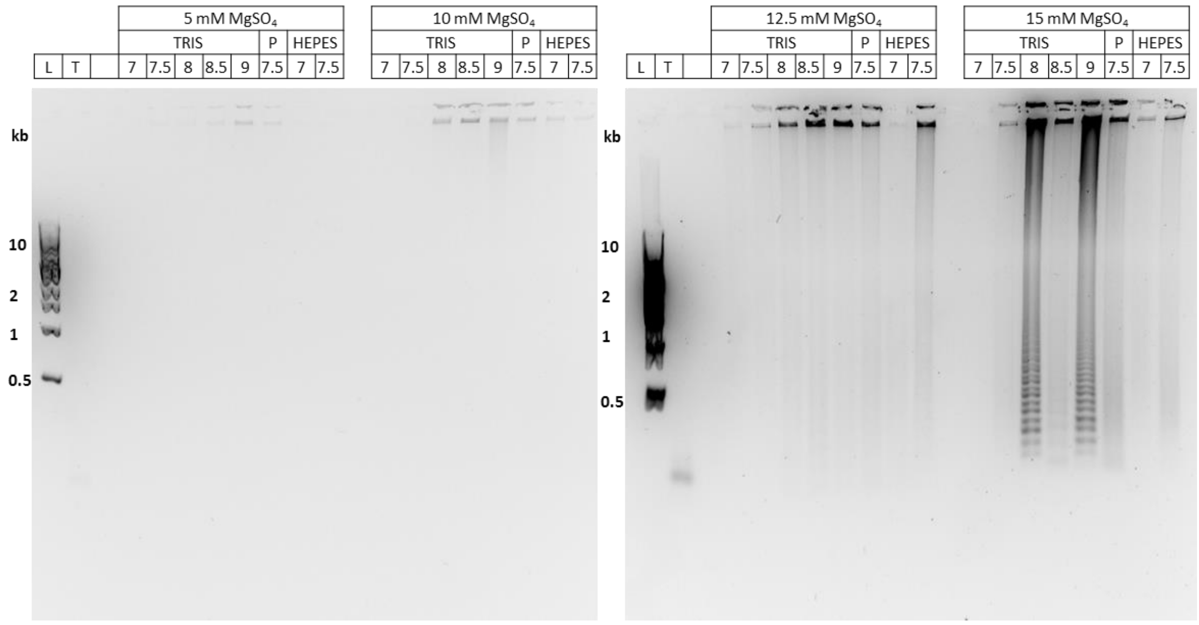

Figure S9. Agarose gel electrophoresis analysis of precipitates that form during Bst catalysed RCA with $MgSO_4$ as the cofactor. L) 1 kb DNA ladder; Template T1; P) PIPES; numbers above lanes indicate the pH of the buffer used. These gels show how the buffer used, the concentration of $MgSO_4$ and the pH of the amplification step control the DNA loading of the precipitates formed. Samples with DNA present were then analysed by SEM. In contrast with the $MnCl_2$ RCA reactions, the $MgSO_4$ precipitates appear to incorporate more DNA with increasing pH.

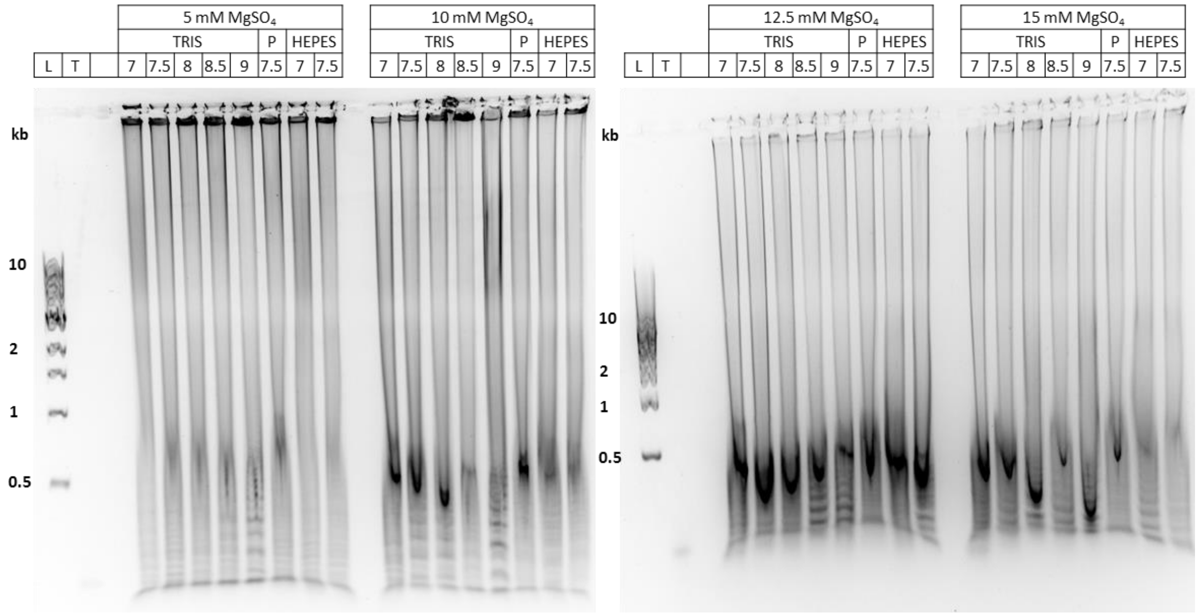

Figure S10. Agarose gel electrophoresis analysis of supernatants from Bst catalysed RCA with $MgSO_4$ as the cofactor. L) 1 kb DNA ladder; T) template T1; P) PIPES; numbers above lanes indicate the pH of the buffer used. These gels show how the buffer used, the concentration of $MgSO_4$ and the pH of the amplification step affect Bst efficiency and processivity. Lower concentrations of $MgSO_4$ appear to result in greater RCA processivity.

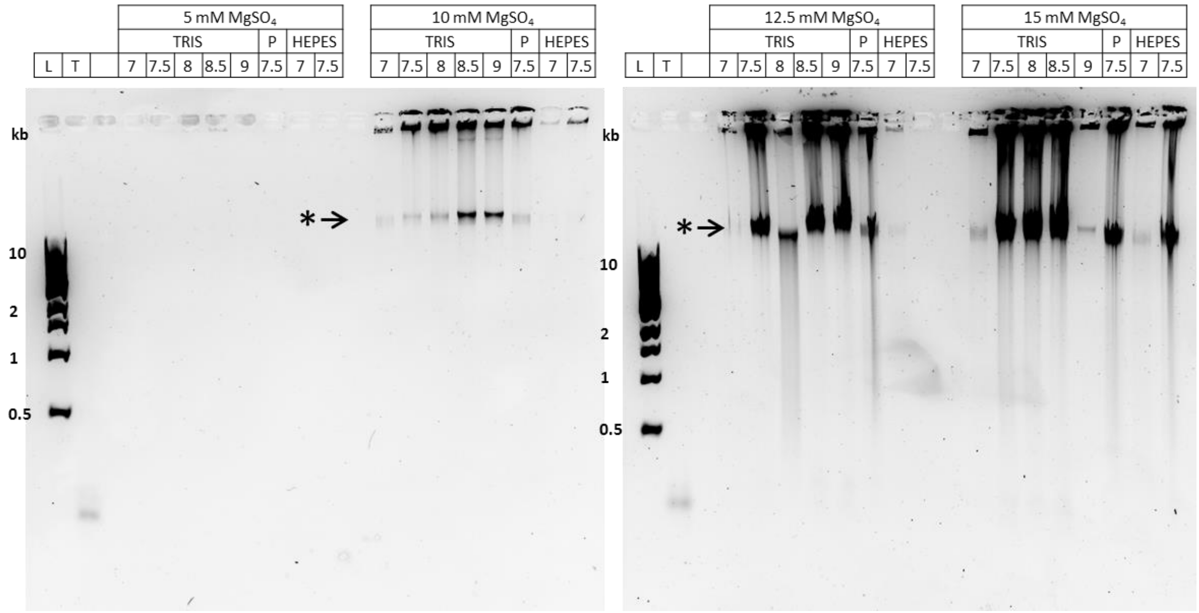

Figure S11. Agarose gel electrophoresis analysis of precipitates that form during Φ29 catalysed RCA with $MgSO_4$ as the cofactor. L) 1 kb DNA ladder; T) template T1; P) PIPES; *) the appearance of a second band matches with previous literature, suggesting that this is a double stranded product.(3) Numbers above lanes indicate the pH of the buffer used. These gels show how the buffer used, the concentration of $MgSO_4$ and the pH of the amplification step control the DNA loading of the precipitates formed. Samples with DNA present were then analysed by SEM. DNA is incorporated best into the particles at pH 8.5-9, except at the higher concentration of $MgSO_4$ (15 mM) where pH 7.5, 8 and 8.5 appear the best.

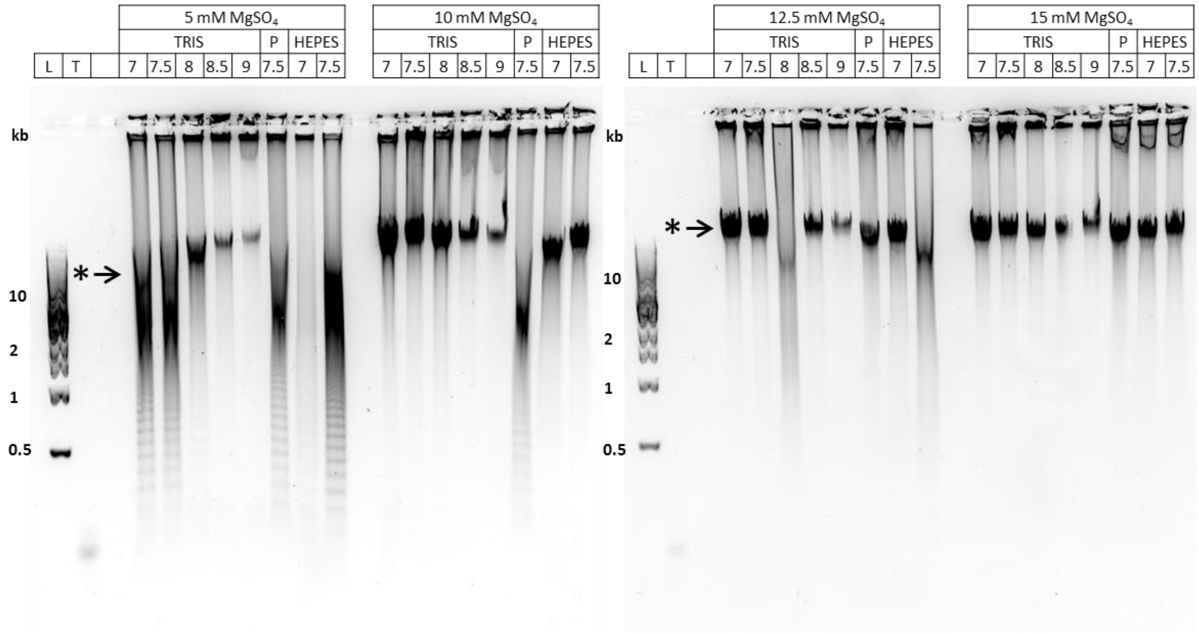

Figure S12. Agarose gel electrophoresis analysis of supernatants from Φ29 catalysed RCA with $MgSO_4$ as the cofactor. L) 1 kb DNA ladder; T) template T1; P) PIPES; *) the appearance of a second band matches with previous literature, suggesting that this is a double stranded product.(3) Numbers above lanes indicate the pH of the buffer used. These gels show how the buffer used, the concentration of $MgSO_4$ and the pH of the amplification step affect Φ29 efficiency and processivity.

The RCA reactions at lower pH appear to produce more free DNA present in the supernatant, this is possibly due to the incorporation of DNA into the precipitate at higher pH (Fig S10).

Supplementary MnDNF SEM images

SEM images showing the effect of buffer on particle morphology

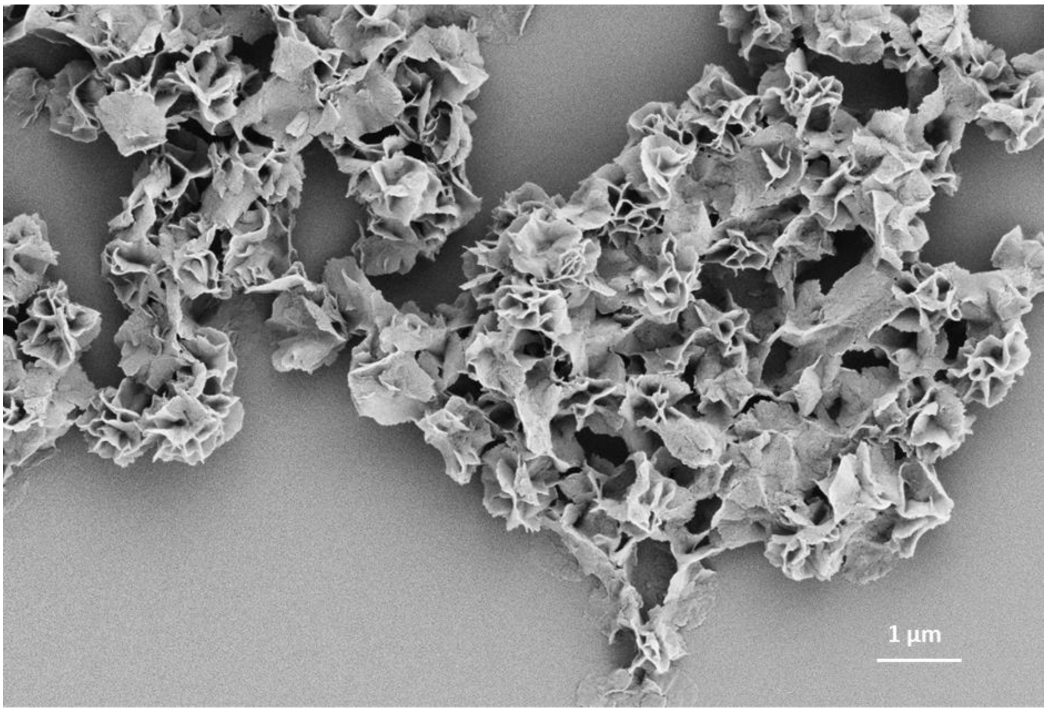

Figure S13. Representative SEM image of the precipitate formed during Bst catalysed RCA in the presence of 20 mM $MnCl_2$. This image shows that increasing the concentration of $MnCl_2$ past 15 mM in the amplification buffer results in a large mesh formed of sheets.

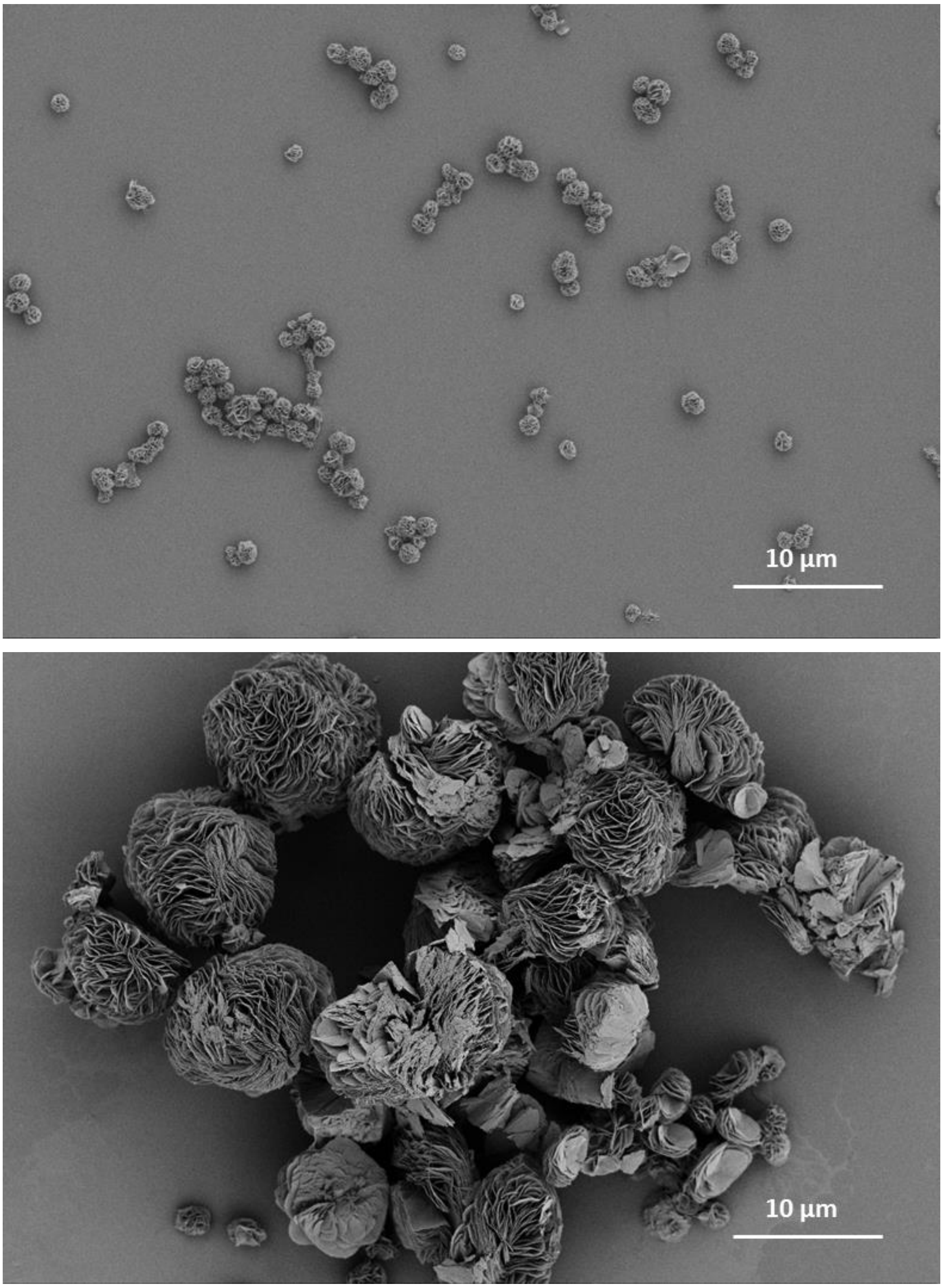

Figure S14. Representative SEM images of the precipitate formed during Φ29 catalysed RCA in the presence of 10 mM $MnCl_2$. Top) Tris buffer pH 8; Bottom) HEPES pH 7.5. Scale bar: 10 µm. These images show that HEPES buffer produces particles with a much broader size range than Tris buffer during Φ29 catalysed RCA in the presence of 10 mM $MnCl_2$.

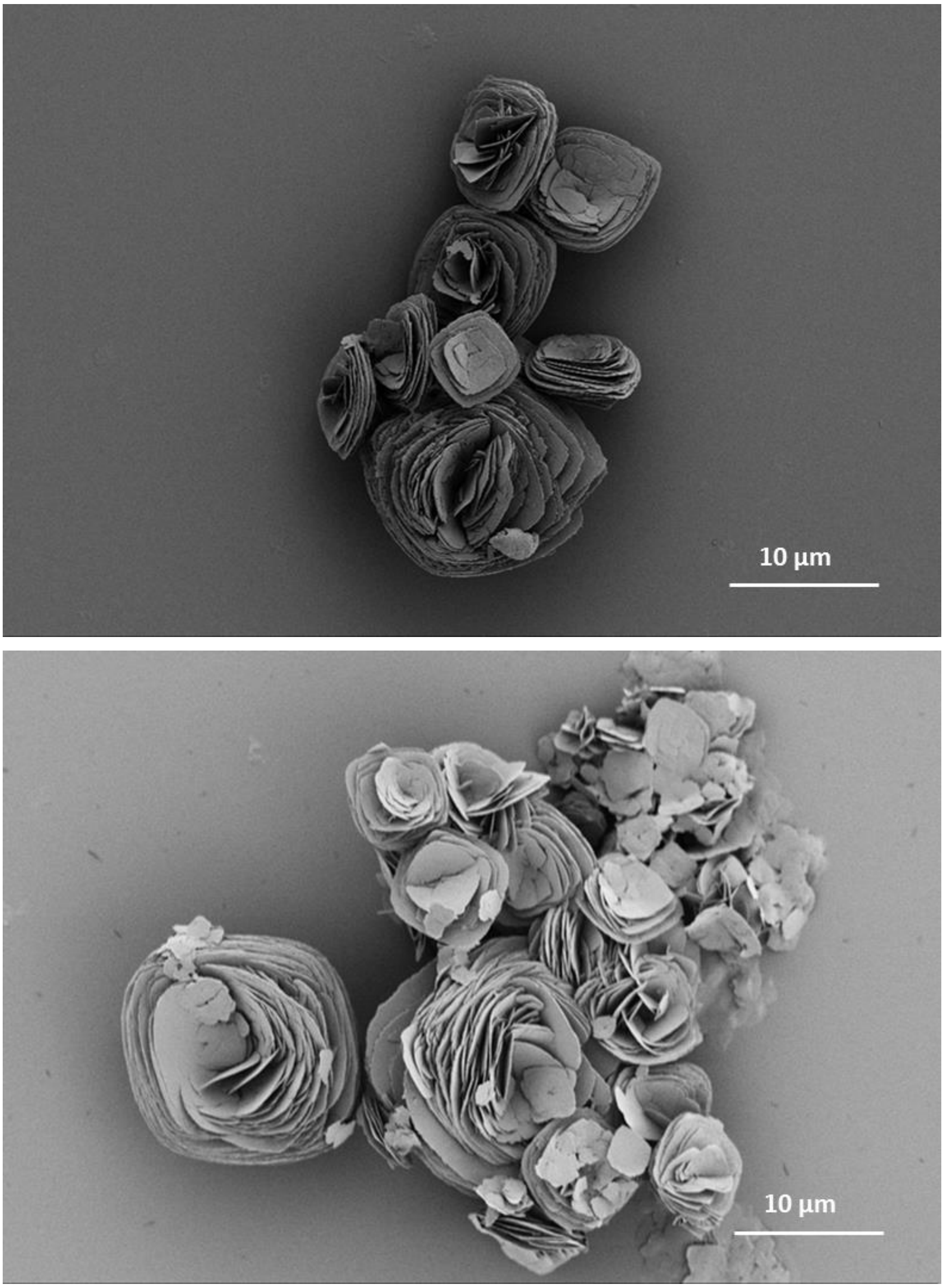

Figure S15. Representative SEM images of the precipitate formed during Φ29 catalysed RCA in the presence of 5 mM $MnCl_2$. Top) Tris buffer pH 8; Bottom) HEPES pH 7.5. Scale bar: 10 µm. These images show that HEPES buffer produces particles of similar morphology but with a much broader size range than Tris buffer during Φ29 catalysed RCA in the presence of 5 mM $MnCl_2$.

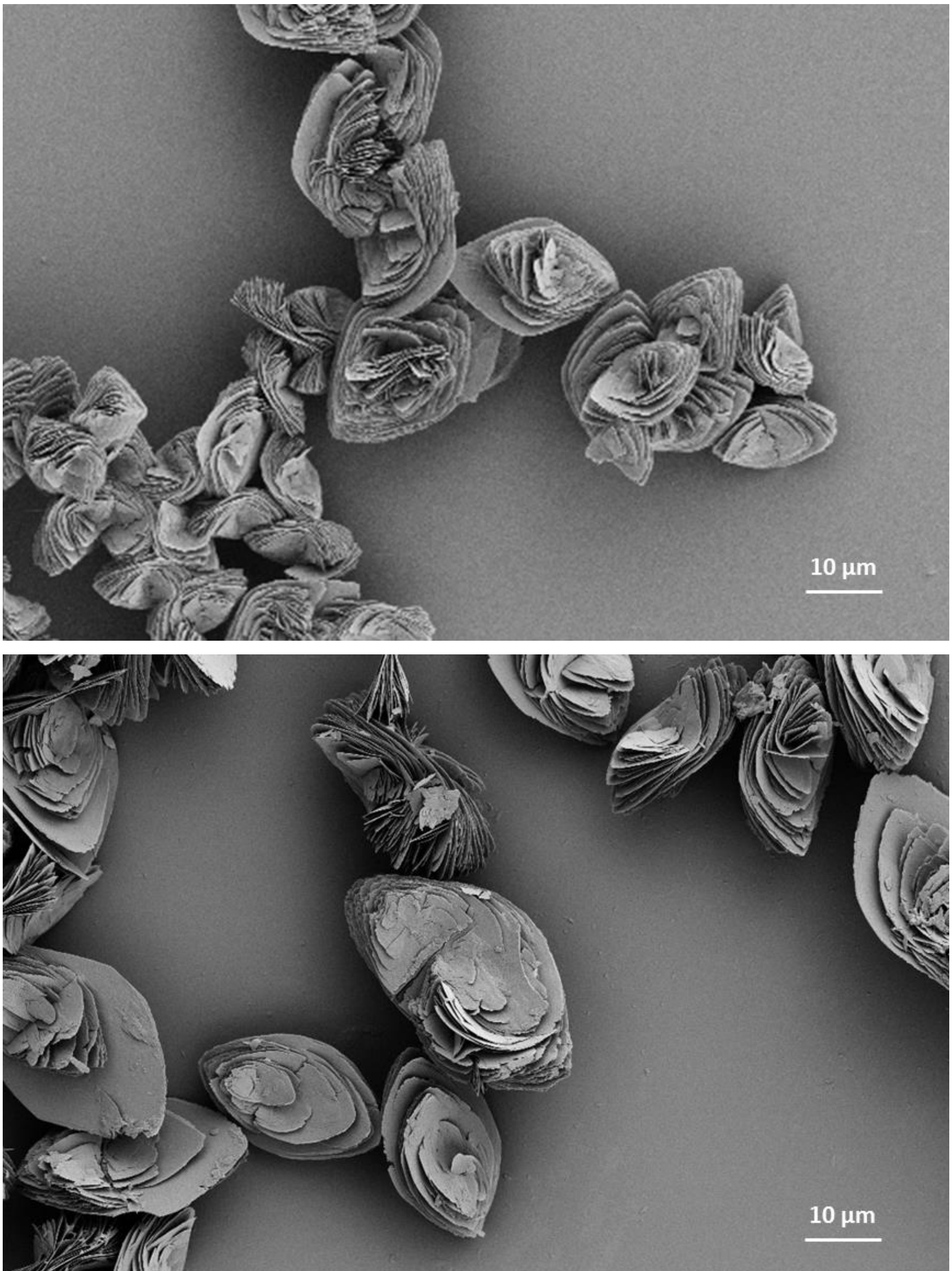

Figure S16. Representative SEM images of the precipitate formed during Bst catalysed RCA in the presence of 5 mM $MnCl_2$. Top) Tris buffer pH 8; Bottom) HEPES pH 7.5. Scale bar: 10 µm. These images show that HEPES buffer produces similar particles to Tris buffer for Bst RCA in the presence of 5 mM $MnCl_2$.

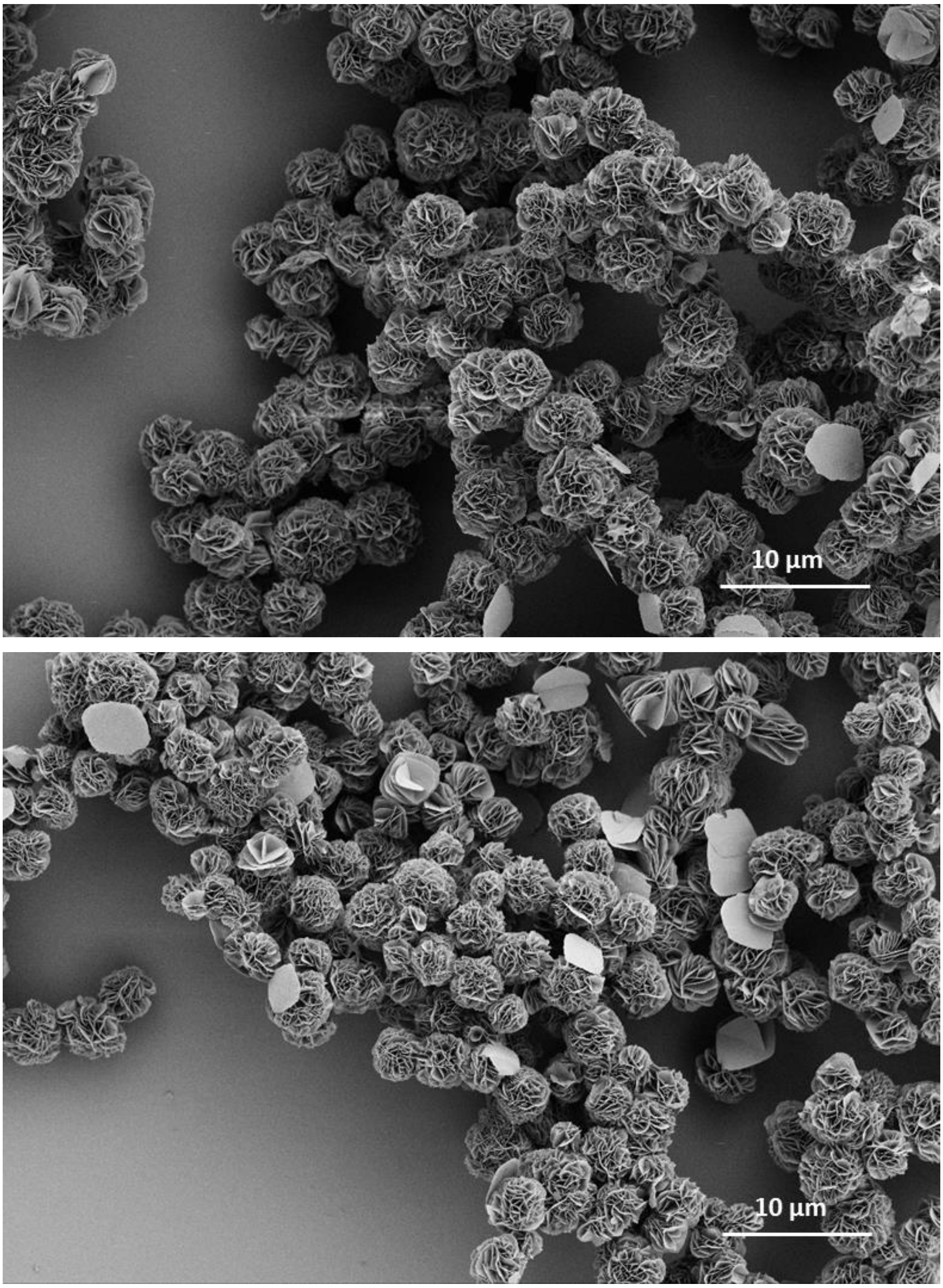

Figure S17. Representative SEM images of the precipitate formed during Bst catalysed RCA in the presence of 15 mM $MnCl_2$. Top) no ammonium sulphate; Bottom) 10 mM ammonium sulfate. Scale bar: 10 µm. These images suggest that ammonium sulfate does not affect the morphology of the particles.

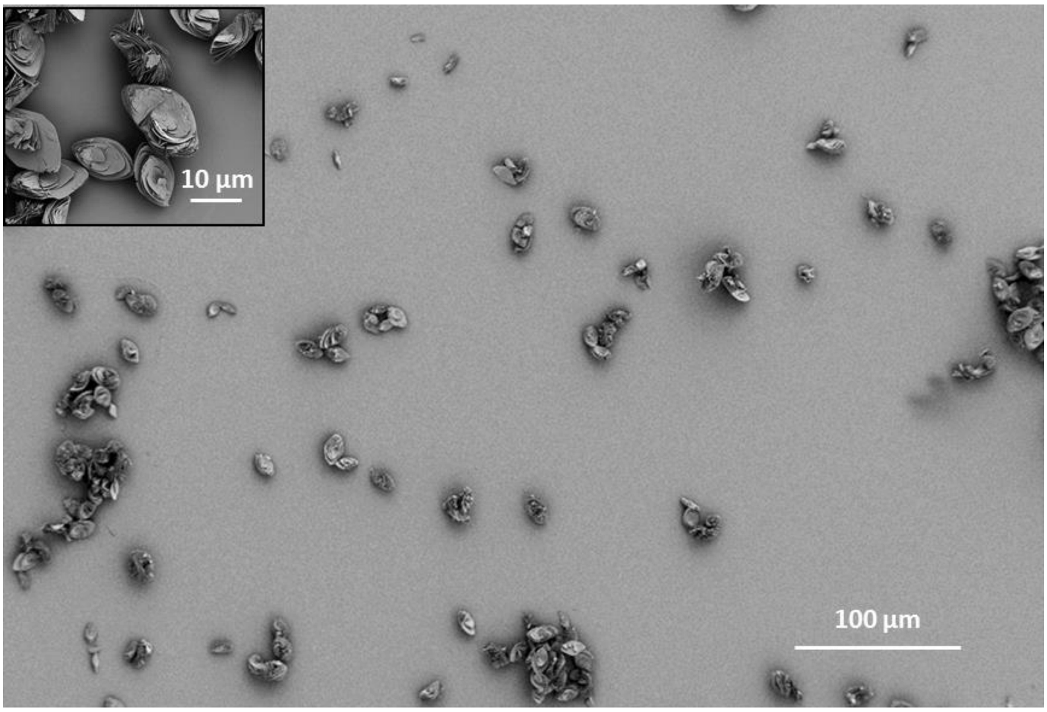

Figure S18. SEM image used to determine the size distribution of the precipitate formed during Bst catalysed RCA in the presence of 5 mM $MnCl_2$. ImageJ was used to measure the average point-to-point length: 15.8 ± 2.7 µm, *n* = 60. Only structures where both ends are clear were measured.

SEM images used to determine particle size

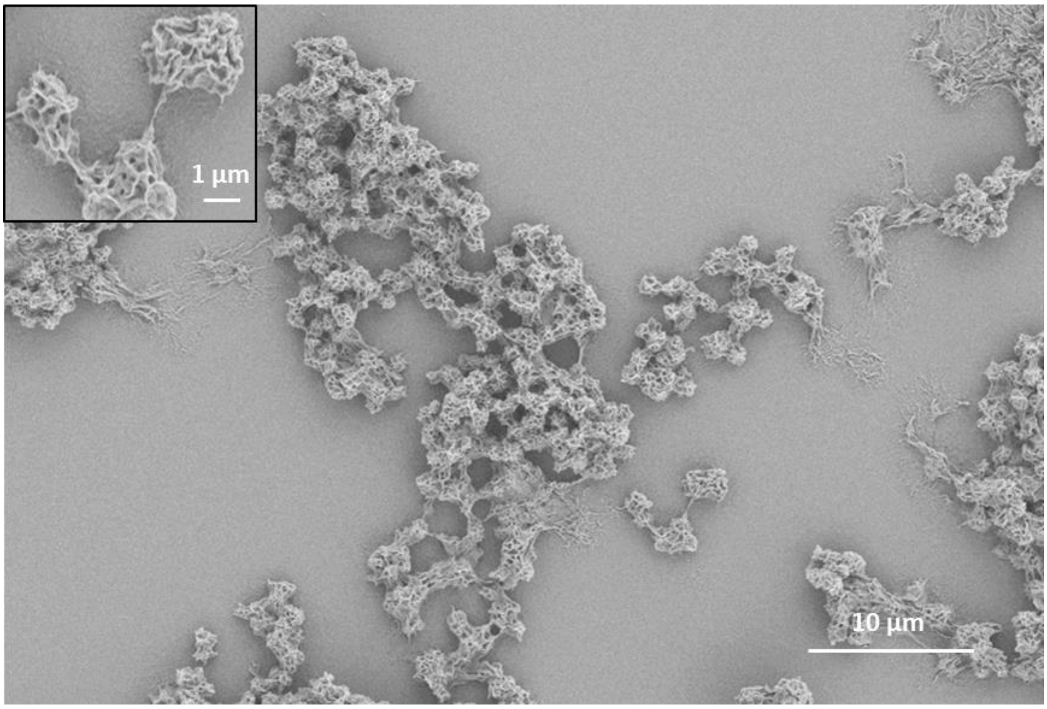

Figure S19. SEM image used to determine the size distribution of the precipitate formed during Bst catalysed RCA in the presence of 10 mM $MnCl_2$. ImageJ was used to measure the average globular structure size: 1.4 ± 0.5 µm; *n* = 26.

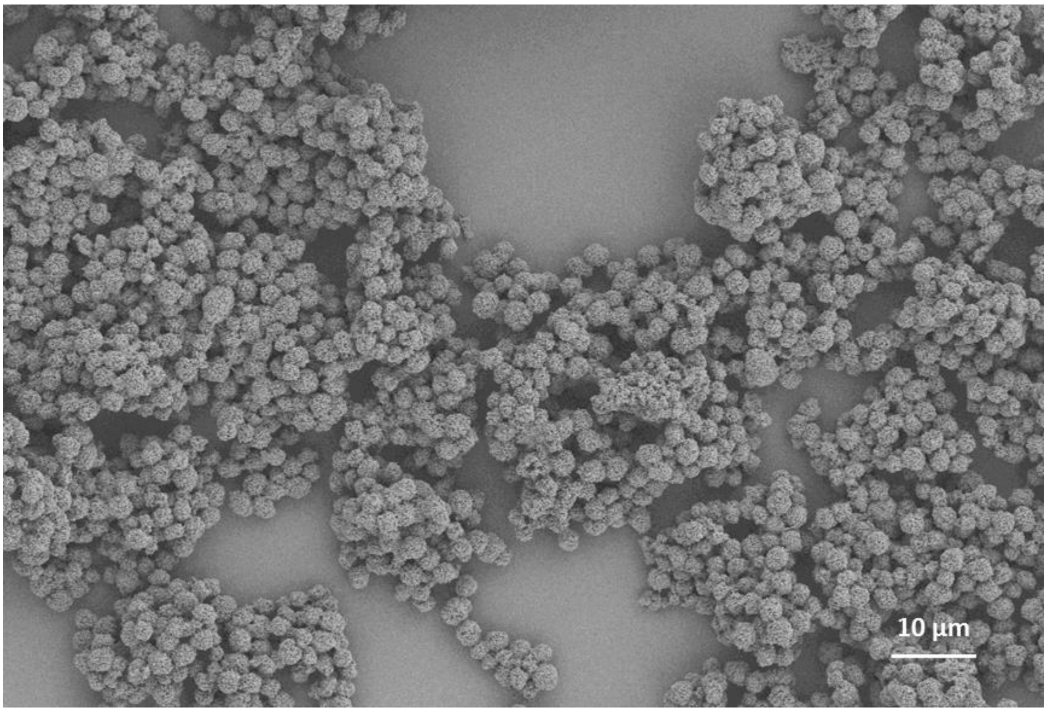

Figure S20. SEM image used to determine the size distribution of the precipitate formed during Bst catalysed RCA in the presence of 15 mM $MnCl_2$. ImageJ was used to measure the average diameter of particles where the whole structure could be seen: 2.6 ± 0.3 µm, *n* = 93.

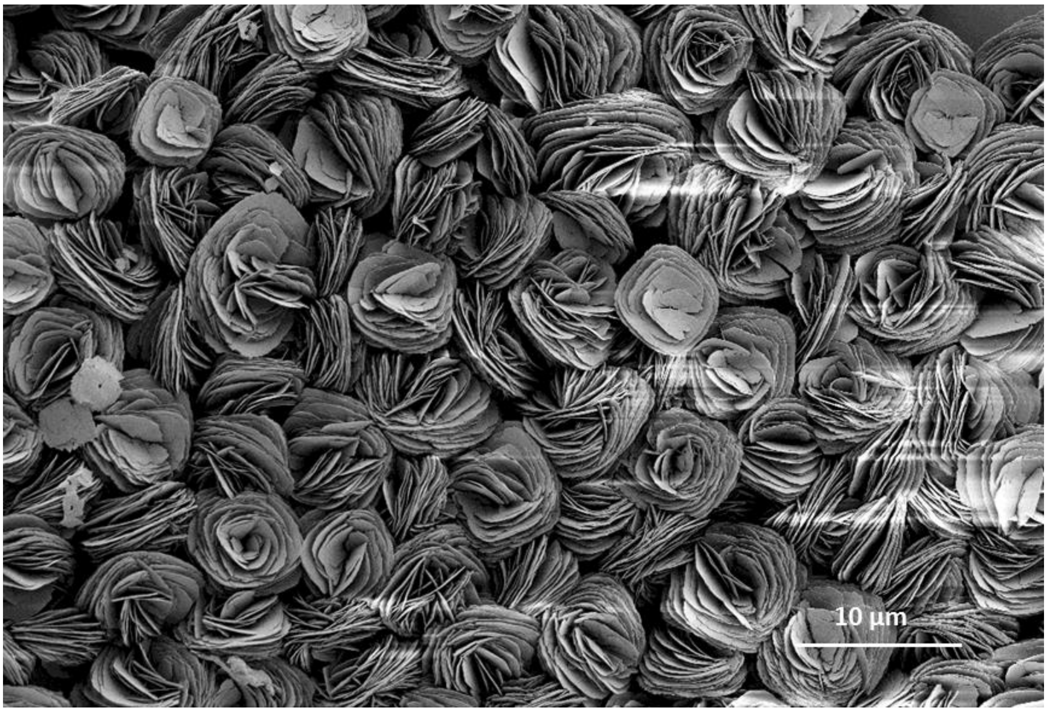

Figure S21. SEM image used to determine the size distribution of the precipitate formed during Φ29 catalysed RCA in the presence of 5 mM $MnCl_2$. ImageJ was used to measure the average diagonal length of the particles where the whole structure could be seen: 8.8 ± 1.2 µm, *n* = 20.

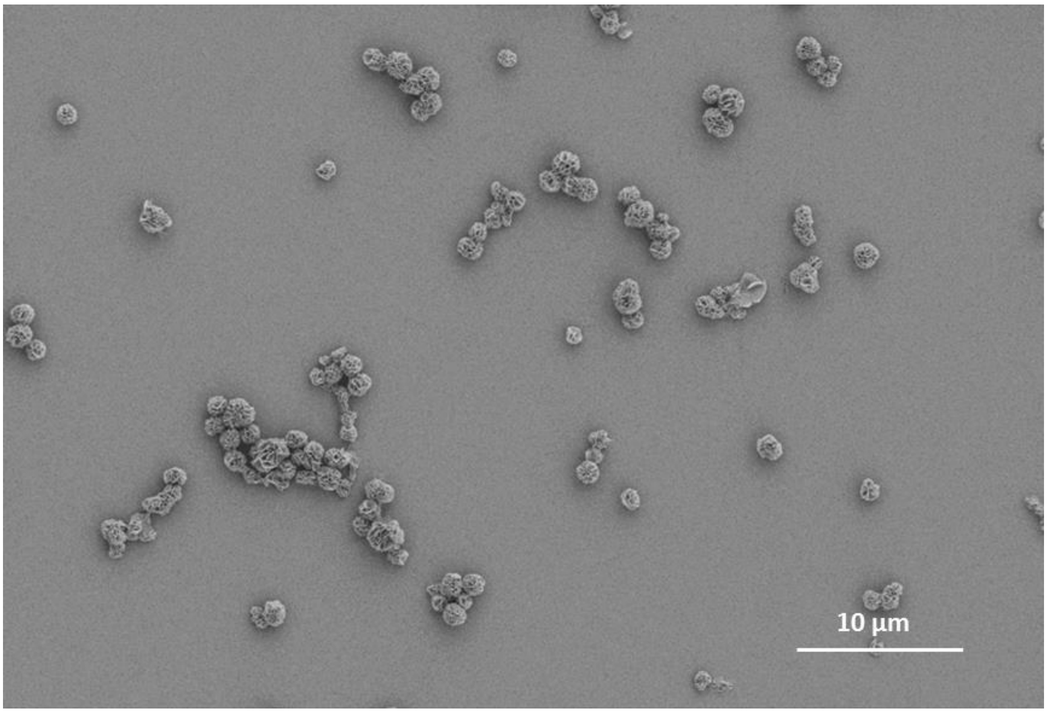

Figure S22. SEM image used to determine the size distribution of the precipitate formed during Φ29 catalysed RCA in the presence of 10 mM $MnCl_2$. ImageJ was used to measure the average diameter of the particles: 1.3 ± 0.2 µm, *n* = 72.

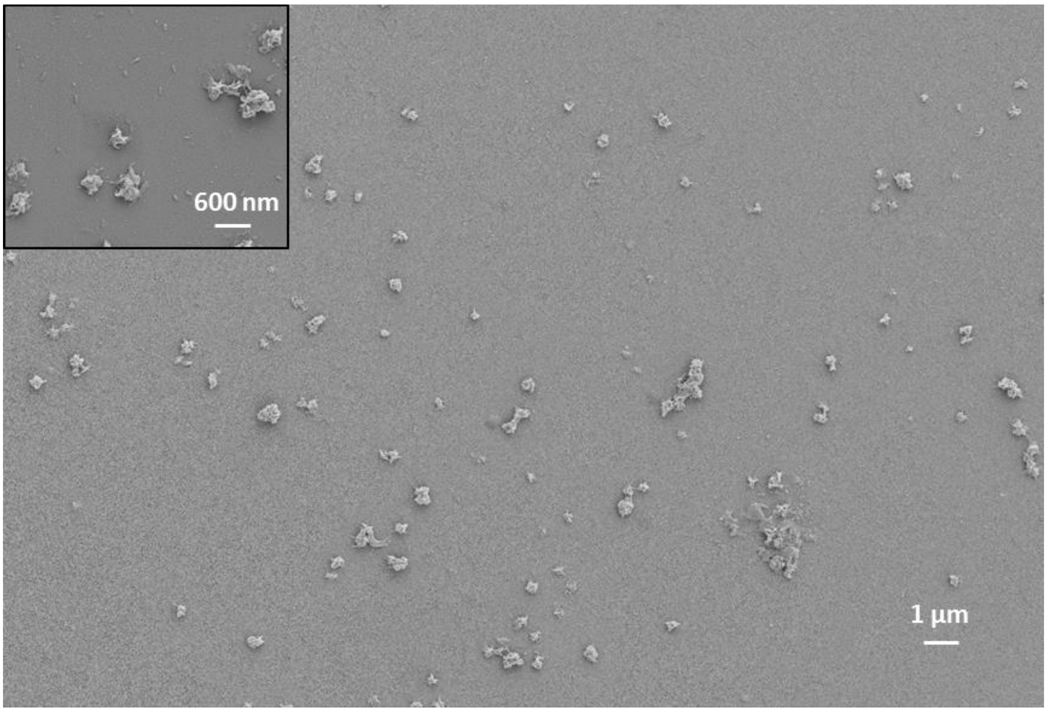

Figure S23. SEM image used to determine the size distribution of the precipitate formed during Φ29 catalysed RCA in the presence of 15 mM $MnCl_2$. ImageJ was used to measure the average diameter of the particles: 312 ± 6 nm, *n* = 111.

SYBR green I and SYBR green II analysis of DNA levels in MnDNFs

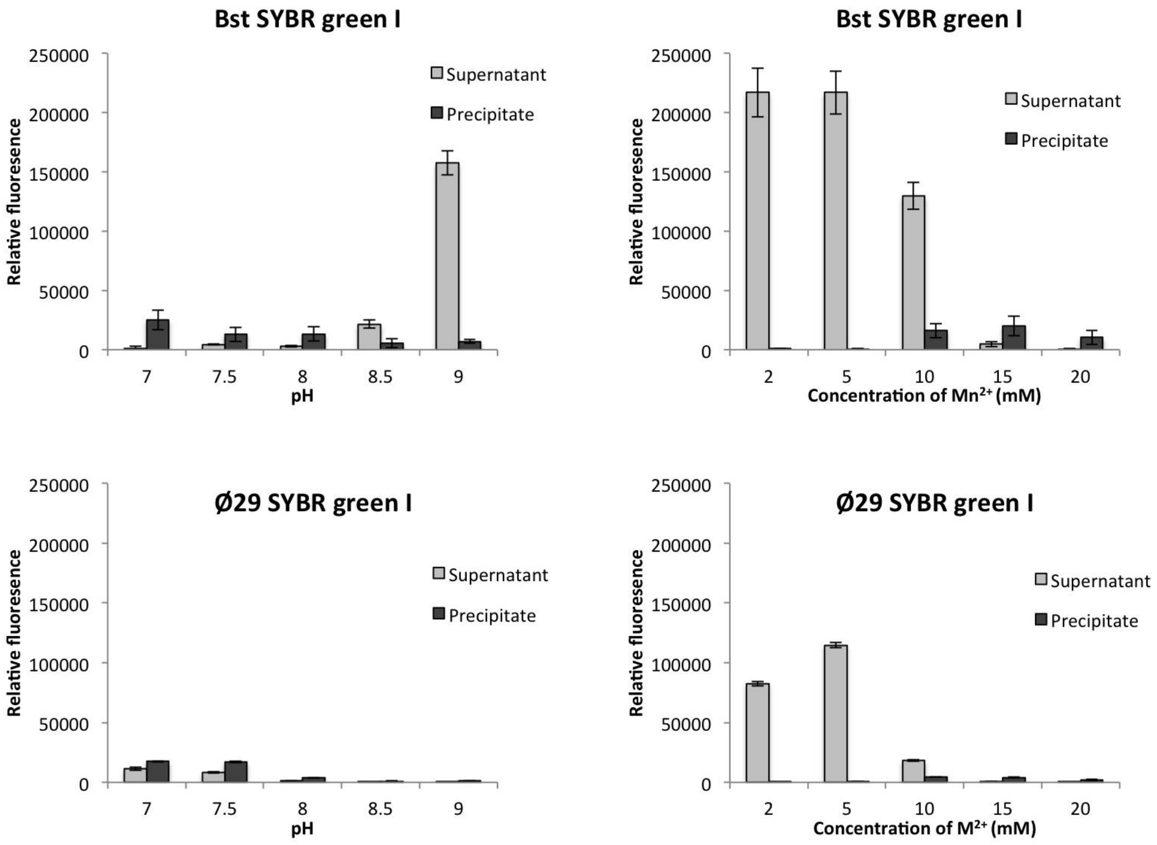

Figure S24. Fluorescence-based analysis comparing the levels of free DNA and DNA incorporated into the nanoconstructs as a function of enzyme, pH, and Mn^{2+} concentration. Samples were broken down using TBE to release DNA prior to incubation with SYBR green I. A plate reader was then used to compare the DNA levels. The effect of pH on Φ29 RCA was compared at 15 mM $MnCl_2$ and the effect of concentration was evaluated at pH 8.0. The effect of pH on Bst was determined at 15 mM $MnCl_2$ and the effect of concentration was evaluated at pH 7.0.

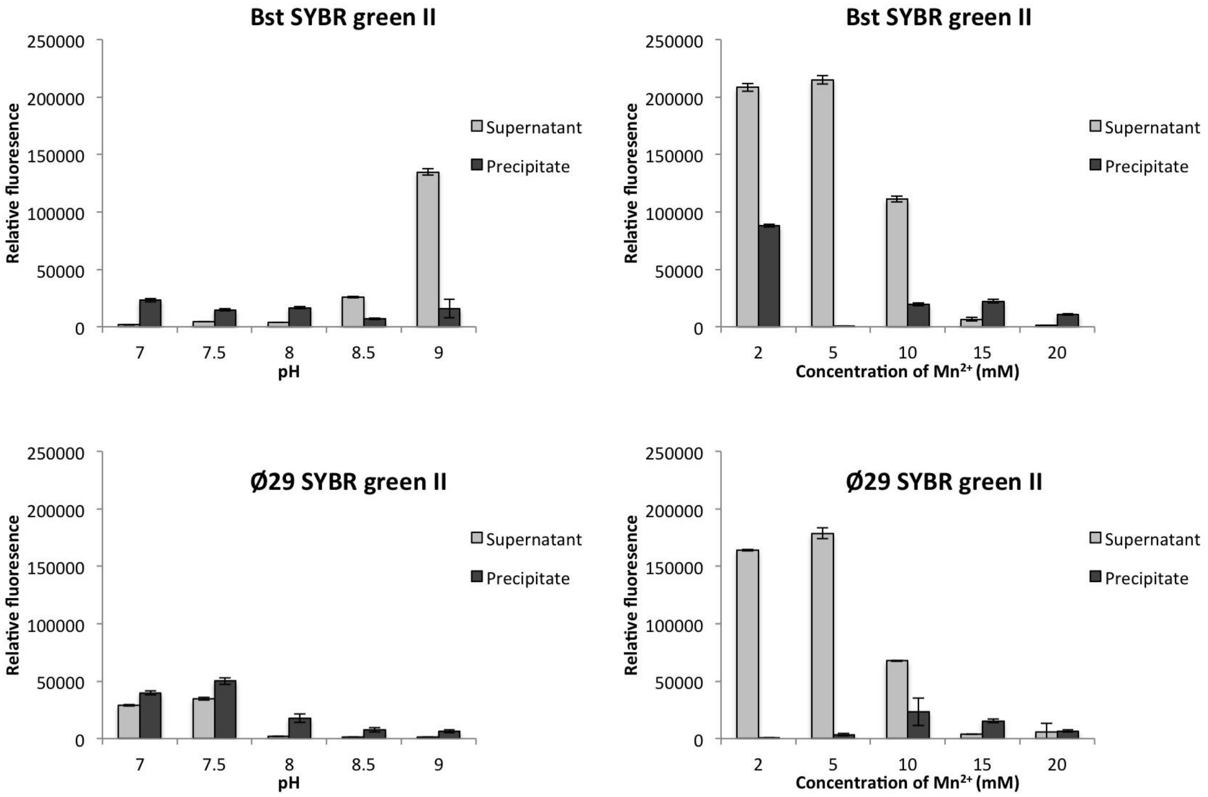

Figure S25. Fluorescence-based analysis comparing the levels of free DNA and DNA incorporated into the nanoconstructs as a function of enzyme, pH, and Mn^{2+} concentration. Samples were broken down using TBE to release DNA prior to incubation with SYBR green II. A plate reader was then used to compare the DNA levels. The effect of pH on Φ29 RCA was compared at 15 mM $MnCl_2$ and the effect of concentration was evaluated at pH 8.0. The effect of pH on Bst was determined at 15 mM $MnCl_2$ and the effect of concentration was evaluated at pH 7.0.

SYBR gold analysis of DNA levels in MgDNFs

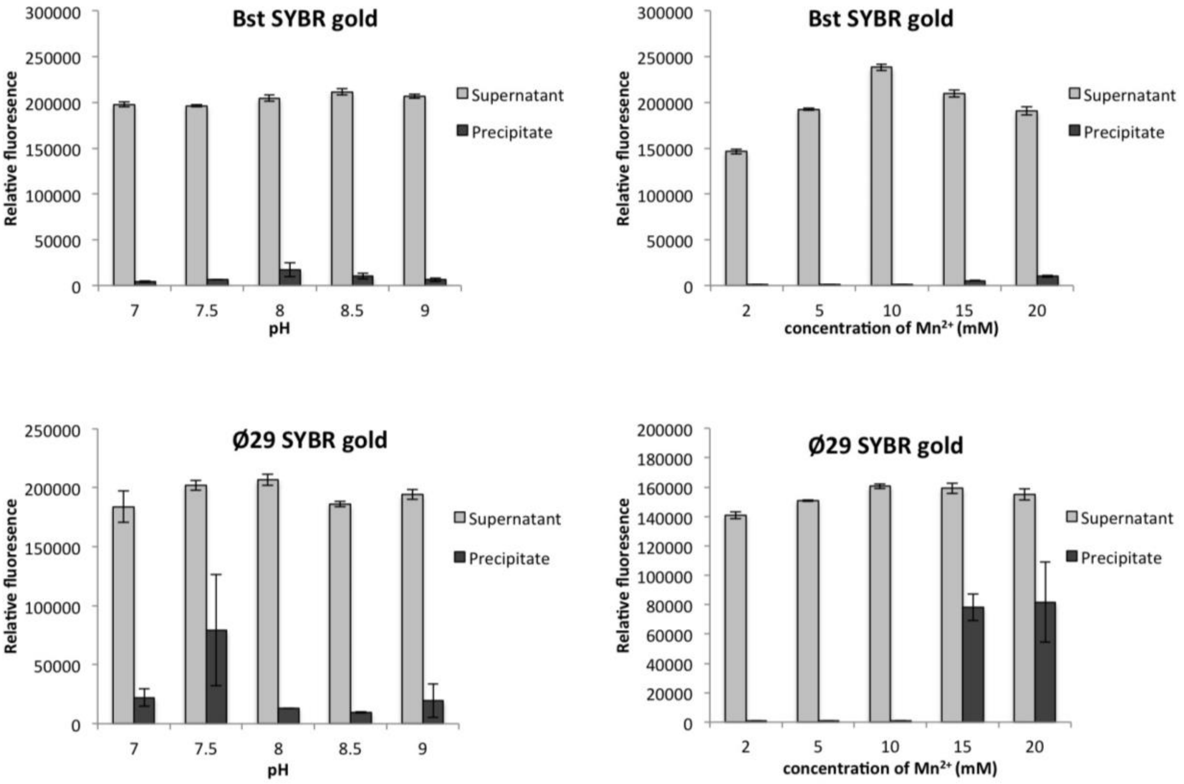

Figure S26. Fluorescence-based analysis comparing the levels of free DNA and DNA incorporated into the nanoconstructs as a function of enzyme, pH, and Mn^{2+} concentration. Samples were broken down using TBE to release DNA prior to incubation with SYBR each dye. A plate reader was then used to compare the DNA levels. The effect of pH on Φ29 RCA was compared at 15 mM $MgSO_4$ and the effect of concentration was evaluated at pH 7.5. The effect of pH on Bst was determined at 15 mM $MgSO_4$ and the effect of concentration was evaluated at pH 8.5.

Agarose gel analysis for $CoCl_2$ screening experiments

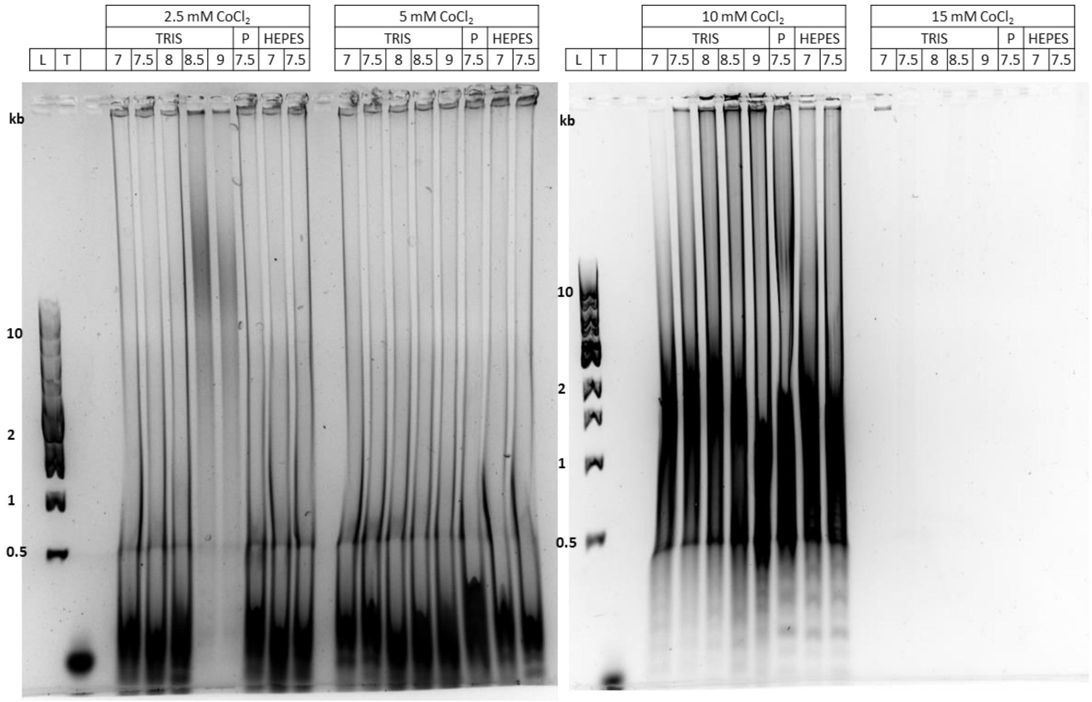

Figure S27. Agarose gel electrophoresis analysis of supernatants from Bst catalysed RCA with $CoCl_2$ as the cofactor. L) 1 kb DNA ladder; T) template T1; P) PIPES; numbers above lanes indicate the pH of the buffer used. These gels show how the buffer used, the concentration of $CoCl_2$ and the pH of the amplification step affect Bst efficiency and processivity. 15 mM of $CoCl_2$ was found to inhibit Bst RCA.

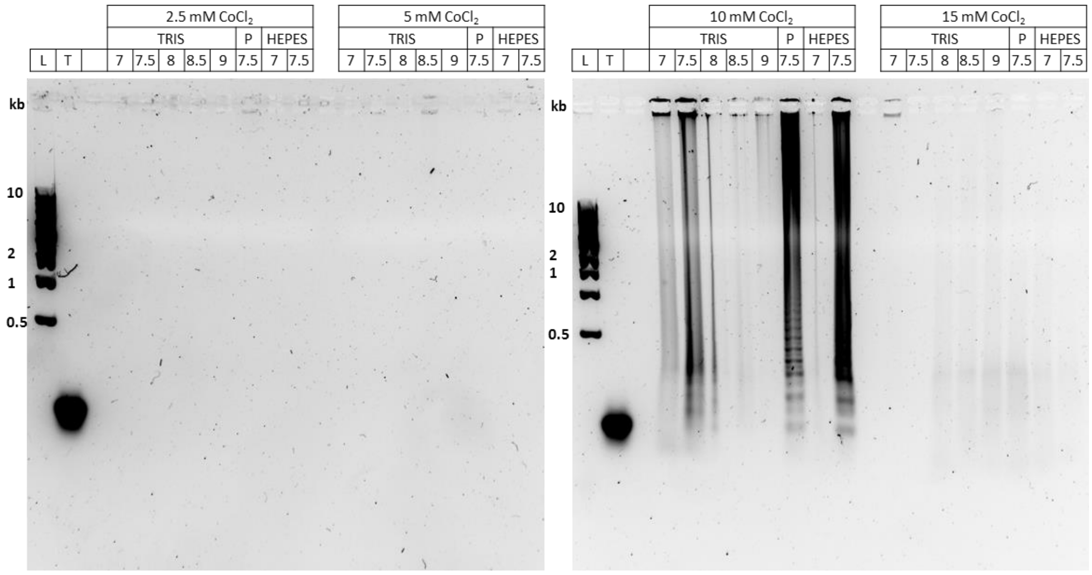

Figure S28. Agarose gel electrophoresis analysis of the precipitates that form during Bst catalysed RCA with $CoCl_2$ as the cofactor. L) 1 kb DNA ladder; T) template T1; P) PIPES; numbers above lanes indicate the pH of the buffer used. These gels show how the buffer used, the concentration of $CoCl_2$ and the pH of the amplification step control the DNA loading of the precipitates formed. Samples with DNA present were then analysed by SEM. 10 mM $CoCl_2$ at pH 7-7.5 resulted in the best DNA incorporation into precipitate.

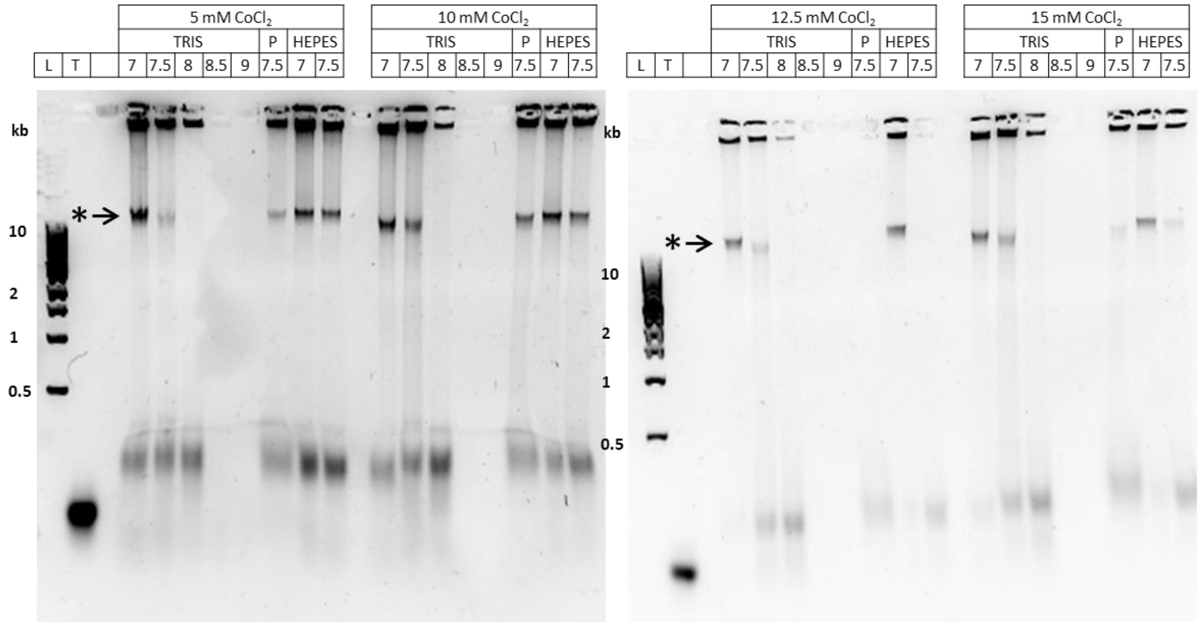

Figure S29. Agarose gel electrophoresis analysis of supernatants from Φ29 catalysed RCA with $CoCl_2$ as the cofactor. L) 1 kb DNA ladder; T) template T1; P) PIPES; *) the appearance of a second band matches with previous literature, suggesting that this is a double stranded product.(3) Numbers above lanes indicate the pH of the buffer used. These gels show how the buffer used, the concentration of $CoCl_2$ and the pH of the amplification step affect Φ29 efficiency and processivity. Overall, RCA was best between pH 7-7.5.

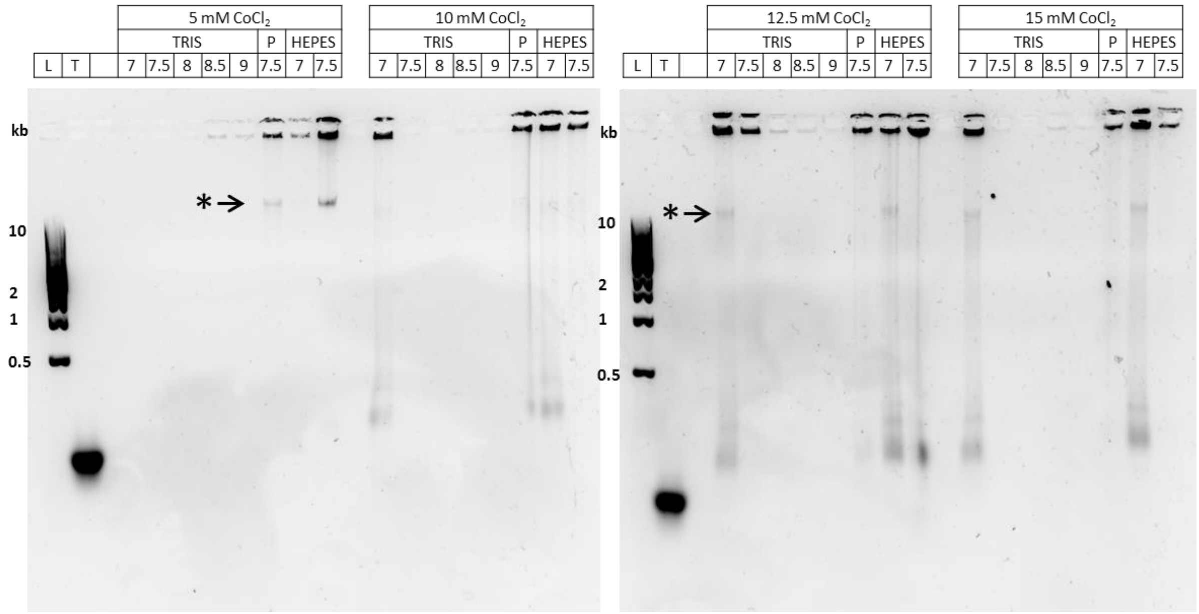

Figure S30. Agarose gel electrophoresis analysis of precipitates that form during Φ29 catalysed RCA with $CoCl_2$ as the cofactor. L) 1 kb DNA ladder; T) template T1; P) PIPES; *) the appearance of a second band matches with previous literature, suggesting that this is a double stranded product.(3) Numbers above lanes indicate the pH of the buffer used. These gels show how the buffer used, the concentration of $CoCl_2$ and the pH of the amplification step control the DNA loading of the precipitates formed. Samples with DNA present were then analysed by SEM. pH 7 was best for DNA incorporation into the precipitates (with the exception of 5 mM $CoCl_2$).

Supplementary CoDNF SEM images

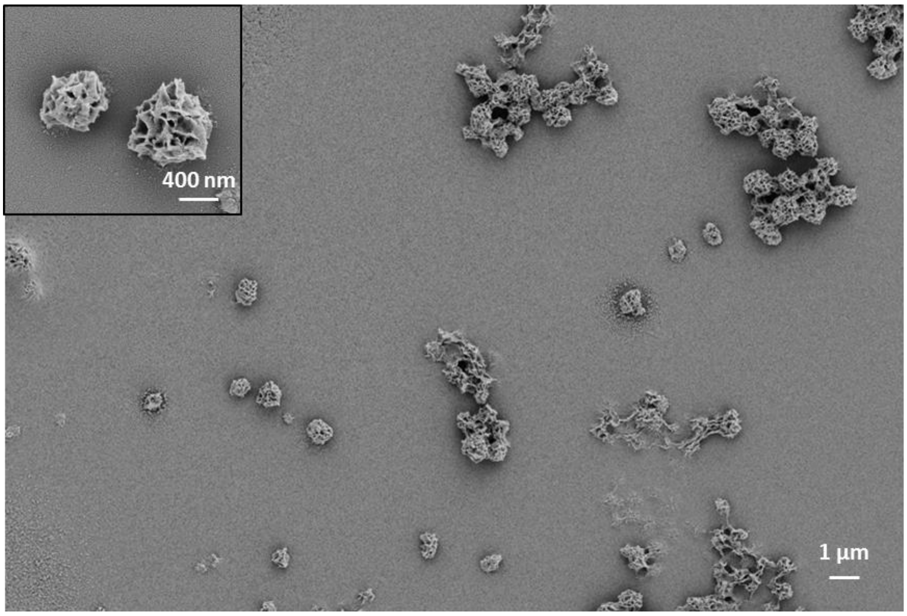

Figure S31. SEM image used to determine the size distribution of the precipitate formed during Bst catalysed RCA in the presence of 10 mM $CoCl_2$. ImageJ was used to measure the diameter of the particles: 0.79 ± 0.13 µm, n = 52.

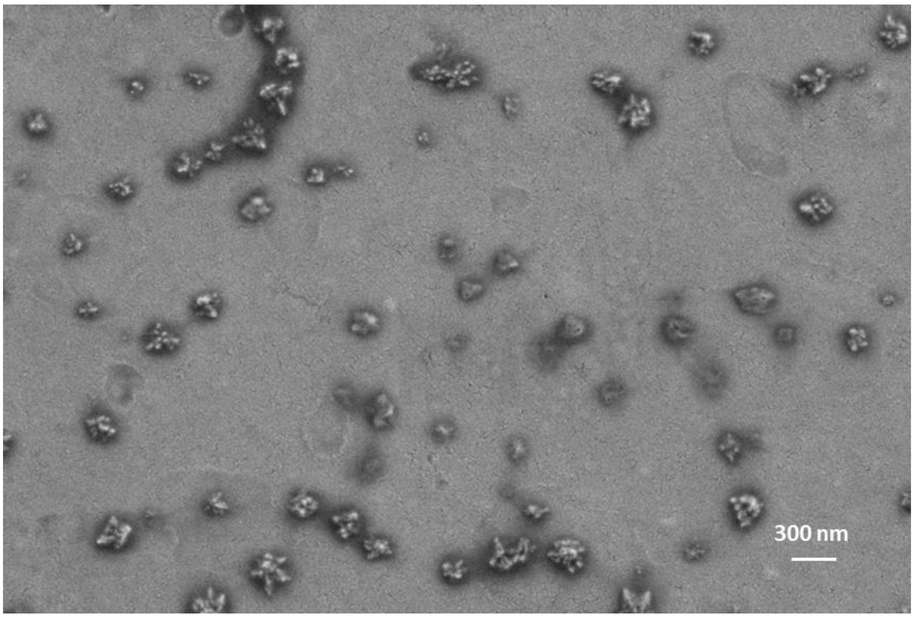

Figure S32. SEM image used to determine the size distribution of the precipitate formed during 0.02 mL scale Bst catalysed RCA in the presence of 15 mM $CoCl_2$. ImageJ was used to measure the average diameter of the particles: 210 ± 43 nm; n = 141.

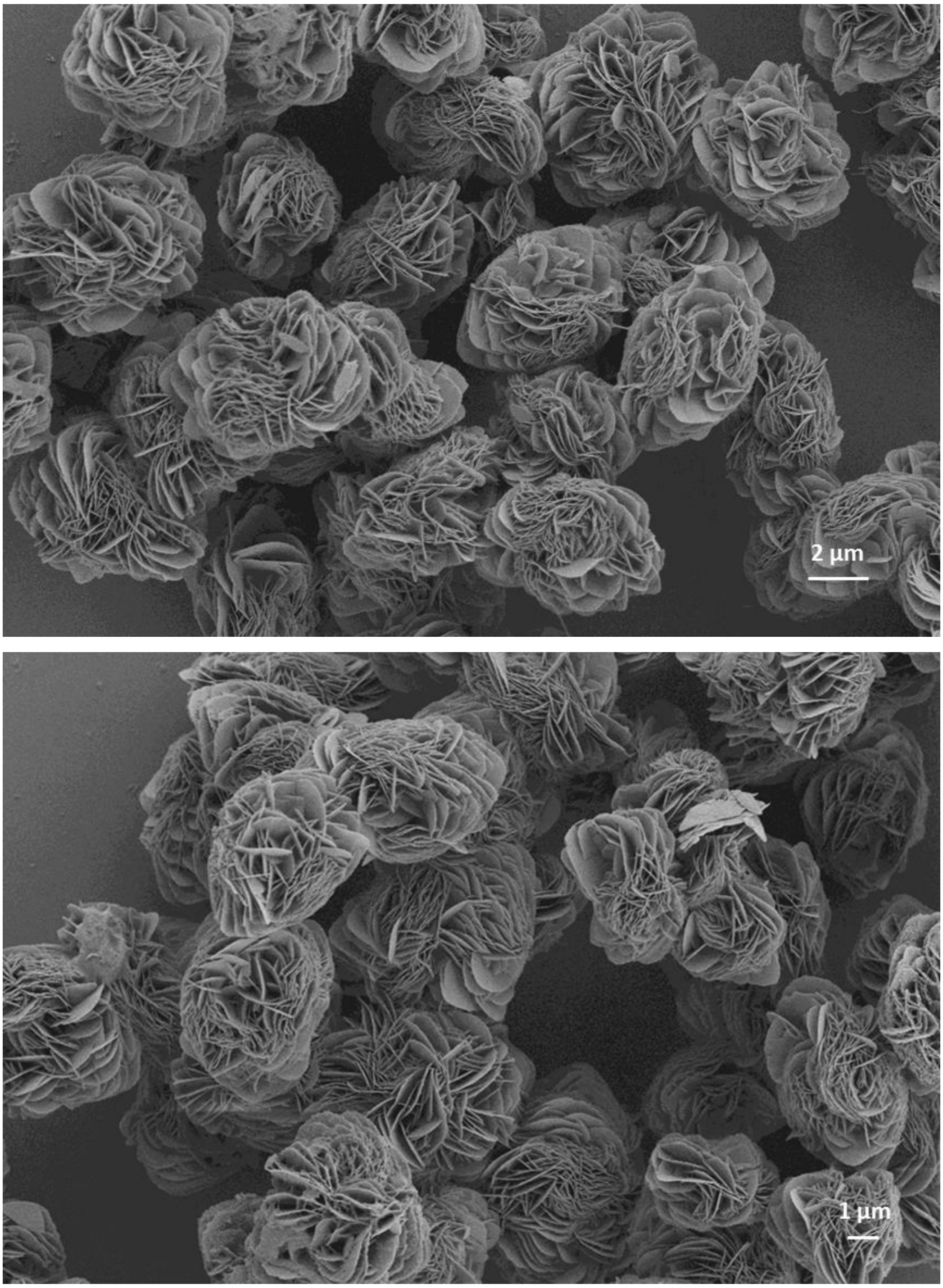

Figure S33. SEM images used to determine the size distribution of the precipitate formed during 1 mL scale Bst catalysed RCA in the presence of 15 mM $CoCl_2$. ImageJ was used to measure the average diameter of the particles: 6.1 ± 0.7 µm, *n* = 26.

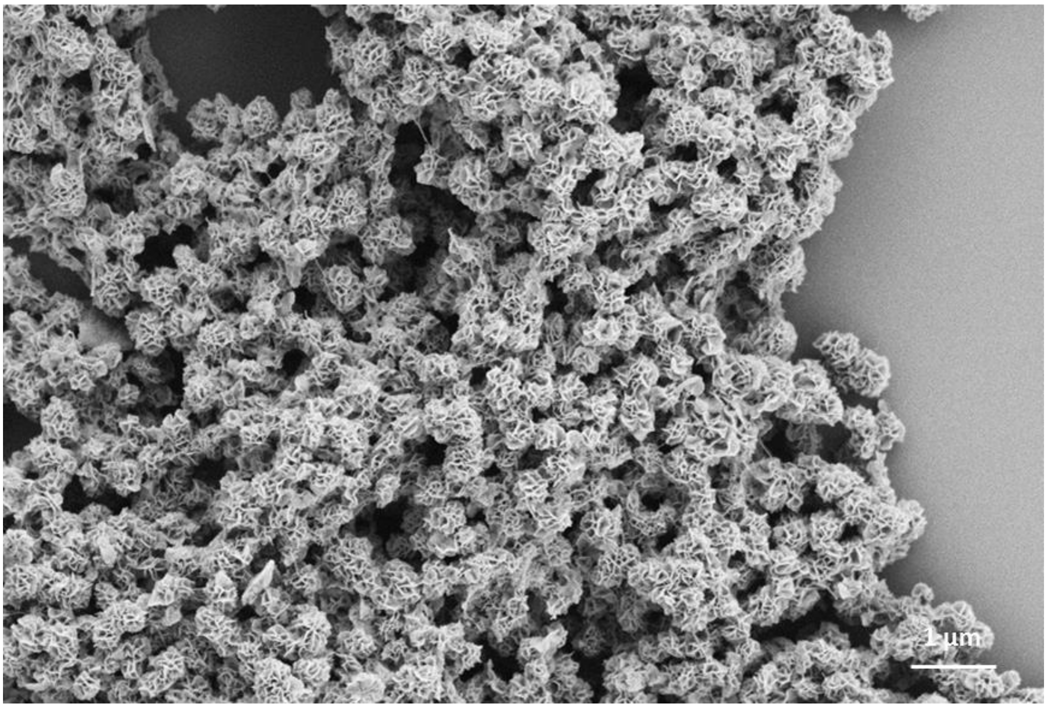

Figure S34. SEM image used to determine the size distribution of the precipitate formed during Φ29 catalysed RCA in the presence of 10 mM $CoCl_2$. ImageJ was used to measure the average diameter of the particles: 446 ± 65 nm, *n* = 108.

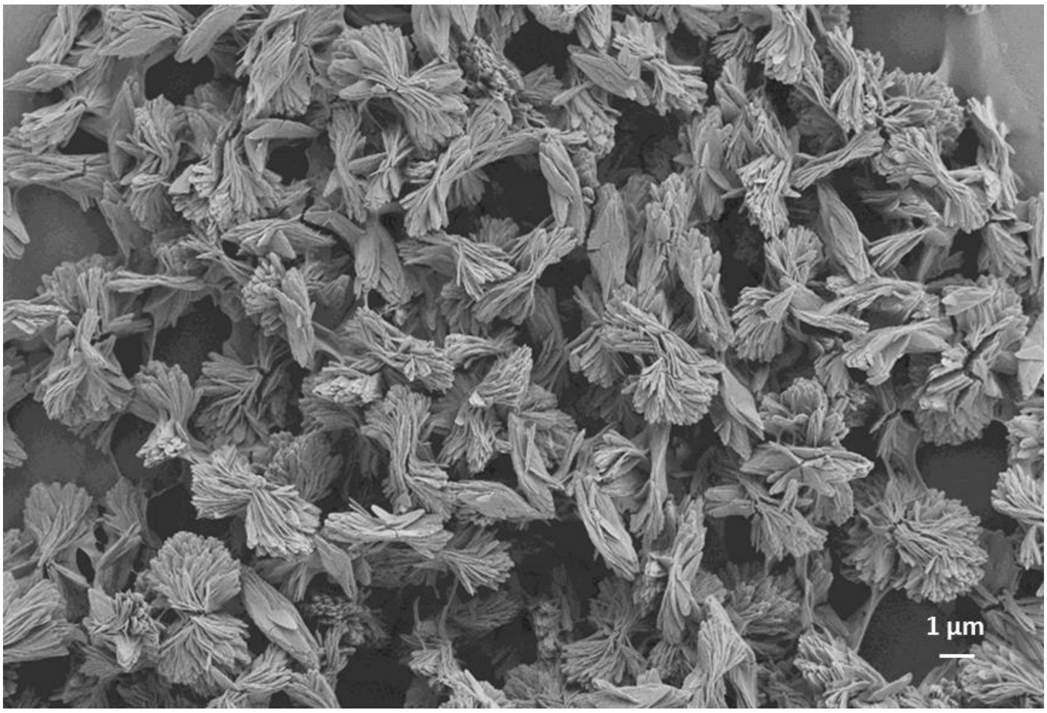

Figure S35. SEM image used to determine the size distribution of the precipitate formed during Φ29 catalysed RCA in the presence of 15 mM $CoCl_2$. ImageJ was used to measure the end-to-end length of the particles: 3.8 ± 0.4 µm; *n* = 31.

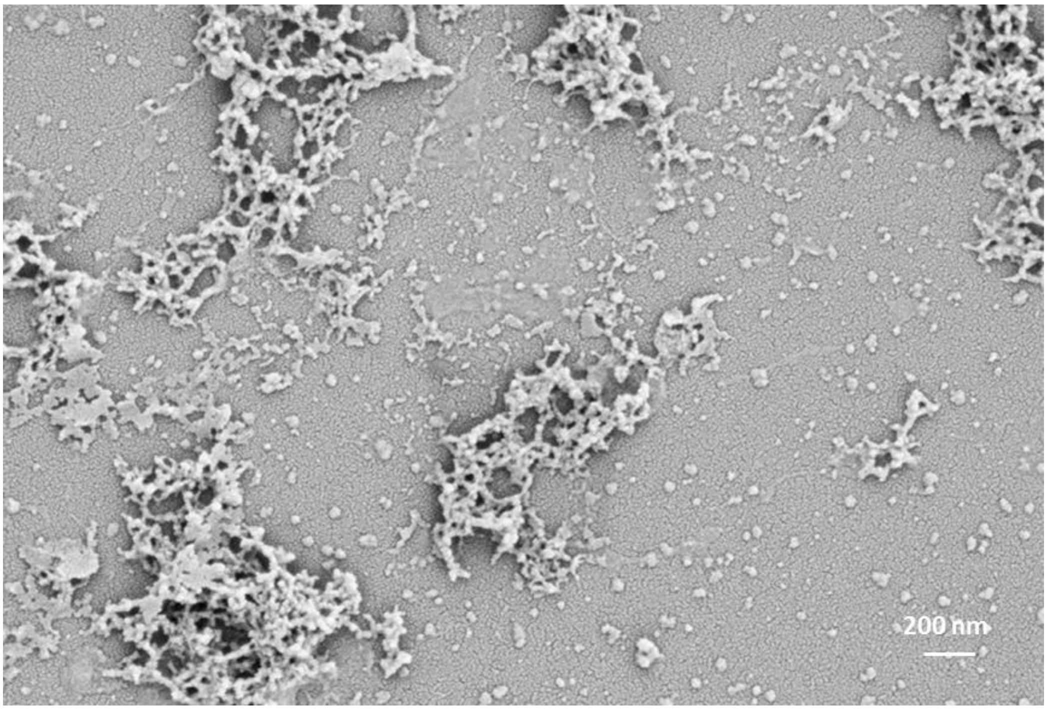

Figure S36. SEM image used to determine the size distribution of the precipitate formed during Φ29 catalysed RCA in the presence of 15 mM $CoCl_2$. ImageJ was used to measure the average diameter of the particles: 30 ± 8 nm, *n* = 176.

SYBR green I and SYBR green II analysis of DNA levels in CoDNFs

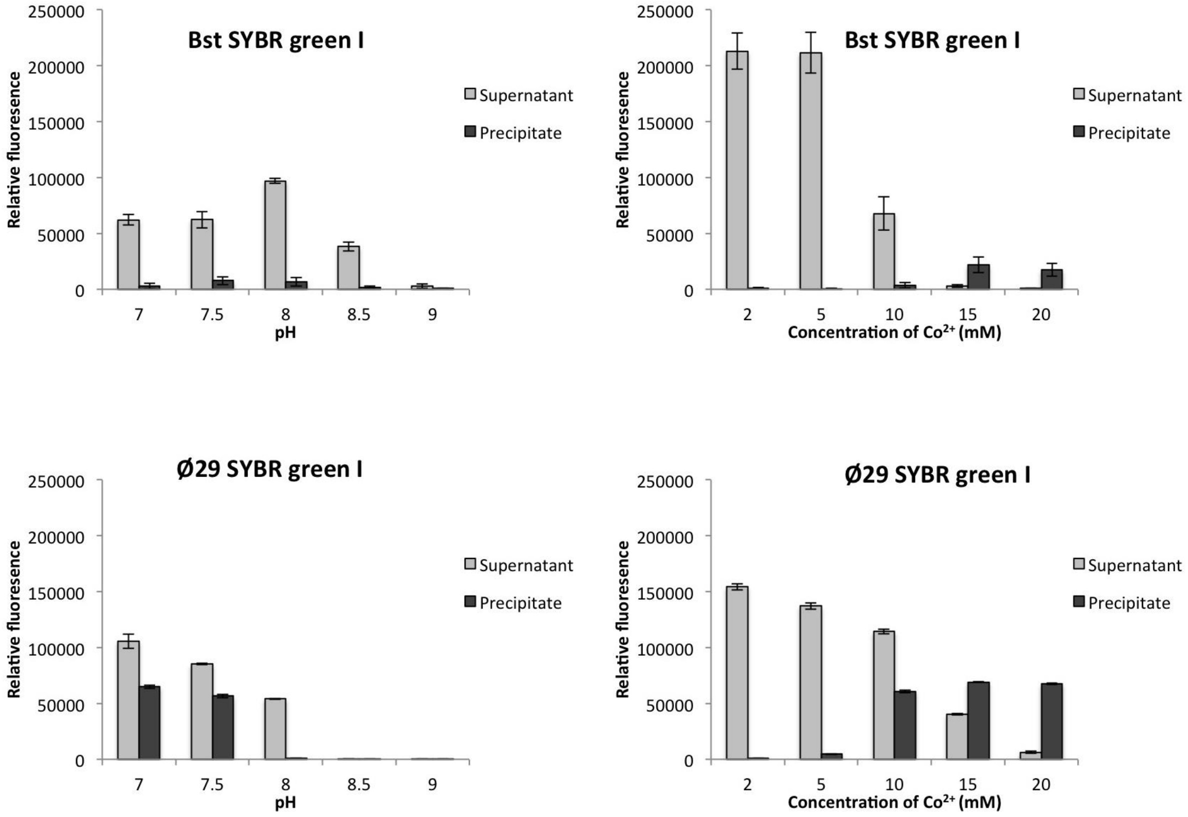

Figure S37. Fluorescence-based analysis comparing the levels of free DNA and DNA incorporated into the nanoconstructs as a function of enzyme, pH, and Co^{2+} concentration. Samples were broken down using TBE to release DNA prior to incubation with SYBR green I. A plate reader was then used to compare the DNA levels. The effect of pH on Φ29 and Bst RCA was compared at 10 mM $CoCl_2$ and the effect of concentration was evaluated at pH 7.0.

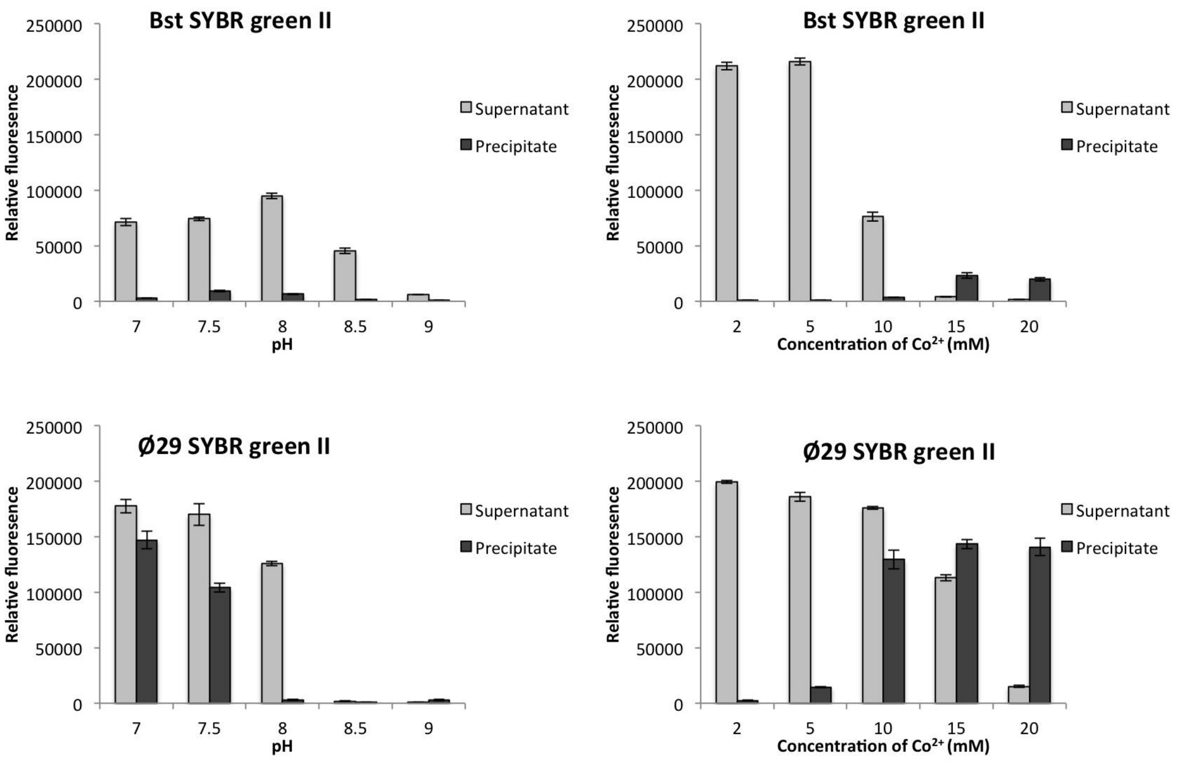

Figure S38. Fluorescence-based analysis comparing the levels of free DNA and DNA incorporated into the nanoconstructs as a function of enzyme, pH, and Co^{2+} concentration. Samples were broken down using TBE to release DNA prior to incubation with SYBR green II. A plate reader was then used to compare the DNA levels. The effect of pH on Φ29 and Bst RCA was compared at 10 mM $CoCl_2$ and the effect of concentration was evaluated at pH 7.0.

SEM images of particles formed using T2 and S2

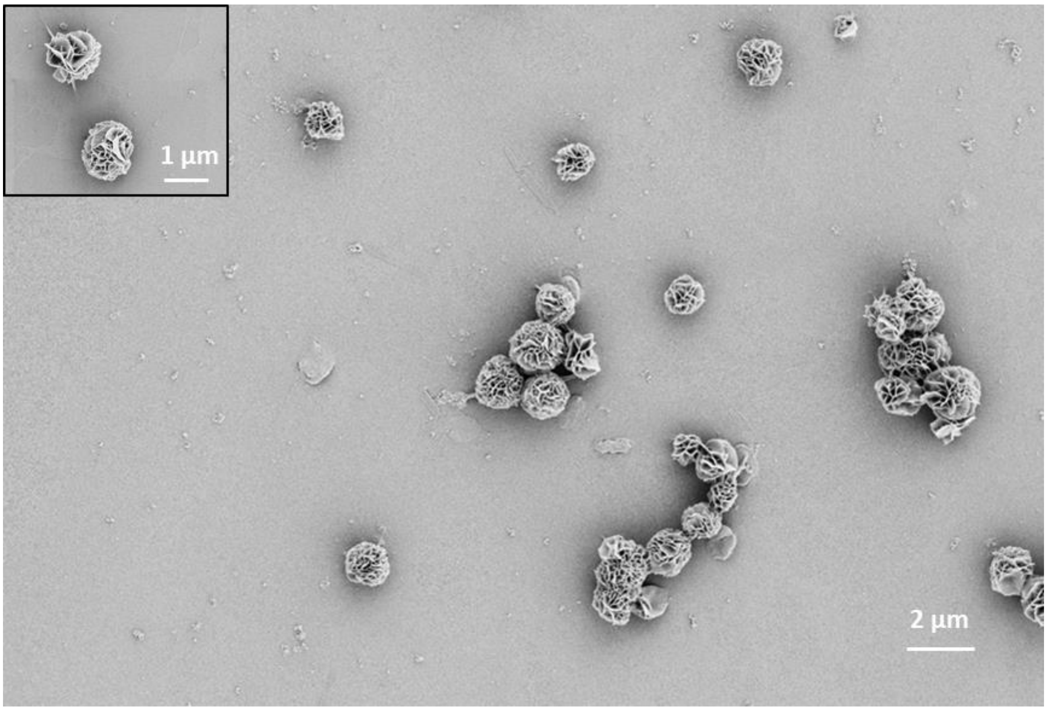

Figure S39. SEM image of precipitate formed when using T2 and S2 during Φ29 catalysed RCA with 10 mM $MnCl_2$. This image shows that the morphology and size of the particles are similar to those formed using T1 and S1 (Fig 3 in paper) suggesting that the morphology of the particles is not sequence dependent.

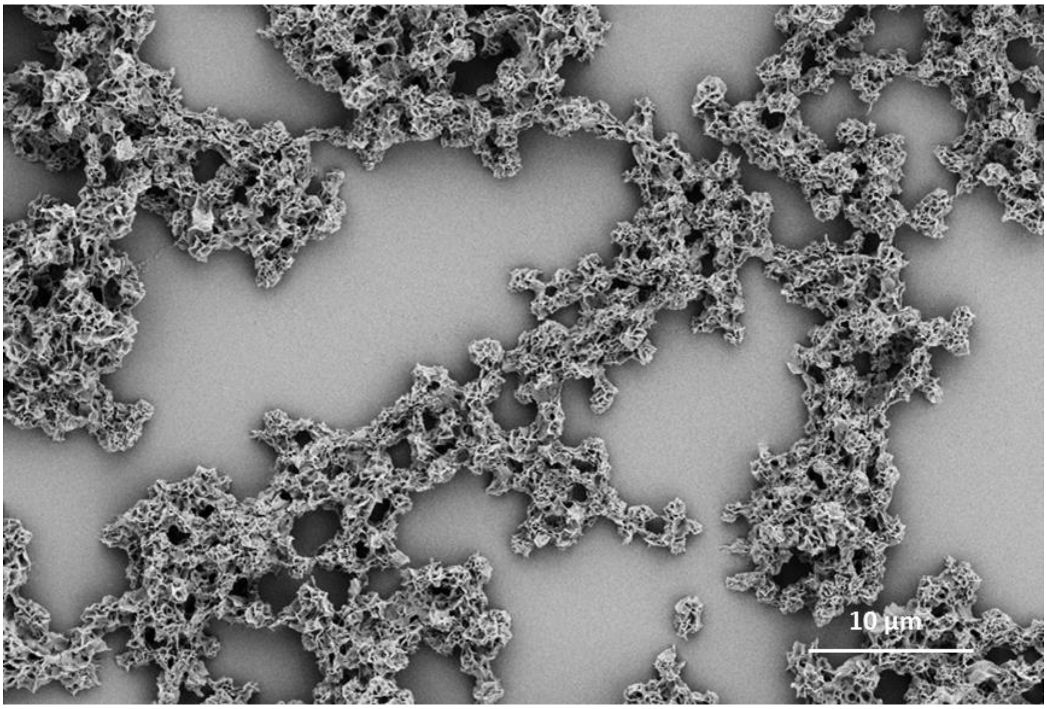

Figure S40. SEM image of precipitate formed when using T2 and S2 during Bst catalysed RCA with 10 mM $MnCl_2$. This image shows that the morphology and size of the particles are similar to those formed using T1 and S1 (Fig 3 in paper) suggesting that the morphology of the particles is not sequence dependent.

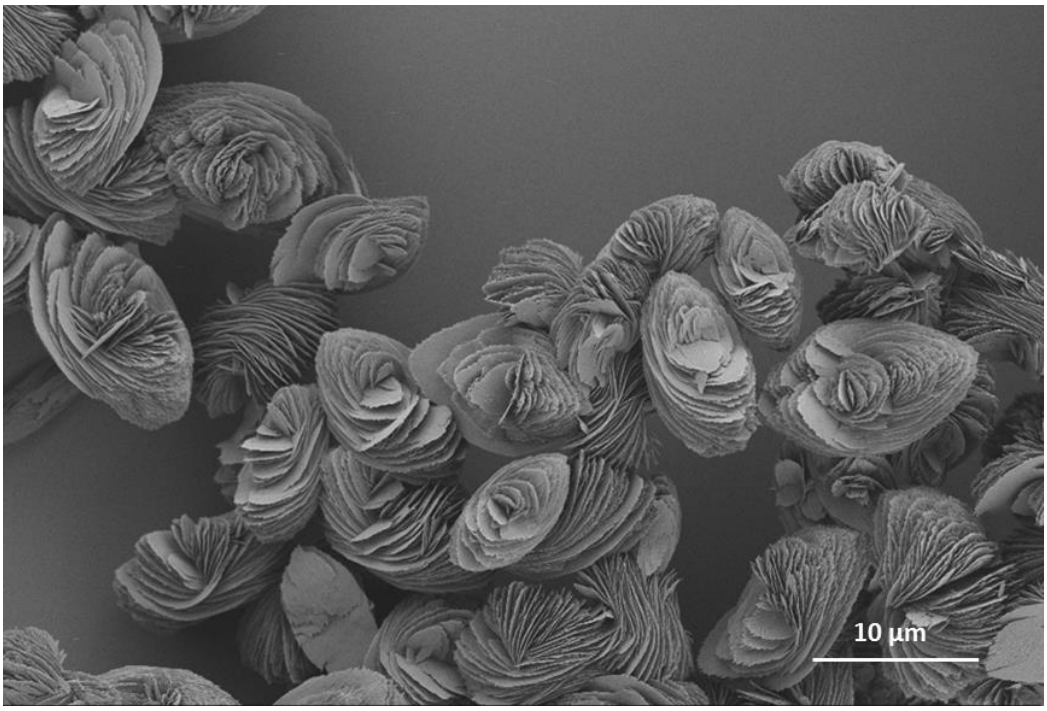

Figure S41. SEM image of precipitate formed when using T2 and S2 during Bst catalysed RCA with 5 mM $MnCl_2$. This image shows that the morphology and size of the particles are similar to those formed using T1 and S1 (Fig 3 in paper) suggesting that the morphology of the particles is not sequence dependent.

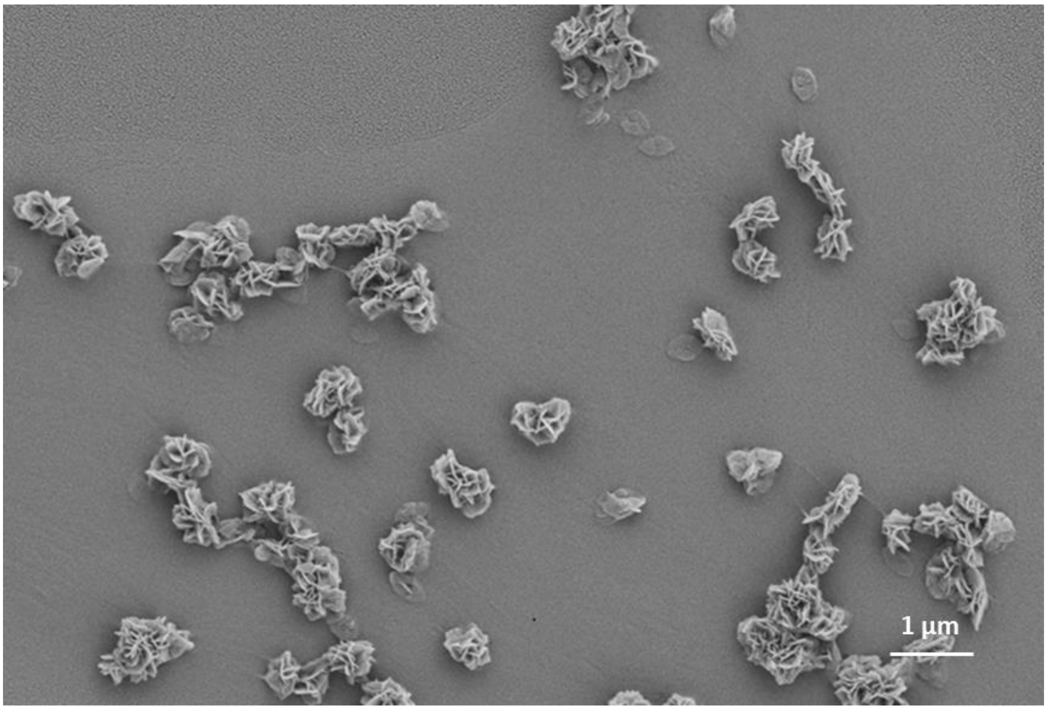

Figure S42. SEM image of the precipitate formed when using T2 and S2 during Φ29 catalysed RCA with 10 mM $CoCl_2$. The incubation time for the RCA step was 30 h for this experiment, which is different to the 20 h incubation time used in the earlier Φ29 RCA reactions described in this paper. This image shows that the morphology and size of the particles are similar to those formed using T1 and S1 (Fig 4 in paper) suggesting that the morphology of the particles is not sequence dependent.

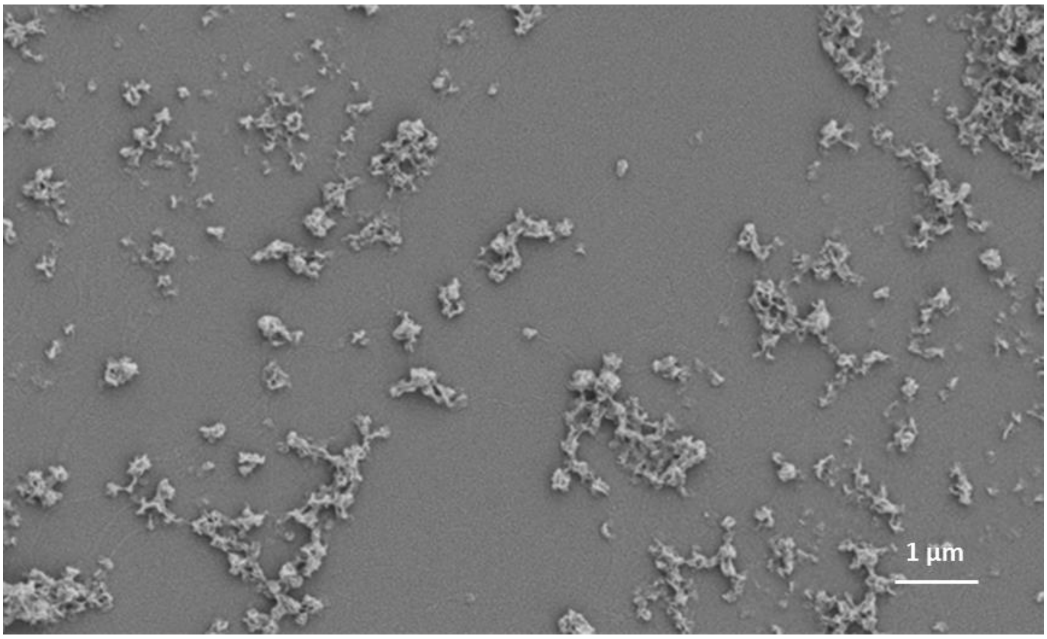

Figure S43. Representative SEM image of Φ29 catalysed RCA in the presence of 15 mM $CoCl_2$ for T2. The incubation time for the RCA step was 30 h, which is different to the 20 h incubation time used in the earlier Φ29 RCA reactions described in this paper. This image shows that the morphology and size of the particles are similar to those formed using T1 and S1 (Fig 4 in paper) suggesting that the morphology of the particles is not sequence dependent.

Additional Energy-dispersive X-ray spectroscopy (EDX) data

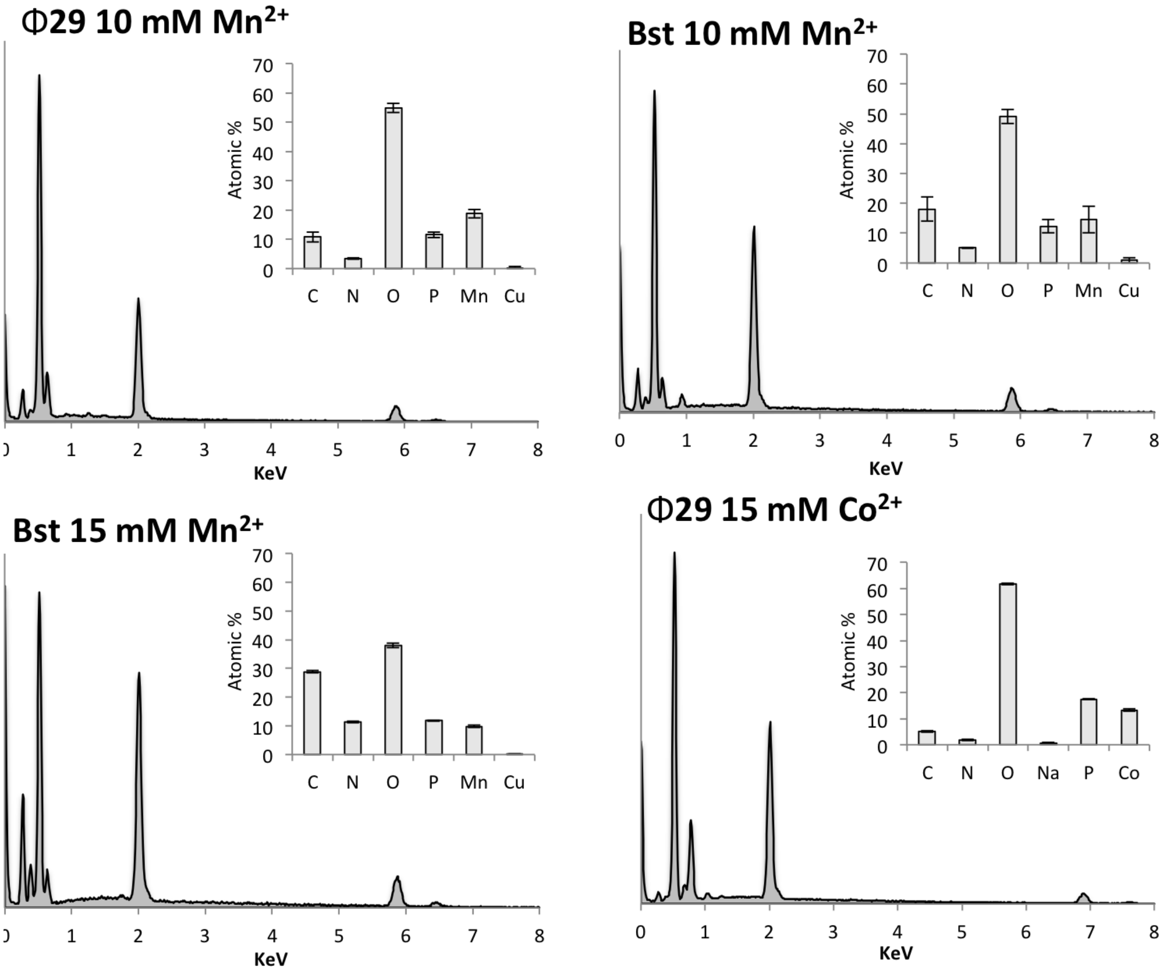

Figure S44. Additional EDX analysis of particles used in subsequent magnetic studies. EDX data is shown for MnDNFs prepared using Φ29 in Tris buffer pH 8.0, MnDNFs prepared using Bst in Tris buffer pH 7.5 and CoDNFs prepared in Tris buffer pH 7.0. The CoDNFs prepared using a 1 mL RCA volume. Samples were analysed on a carbon support film with a copper mesh to minimise signals from mounting which is why high carbon levels and copper are detected.

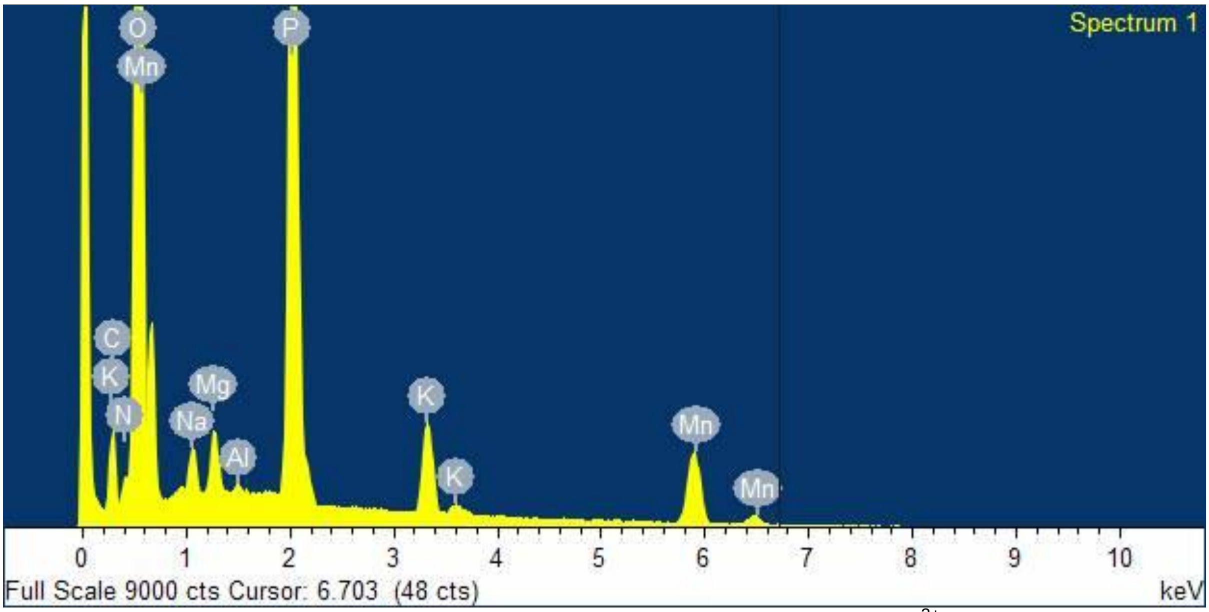

Figure S45. EDX analysis of particles formed during Bst RCA using 5 mM Mn^{2+} in pH 7.0 HEPES buffer.

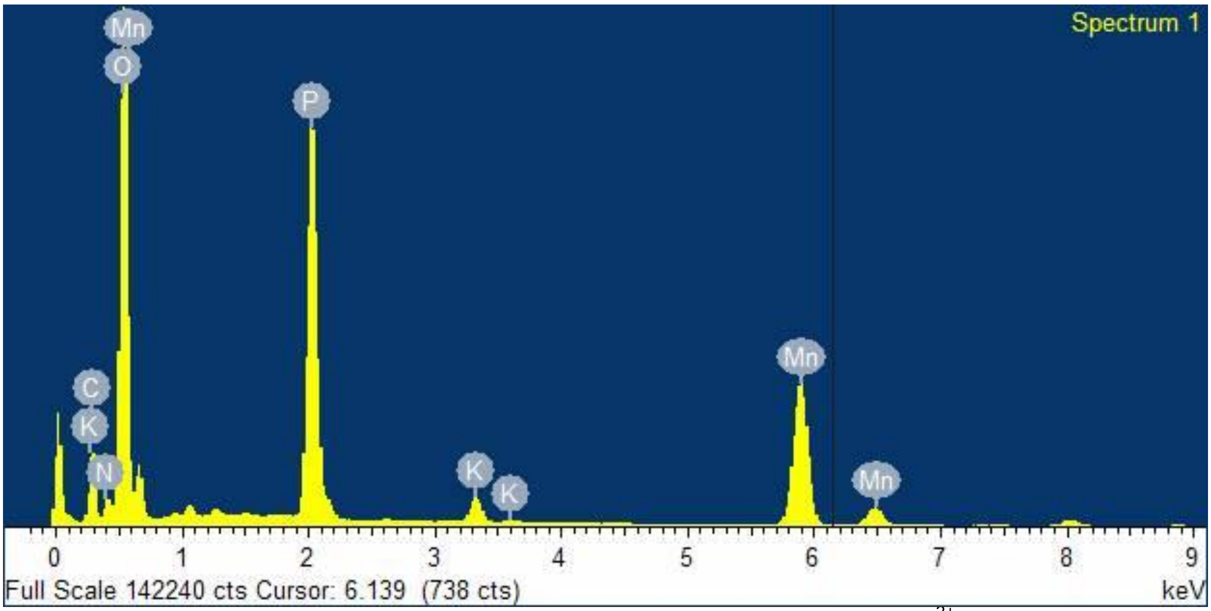

Figure S46. EDX analysis of particles formed during Bst RCA using 10 mM Mn^{2+} in pH 7.0 HEPES buffer.

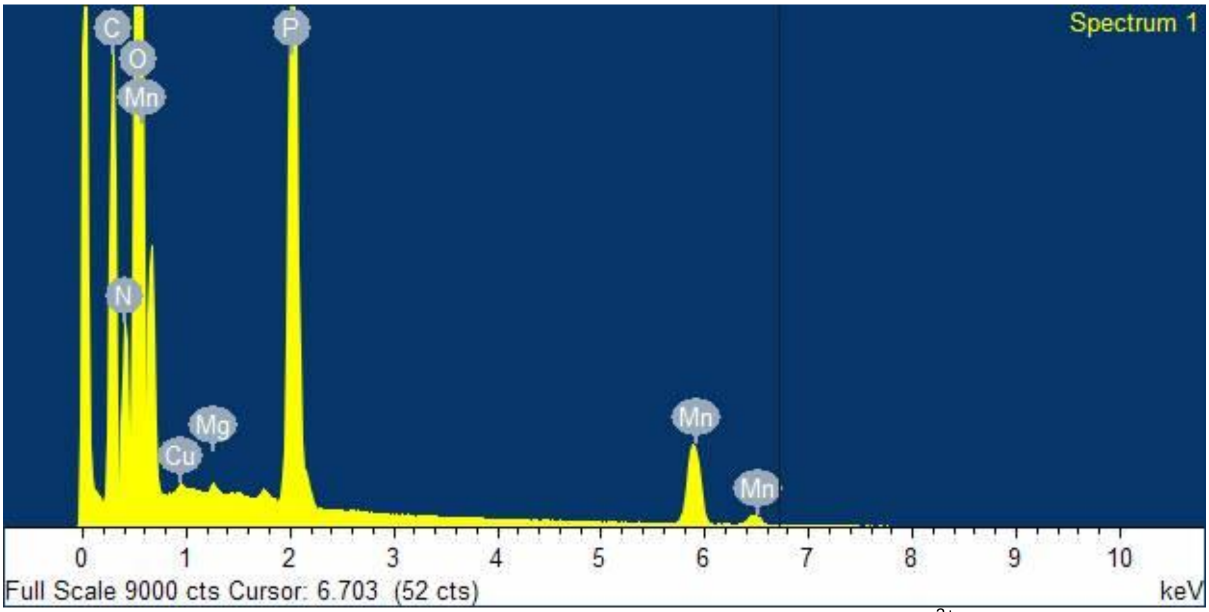

Figure S47. EDX analysis of particles formed during Bst RCA using 15 mM Mn^{2+} in pH 7.0 HEPES buffer.

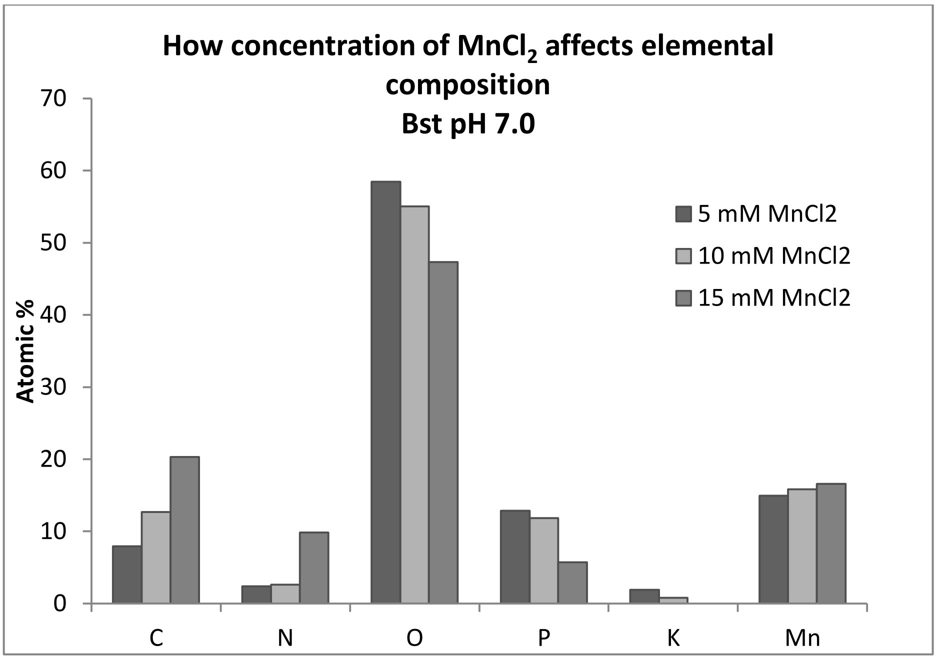

Figure S48. Comparison of how the cation concentration during RCA affects the elemental composition of the resulting particles. These RCA reactions were carried out in HEPES buffer pH 7.0 (Figure S45-47). Elemental composition was determined using EDX. This figure shows that as the concentration of Mn^{2+} is increased in the RCA mixture, the amount of nitrogen present in the particles increases relative to the phosphorus and oxygen levels. This suggests that the DNA loading of the particles is increased at higher cation concentration.

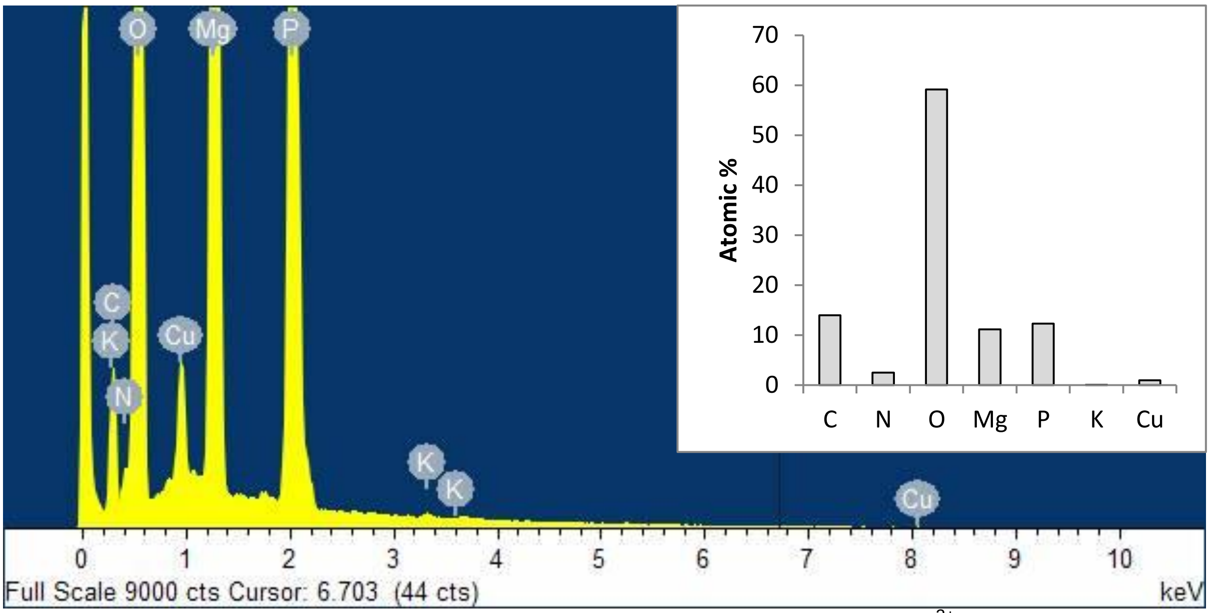

Figure S49. EDX analysis of particles formed during Bst RCA using 15 mM Mg^{2+} in Tris buffer at pH 8.5. Inset: elemental composition of MgDNFs as determined using SEM-based EDX.

Infrared spectra

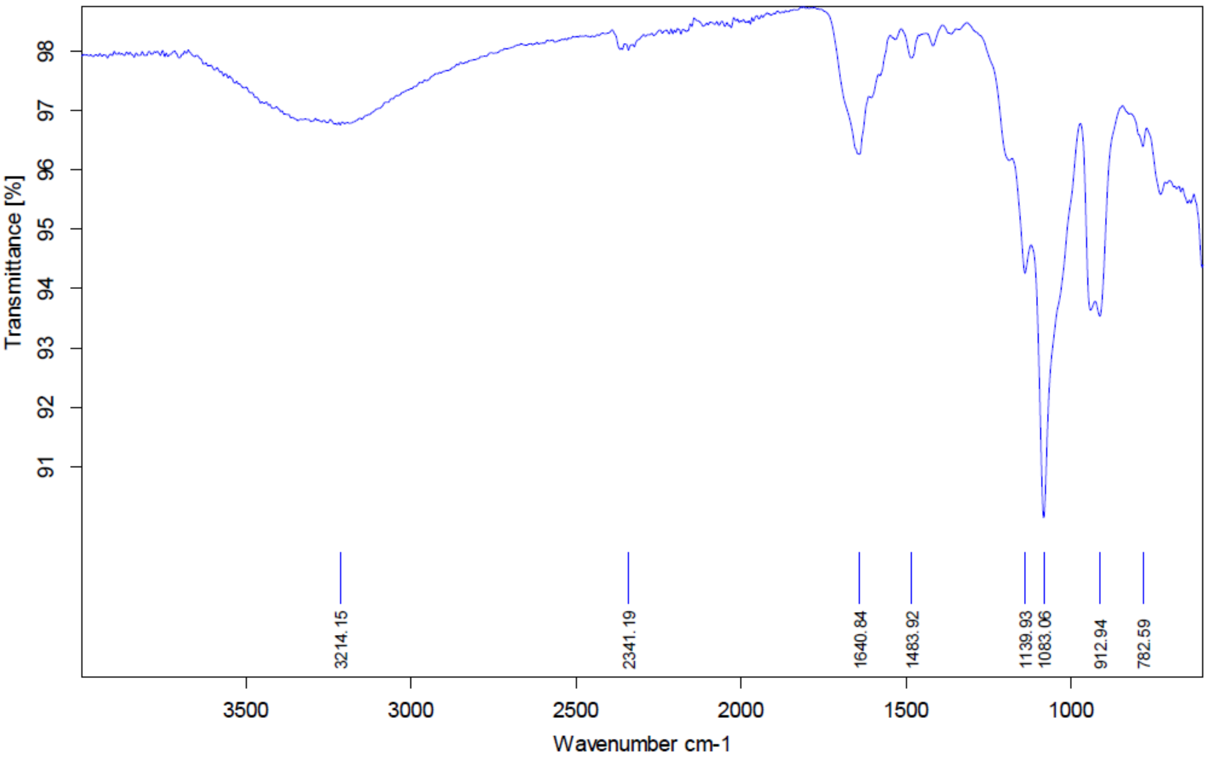

Figure S50. FTIR analysis of MnDNFs. Characteristic stretches for the pyrophosphate are observed in the fingerprint region of the spectra. This shows greater similarity to Fig S54 than Fig S53 supporting the theory that the particles are composed of manganese and pyrophosphate.

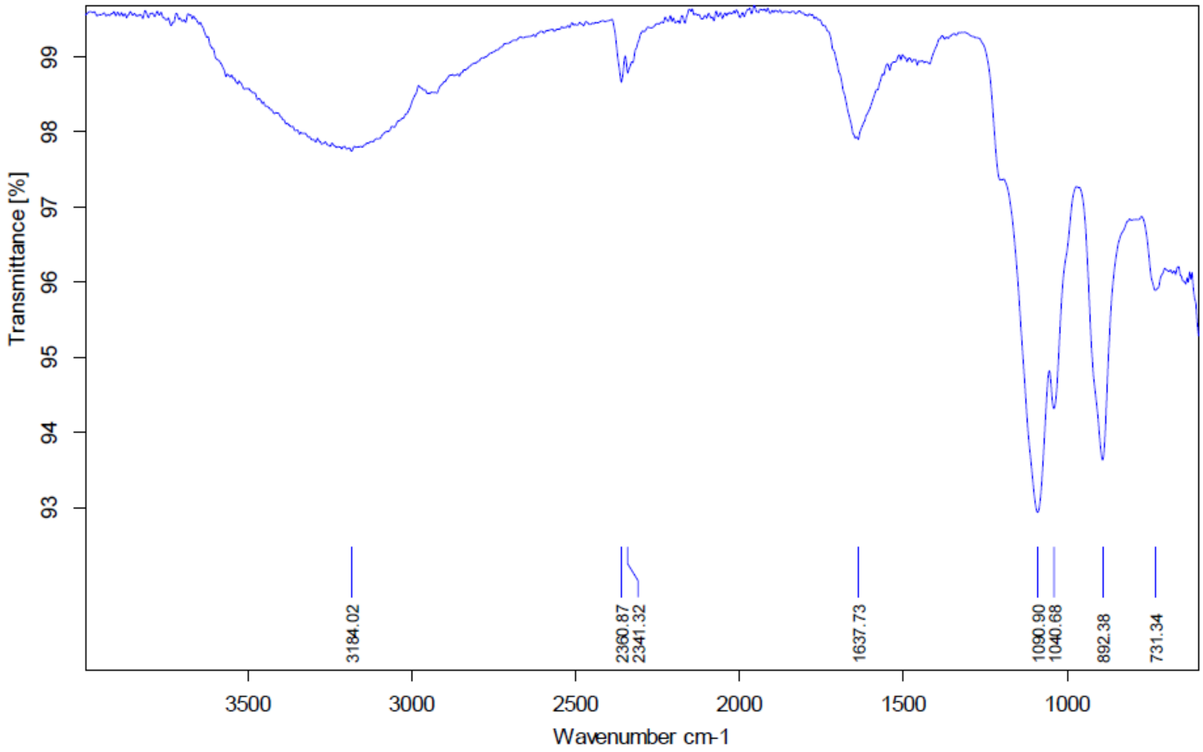

Figure S51. FTIR analysis of CoDNFs. Characteristic stretches for the pyrophosphate are observed in the fingerprint region of the spectra. This shows greater similarity to Fig S56 than Fig S55 supporting the theory that the particles are composed of cobalt and pyrophosphate.

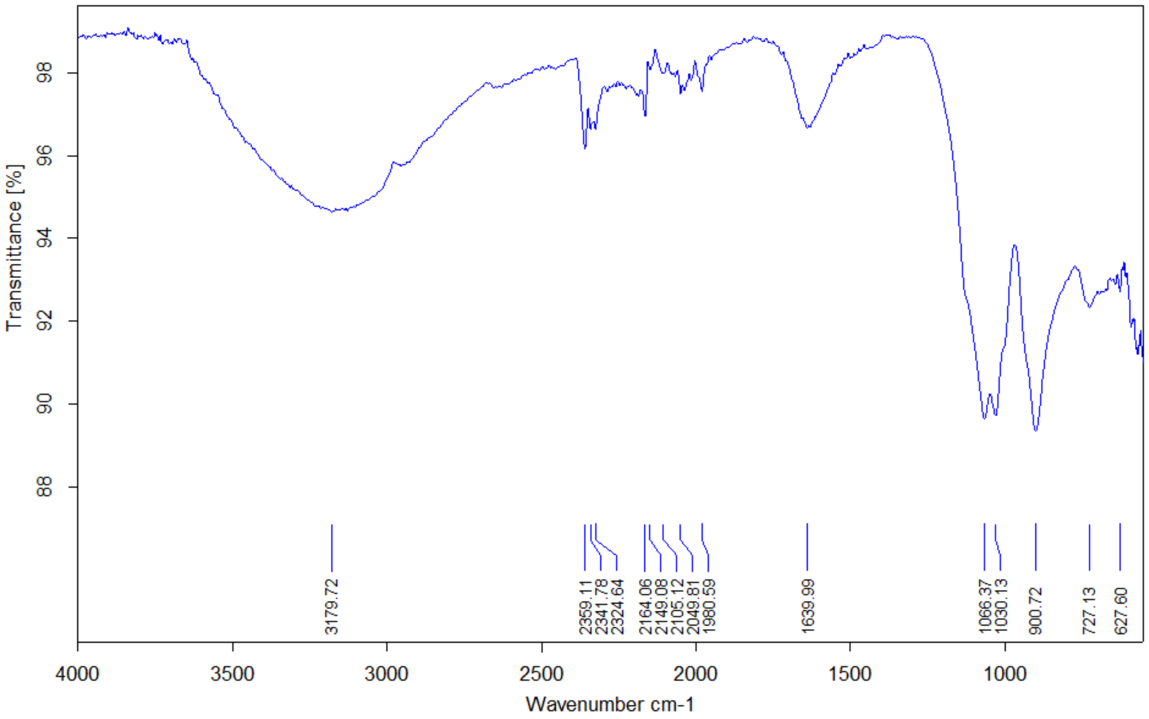

Figure S52. FTIR analysis of ZnDNFs. Characteristic stretches for the pyrophosphate are observed in the fingerprint region of the spectra. This shows greater similarity to Fig S58 than Fig S57 supporting the theory that the particles are composed of cobalt and pyrophosphate.

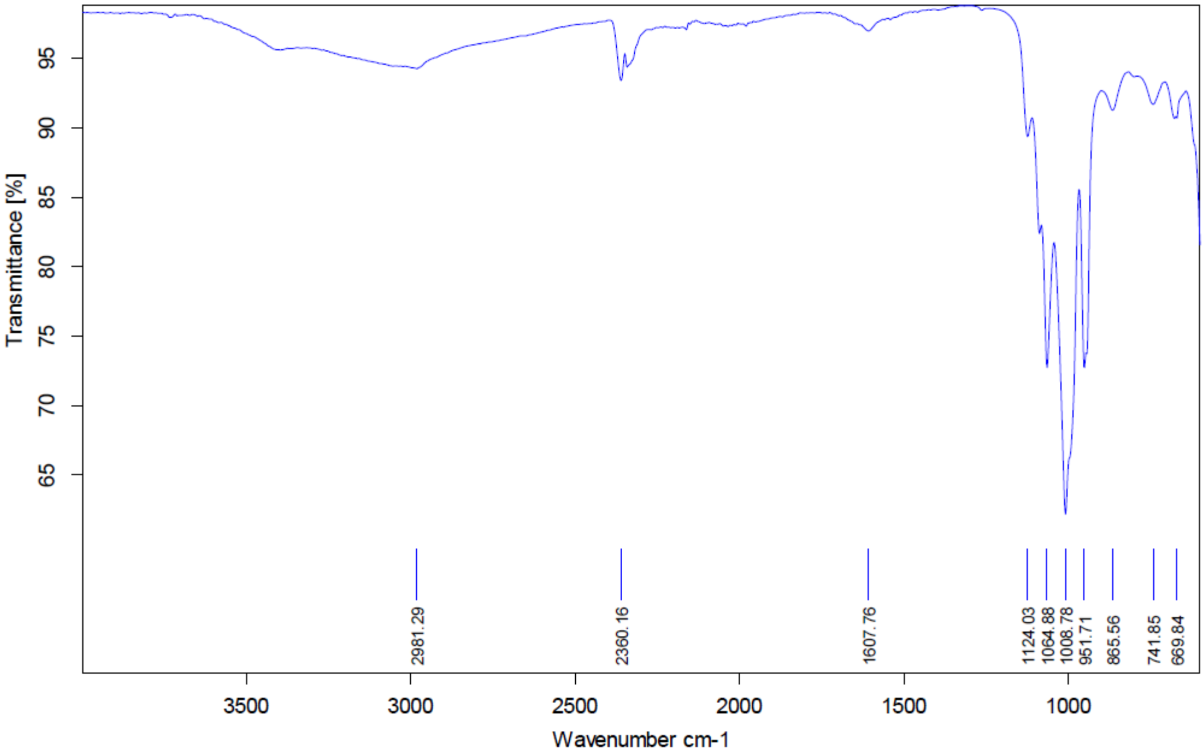

Figure S53. FTIR analysis of precipitate formed when KH_2PO_4 in HEPES buffer at pH 7.5 is mixed with $MnCl_2$.

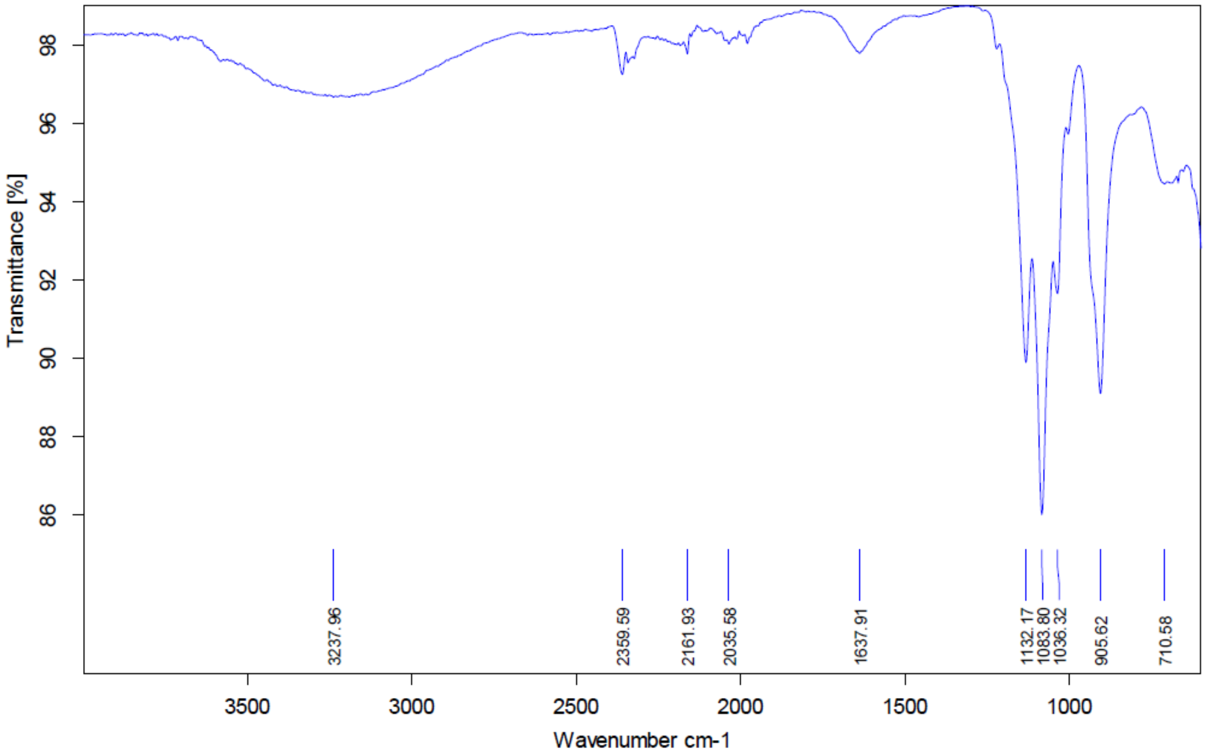

Figure S54. FTIR analysis of precipitate formed when sodium pyrophosphate dibasic in HEPES buffer at pH 7.5 is mixed with $MnCl_2$.

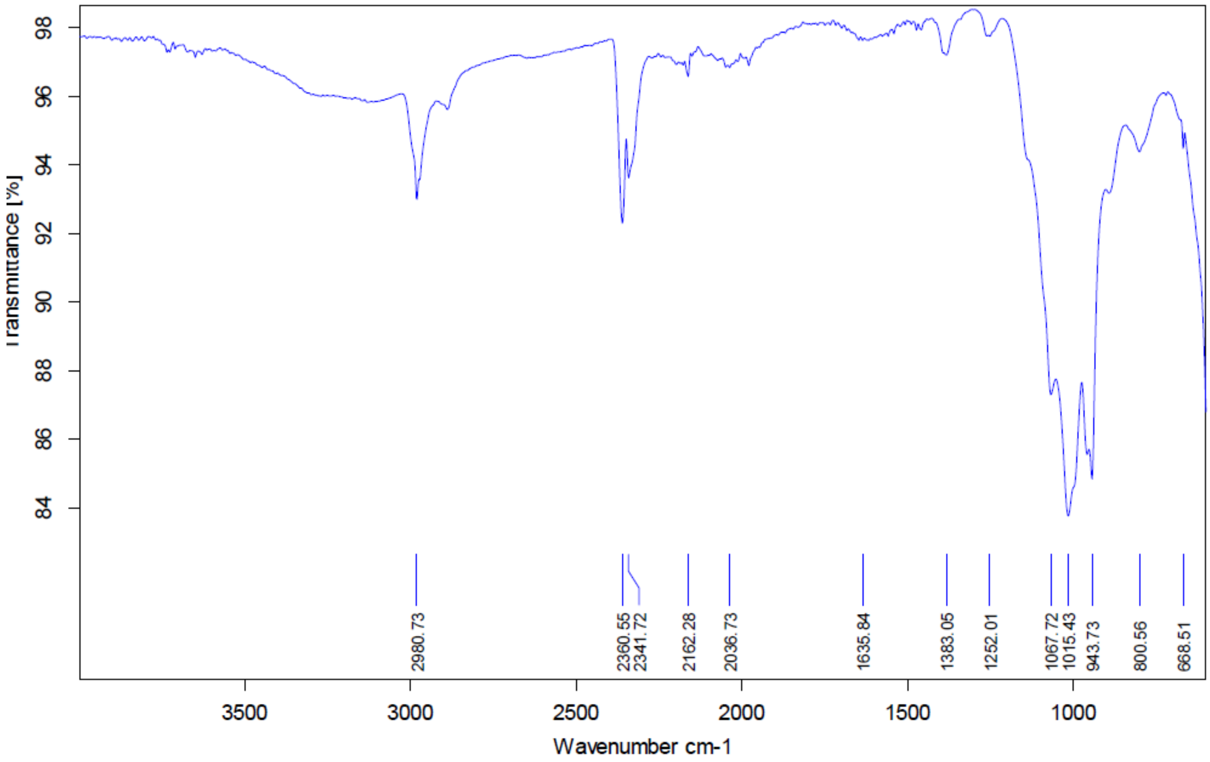

Figure S55. FTIR analysis of precipitate formed when KH_2PO_4 in HEPES buffer at pH 7.5 is mixed with $CoCl_2$.

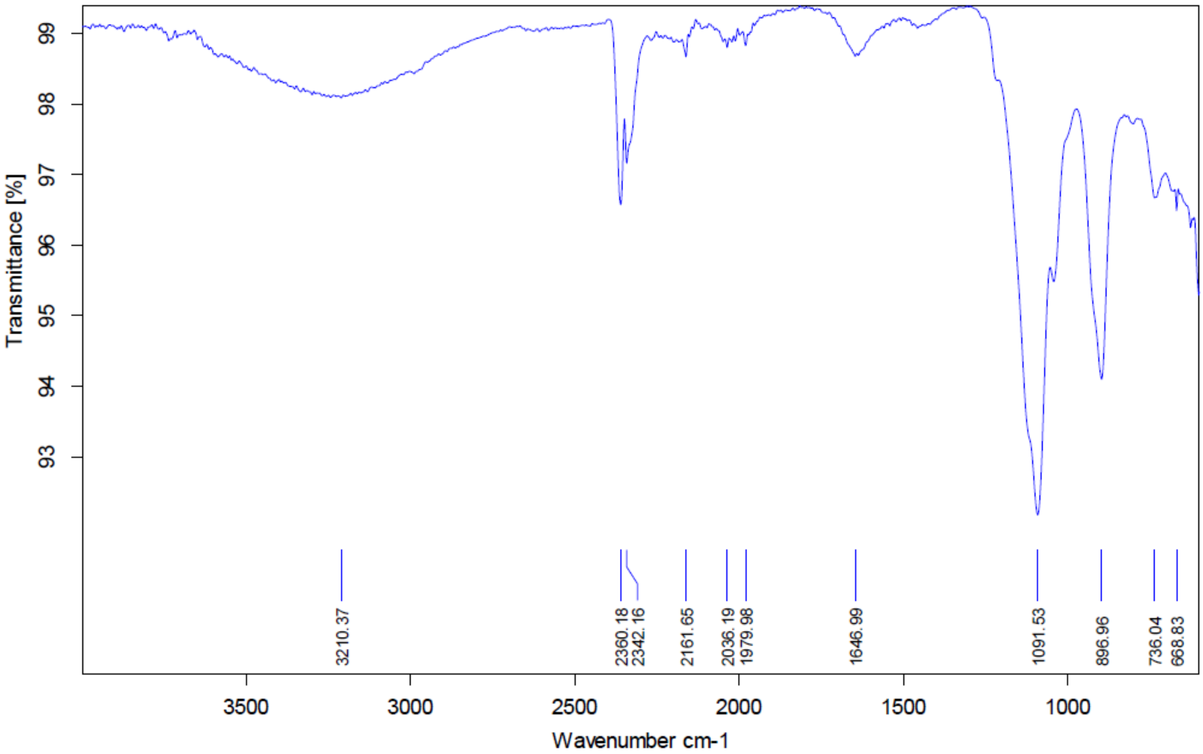

Figure S56. FTIR analysis of precipitate formed when sodium pyrophosphate dibasic in HEPES buffer at pH 7.5 is mixed with $CoCl_2$.

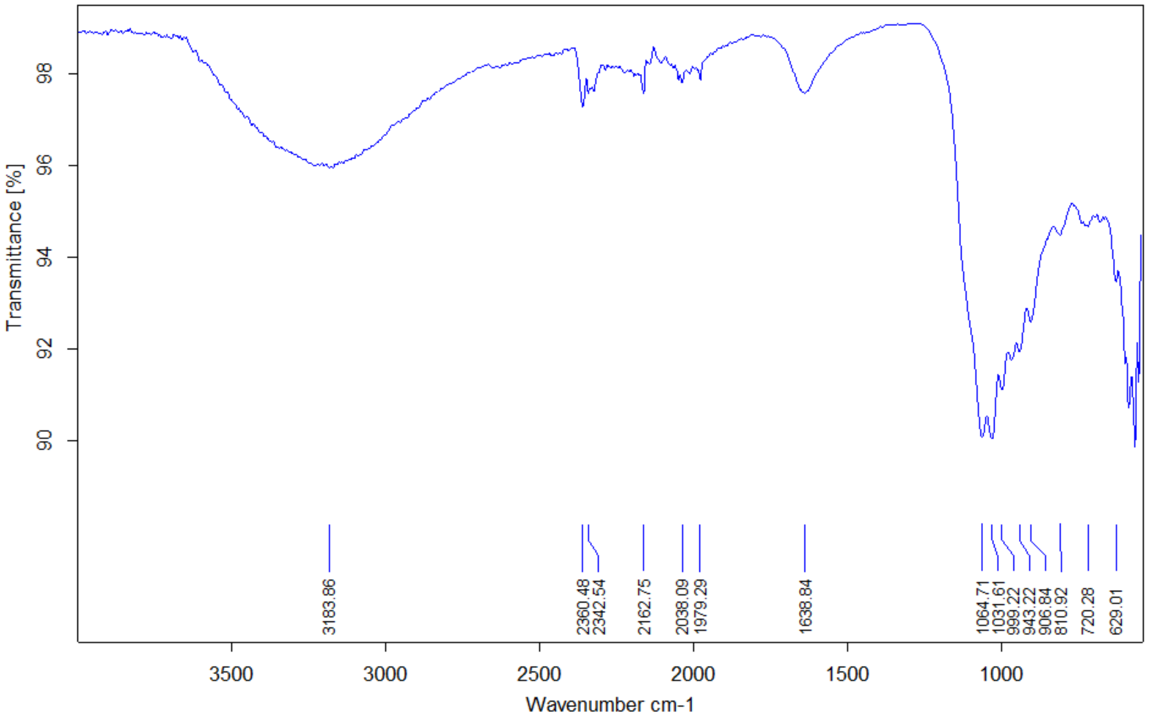

Figure S57. FTIR analysis of precipitate formed when KH_2PO_4 in HEPES buffer at pH 7.5 is mixed with $ZnCl_2$.

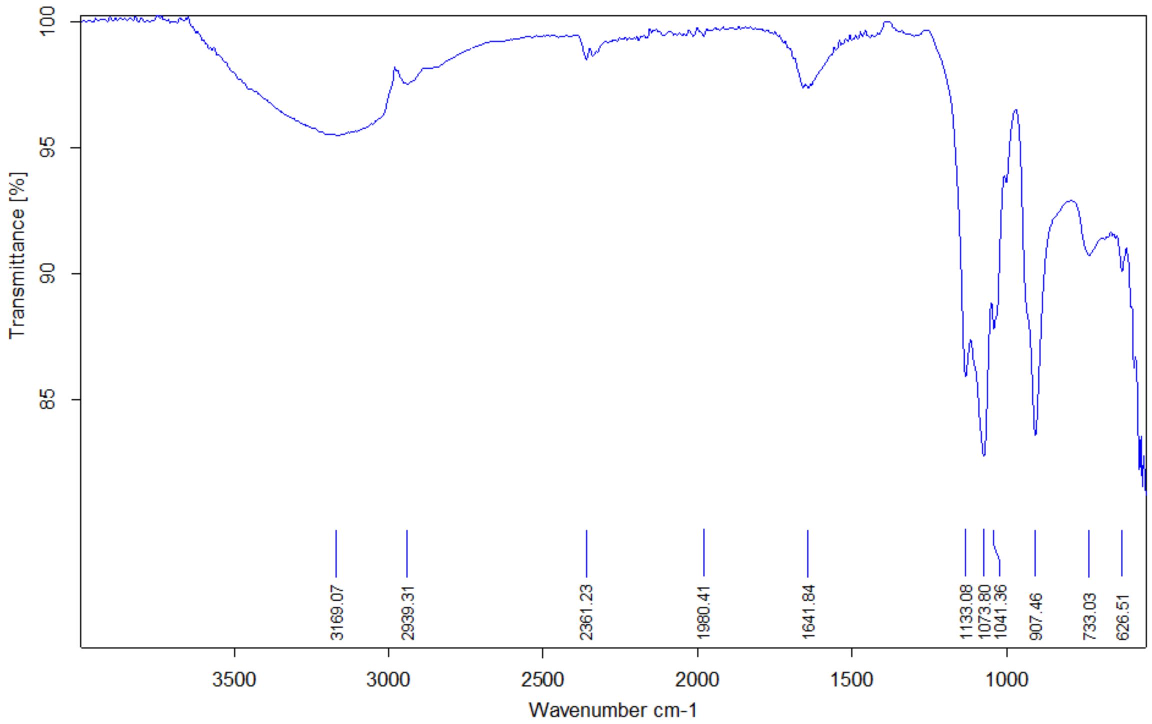

Figure S58. FTIR analysis of precipitate formed when sodium pyrophosphate dibasic in HEPES buffer at pH 7.5 is mixed with $ZnCl_2$.

Surface potential of various DNFs

Cation	pH	cation concentration (mM)	Zeta potential (mV)
Mn^{2+}	7	15	-41.6*
	7.5		-44.7
	8		-10.2
	8.5		-2.5
	9		x
	7	2	x
		5	x
		10	-26.8
		15	-40.5*
		20	-9.6
Co^{2+}	7	10	-11.5
	7.5		-0.3
	8		3.5*
	8.5		x
	9		x
	8	2	-4.7
		5	X
		10	2.3*
		15	x
		20	x
Zn^{2+}	7	15	4.02
	8		-21.9
	9		-23.3

Table S2. Surface potential of precipitates formed during Bst catalysed RCA at different pH and cation concentration. The surface potential was determined using a Malvern Zetasizer Ultra instrument. Particles were suspended in 10 mM aqueous NaCl . x indicates that good quality data could not be obtained, this could be due to the particles being too large and sedimenting or unstable during the measurements. * Indicates samples prepared on different days and is an indication of batch to batch variability in the samples.

Cation	pH	cation concentration (mM)	Zeta potential (mV)
Mn^{2+}	7	10	-33.2
	7.5		-20.0
	8		-4.4*
	8.5		-6.6
	9		-8.8
	8	2	-16.6
		5	-4.6
		10	-5.0*
		15	-12.9
		20	x
Co^{2+}	7	10	-11.0
	7.5		-27.3*
	8		-30.3
	8.5		-31.4
	9		-5.2
	7.5	2	x
		5	x
		10	-27.7*
		15	-40.8
		20	x
Zn^{2+}	7	20	-25.9
	7.5		-19.9
	8		-23.8
	8.5		x
	9		x
	7	15	-1.3
	8		-27.7
	9		x

Table S3. Surface potential of precipitates formed during Φ29 catalysed RCA at different pH and cation concentration. The surface potential was determined using a Malvern Zetasizer Ultra instrument. Particles were suspended in 10 mM aqueous NaCl . x indicates that good quality data could not be obtained, this could be due to the particles being too large and sedimenting or unstable during the measurements. * Indicates samples prepared on different days and is an indication of batch to batch variability in the samples.

MnDNF and CoDNF serum stability assays

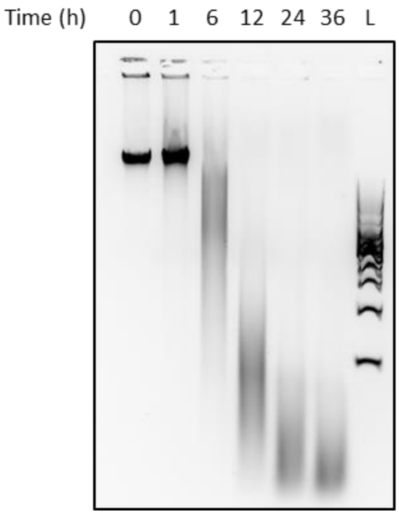

Figure S59. Double-stranded λ-DNA stability in DMEM medium substituted with 10% FBS at 37 °C. Left) agarose gel electrophoresis analysis after the incubation time indicated; right) ImageJ was used to measure the relative intensity compared to the distinctive band associated with non-treated λ-DNA. L) 1 kb DNA ladder.

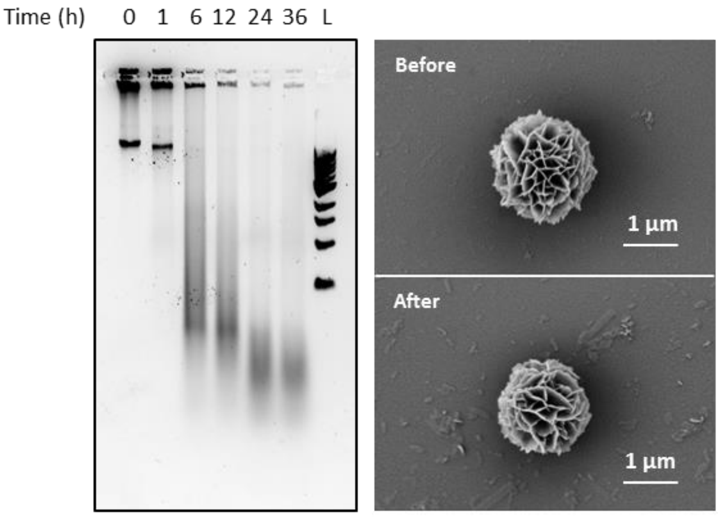

Figure S60. The stability of MnDNFs prepared using Φ29 with 10 mM $MnCl_2$ in DMEM medium substituted with 10% FBS at 37 °C. Left) agarose gel electrophoresis analysis after the incubation time indicated; right) SEM images of MnDNFs before and after 48 h incubation. L) 1 kb DNA ladder.

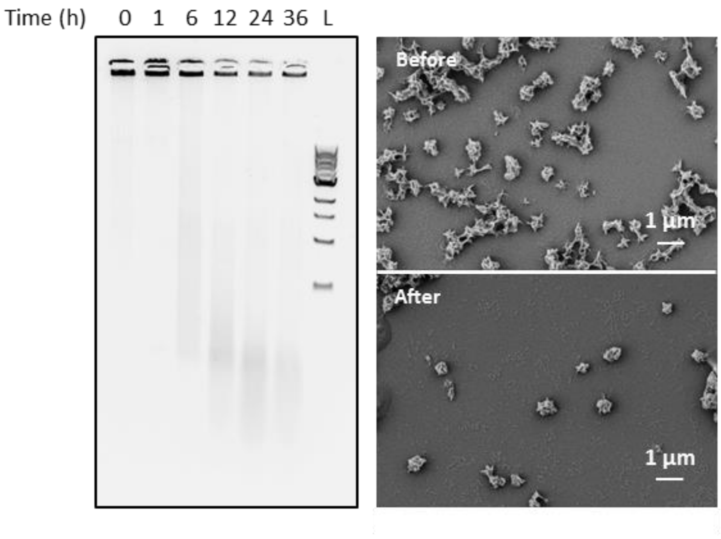

Figure S61. The stability of MnDNFs prepared using Φ29 with 15 mM $MnCl_2$ in DMEM medium substituted with 10% FBS at 37 °C. Left) agarose gel electrophoresis analysis after the incubation time indicated; right) SEM images of MnDNFs before and after 48 h incubation. L) 1 kb DNA ladder.

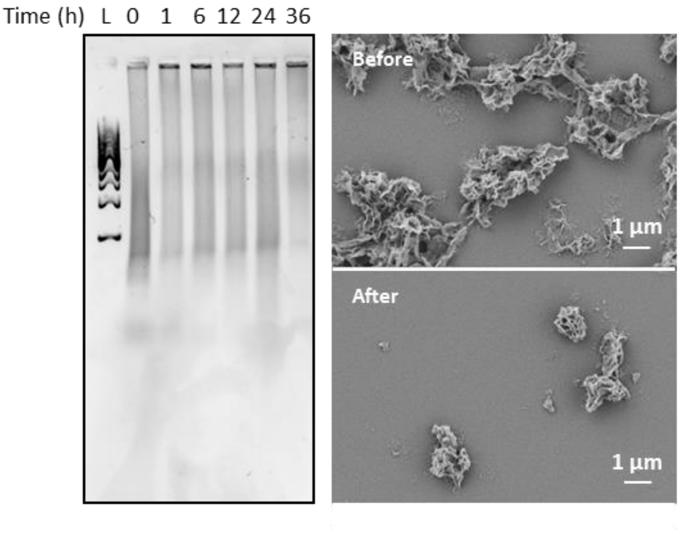

Figure S62. The stability of MnDNFs prepared using Bst with 10 mM $MnCl_2$ in DMEM medium substituted with 10% FBS at 37 °C. Left) agarose gel electrophoresis analysis after the incubation time indicated; right) SEM images of MnDNFs before and after 48 h incubation. L) 1 kb DNA ladder.

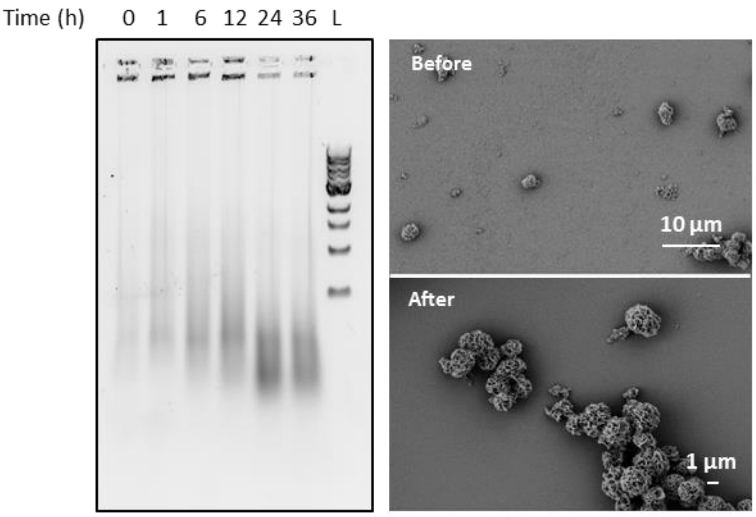

Figure S63. The stability of MnDNFs prepared using Bst with 15 mM $MnCl_2$ in DMEM medium substituted with 10% FBS at 37 °C. Left) agarose gel electrophoresis analysis after the incubation time indicated; right) SEM images of MnDNFs before and after 48 h incubation. L) 1 kb DNA ladder.

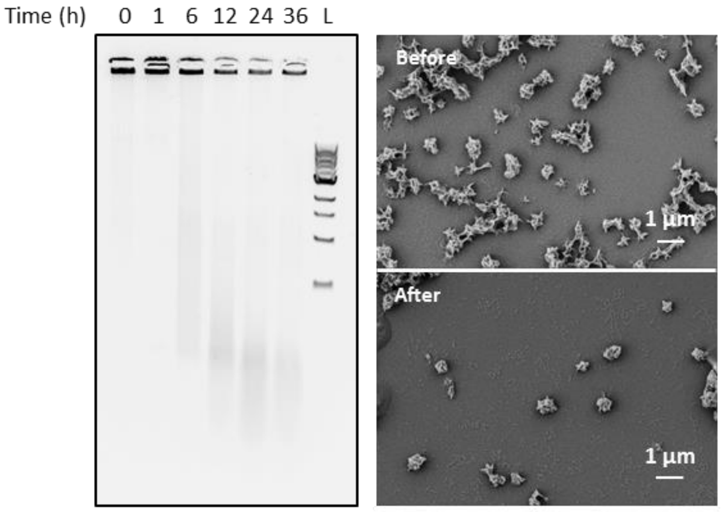

Figure S64. The stability of CoDNFs prepared using Φ29 with 10 mM $CoCl_2$ in DMEM medium substituted with 10% FBS at 37 °C. Left) agarose gel electrophoresis analysis after the incubation time indicated; right) SEM images of CoDNFs before and after 48 h incubation. L) 1 kb DNA ladder.

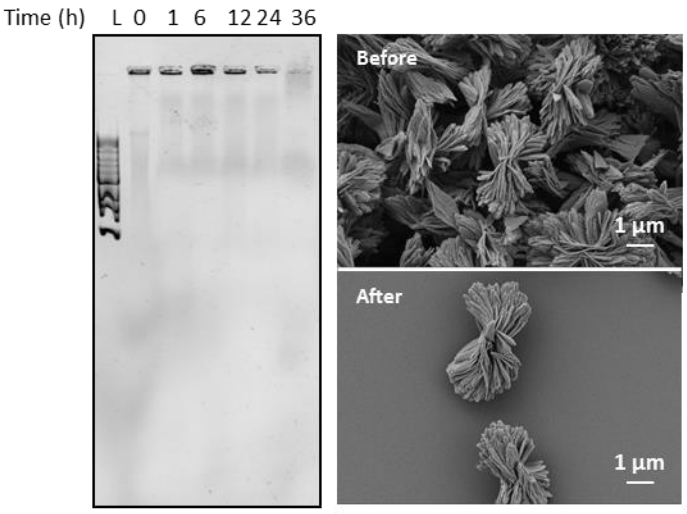

Figure S65. The stability of CoDNFs prepared using Φ29 with 15 mM $CoCl_2$ in DMEM medium substituted with 10% FBS at 37 °C. Left) agarose gel electrophoresis analysis after the incubation time indicated; right) SEM images of CoDNFs before and after 48 h incubation. L) 1 kb DNA ladder.

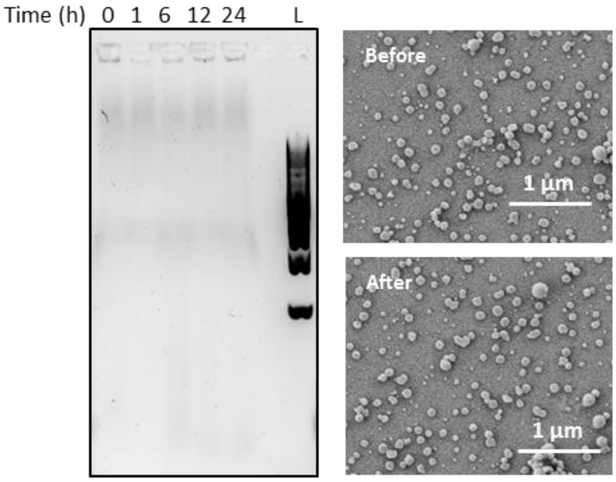

Figure S66. The stability of ZnDNFs prepared using Φ29 with 20 mM $ZnCl_2$ in DMEM medium substituted with 10% FBS at 37 °C. Left) agarose gel electrophoresis analysis after the incubation time indicated; right) SEM images of ZnDNFs before and after 48 h incubation. L) 1 kb DNA ladder.

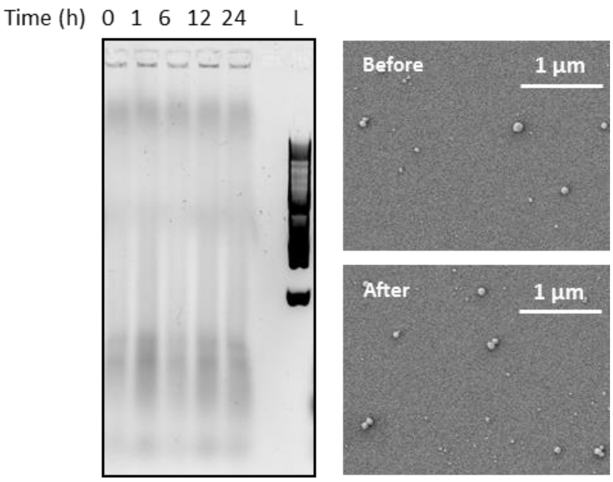

Figure S67. The stability of ZnDNFs prepared using Bst with 15 mM $ZnCl_2$ in DMEM medium substituted with 10% FBS at 37 °C. Left) agarose gel electrophoresis analysis after the incubation time indicated; right) SEM images of ZnDNFs before and after 48 h incubation. L) 1 kb DNA ladder.

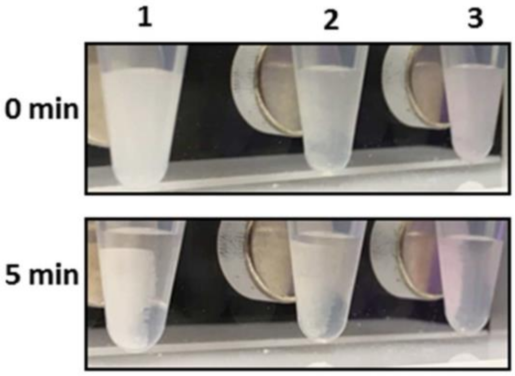

Figure S68. Capture of MnDNFs and CoDNFs using a commercial magnetic rack. The suspended particles are attracted to the magnets and the solution becomes clear within 5 min. 1) MnDNFs prepared using Bst with 15 mM $MnCl_2$; 2) MnDNFs prepared using Φ29 with 15 mM $MnCl_2$; 3) CoDNFs prepared using Φ29 with 15 mM $CoCl_2$.

Mass spectra of the DNA templates and splints used in this study

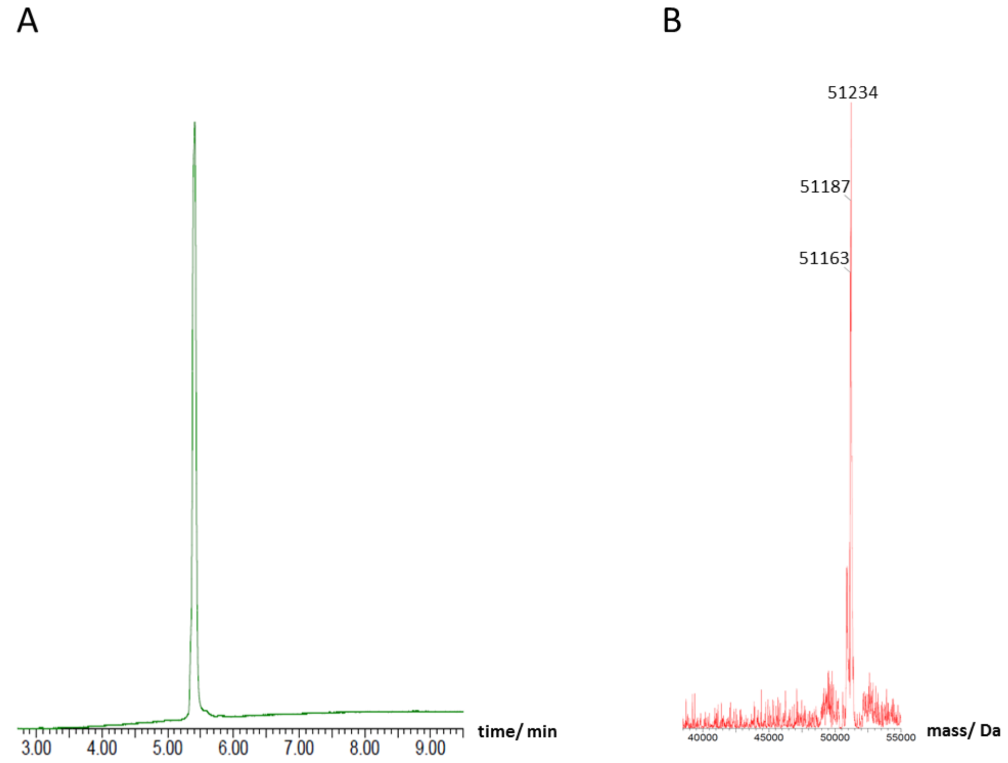

Figure S69. Reversed-phase HPLC (UV abs at 260 nm) and mass spectrum (ES^-) of circular template T1, required 51160 Da, found 51163 Da. The peaks show the product as the Na^+ adducts.

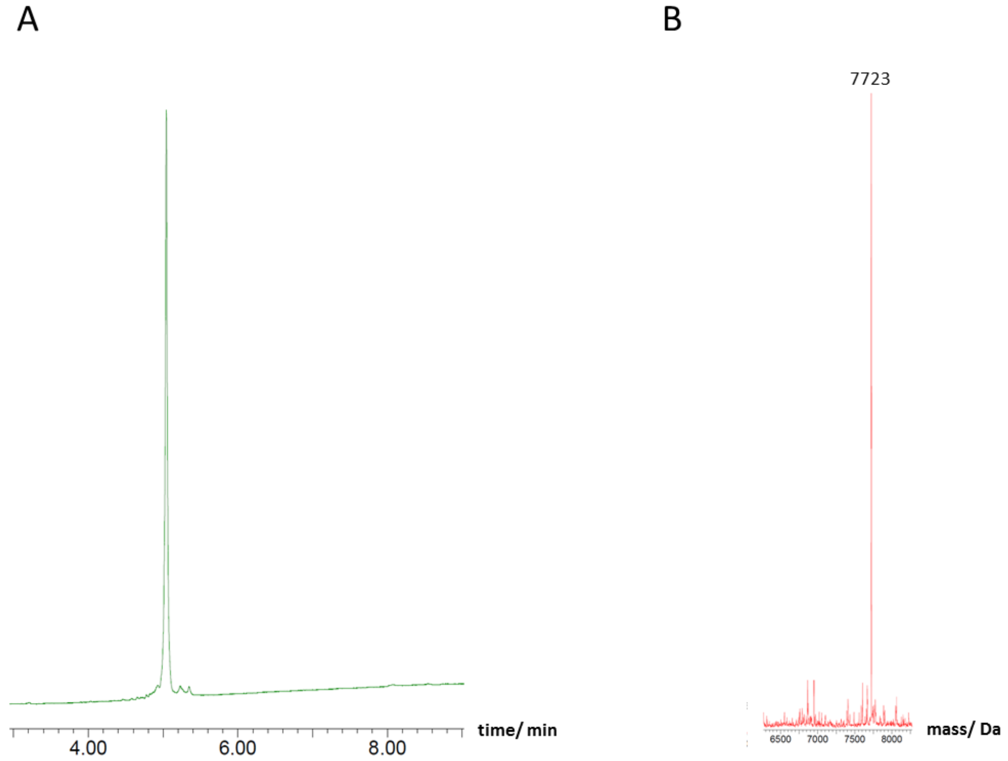

Figure S70. Reversed-phase HPLC (UV abs at 260 nm) and mass spectrum (ES^-) of S1, required 7724 Da, found 7723 Da.

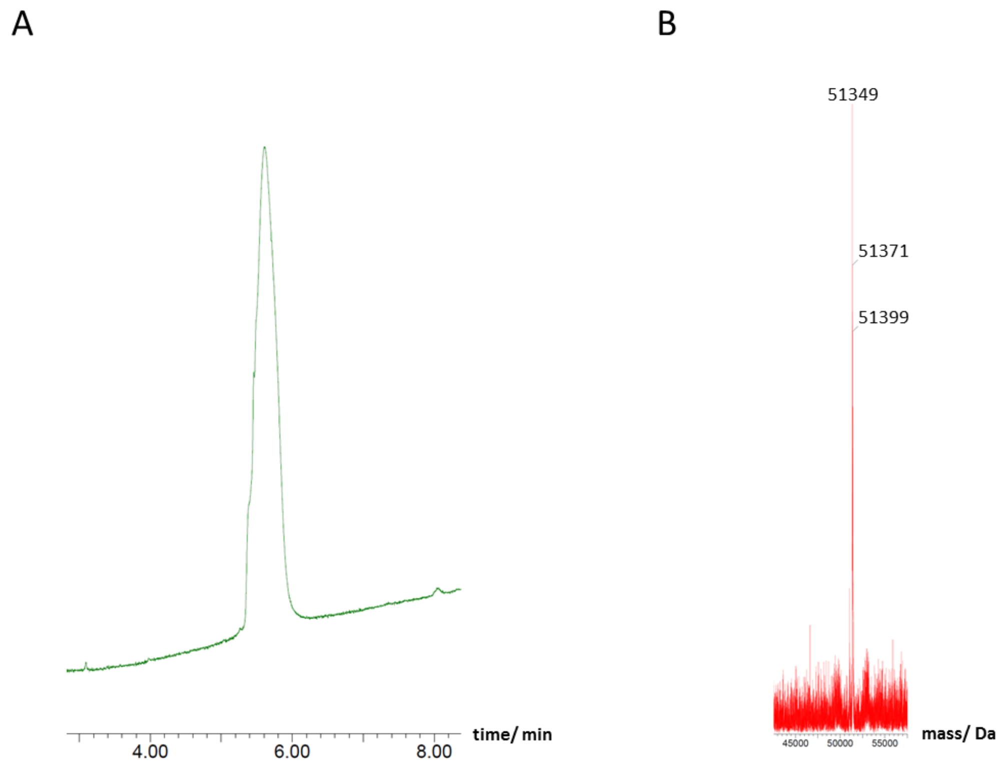

Figure S71. Reversed-phase HPLC (UV abs at 260 nm) and mass spectrum (ES$^-$) of circular template T2, required 51346 Da, found 51349 Da. The peaks show the product as the Na$^+$ adducts.

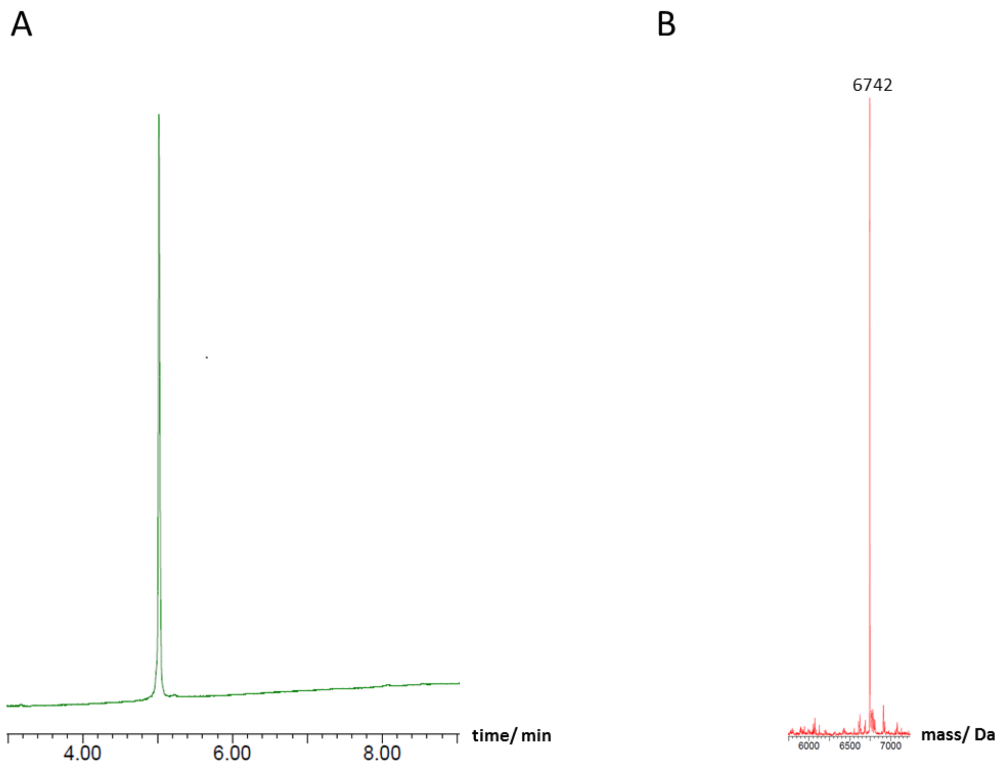

Figure S72. Reversed-phase HPLC (UV abs at 260 nm) and mass spectrum (ES$^-$) of S2, required 6742 Da, found 6742 Da.

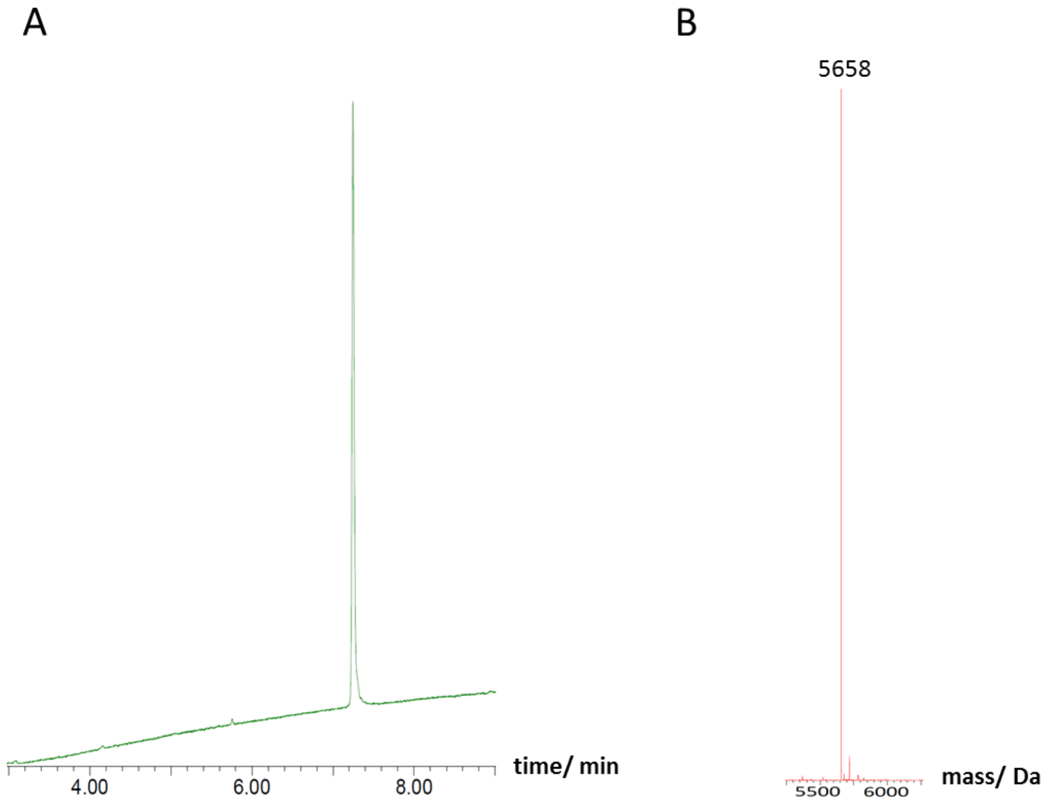

Figure S73. Reversed-phase HPLC (UV abs at 260 nm) and mass spectrum (ES$^-$) of match probe, required 5658 Da, found 5658 Da.

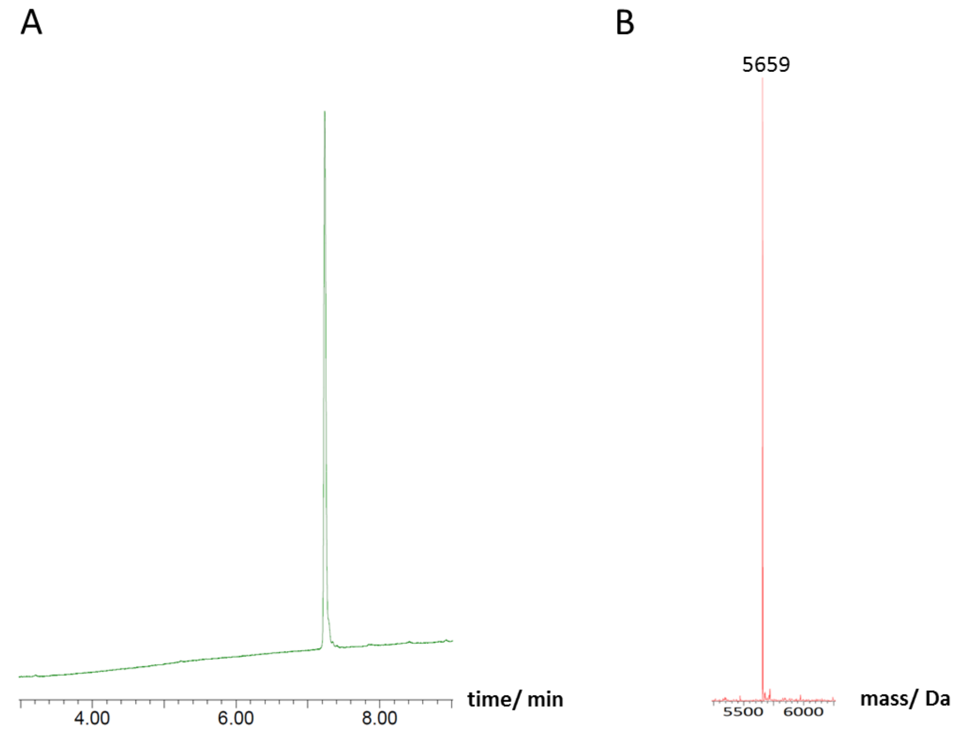

Figure S74. Reversed-phase HPLC (UV abs at 260 nm) and mass spectrum (ES$^-$) of mismatch probe, required 5658 Da, found 5659 Da.

References

1. Jin, K., Park, J., Lee, J., Yang, K.D., Pradhan, G.K., Sim, U., Jeong, D., Jang, H.L., Park, S., Kim, D. *et al.* (2014) Hydrated Manganese(II) Phosphate ($Mn_3(PO_4)_2 \cdot 3H_2O$) as a Water Oxidation Catalyst. *J. Am. Chem. Soc.*, **136**, 7435-7443.
2. Kim, E., Zwi-Dantsis, L., Reznikov, N., Hansel, C.S., Agarwal, S. and Stevens, M.M. (2017) One-Pot Synthesis of Multiple Protein-Encapsulated DNA Flowers and Their Application in Intracellular Protein Delivery. *Adv. Mater.*, **29**, 1701086.
3. Ducani, C., Bernardinelli, G. and Högberg, B. (2014) Rolling circle replication requires single-stranded DNA binding protein to avoid termination and production of double-stranded DNA. *Nucleic Acids Res.*, **42**, 10596-10604.
